# Vortex-Flow-Directed Chiral Macroscopic Ordering of Platelet Nanostructures Formed via the Supramolecular Assembly of Platinum Complexes with Bis(phenylisoxazolyl)benzene

**DOI:** 10.1021/jacs.5c03761

**Published:** 2025-08-13

**Authors:** Masaya Yoshida, Kyota Yasuda, Takuma Matsumoto, Yudai Ono, Naoyuki Hisano, Mao Kawasaki, Takehiro Hirao, Ye Yuan, Shin-ichi Tate, Martin Vacha, Takeharu Haino

**Affiliations:** † Department of Chemistry, Graduate School of Advanced Science and Engineering, 12803 Hiroshima University, 1-3-1 Kagamiyama, Higashi-Hiroshima, Hiroshima 739-8526, Japan; ‡ International Institute for Sustainability with Knotted Chiral Meta Matter (SKCM^2^), Hiroshima University, 1-3-1 Kagamiyama, Higashi-Hiroshima, Hiroshima 739-8526, Japan; § Program of Mathematical and Life Sciences, Graduate School of Integrated Sciences for Life, Hiroshima University, 1-3-1 Kagamiyama, Higashi-Hiroshima, Hiroshima 739-8526, Japan; ∥ Research Center for the Mathematics on Chromatin Live Dynamics, Hiroshima University, 1-3-1 Kagamiyama, Higashi-Hiroshima, Hiroshima 739-8526, Japan; ⊥ Department of Materials Science and Engineering, Institute of Science Tokyo, Ookayama 2-12-1-S8-44, Meguro-ku, Tokyo 152-8552, Japan

## Abstract

To understand the vortex flow-directed circular dichroism (CD) effect observed in homogeneous solutions containing supramolecular structures, the macroscopic order formed by supramolecular structures oriented within a flow must be visualized. In this study, a bis­(phenylisoxazolyl)­benzene-attached platinum complex was found to self-assemble to form uniform anisotropic platelet nanostructures that are oriented within a flow, thereby generating a chiral macroscopic order that is responsible for CD and linear dichroism (LD) effects only in the vortex flow regime. Cooperative self-assembly of a bis­(phenylisoxazolyl)­benzene-attached platinum complex via controlled supramolecular polymerization produced anisotropic platelet nanostructures with a narrow polydispersity index. The orientational order parameter of the nanostructures correlated with the flow velocity; thus, the nanostructures were oriented along the flow direction. Furthermore, the vortex flow of the dilute nanostructure solution broke the symmetry of the flow, thereby generating a chiral macroscopic order. As a result, CD and LD effects were observed in the vortex flow regime of the dilute nanostructure solution. These results can be generalized to the formation of chiral macroscopic order in solutions containing anisotropic nanostructures.

## Graphical abstract

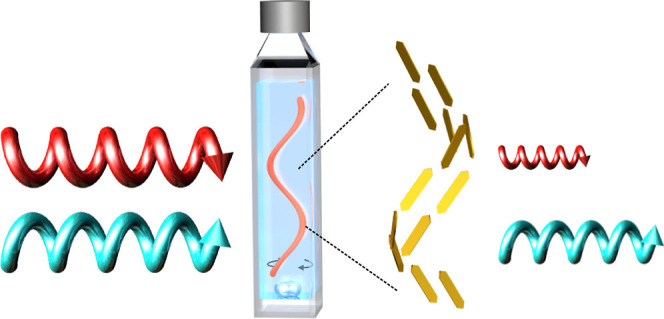

## Introduction

Vortex flow refers to the swirling motion observed in the flow of fluids commonly found in nature, including whirlpools, tornadoes, and hurricanes.^1^ The formation of vortices disrupts the flow symmetry, resulting in macroscale chirality, which translates into the chiral order of nanoscale objects such as metal nanoparticles, nanorods, polymers, and DNA.^2–8^ Typically, this macroscale chirality does not influence the molecular chirality in dilute molecular solutions because Brownian motion generally dominates over hydrodynamic effects. However, Ribó et al. reported that a stirring-directed vortex flow led to the emergence of chirality in the aggregation of an achiral porphyrin upon concentration.^9^ In this system, the direction of vortex flow dictated the chirality of the resulting porphyrin aggregates, resulting in spontaneous symmetry breaking. Therefore, the sign of the observed circular dichroism (CD) signal depended on the vortex flow direction. Notably, the induced chirality remained stable even after stirring was terminated. The authors proposed that vortex-flow-induced irreversible supramolecular chirality is imparted by a kinetically controlled chiral selection mechanism in the supramolecular assembly process of an achiral porphyrin,^10–12^ where the initial nucleation events yield a slight enantiomeric bias, which is amplified during aggregate growth in the vortex flow regime. This chiral preference is irreversibly memorized in each aggregate, yielding a persistent asymmetric aggregate population. This discovery prompted the investigation of persistent supramolecular chirality induction by the aggregation of achiral molecules under vortex flow conditions.^13–27^

In contrast to the persistent supramolecular chirality generated by the kinetically controlled chiral selection mechanism, Aida and Meijer independently reported that dilute solutions of achiral supramolecular polymers exhibit a nonequilibrium CD effect in the vortex flow regime upon stirring.^28,29^ These CD responses were sensitive to the vortex flow, and thus, when the vortex flow stopped, the CD response disappeared. In addition, they found that the sign and intensity of the CD response depended on the direction and rate of the vortex flow. Consequently, they claimed that the stirring-directed vortex flow resulted in a flow-oriented structure for supramolecular polymers, which in turn induced the CD effects attributed to contributions from linear dichroism (LD) and linear birefringence (LB) effects. Vortex-flow-induced nonequilibrium CD responses have been investigated across various supramolecular aggregates, reinforcing the hypothesis that asymmetric hydrodynamic forces generated by vortex flow induce nonequilibrium chiral macroscopic ordering of supramolecular aggregates^30–35^ and, extremely rarely, induce transient helical elastic deformation.^36–39^ Thus, revealing the structural characteristics of the nonequilibrium chiral macroscopic orders underlying the observed nonequilibrium CD effects has become a primary scientific interest.^40–42^ Previous studies primarily relied on bulk spectroscopic techniques to deduce the presence of nonequilibrium chiral macroscopic orders. The precise structural characteristics of nonequilibrium chiral macroscopic orders, such as the anisotropic features of each supramolecular aggregate and the spatial arrangement and orientation of moving supramolecular aggregates within a flow, have not been elucidated in real-space resolution. The direct real-space visualization of the macroscopic orders of moving supramolecular aggregates under flow conditions, as well as position-resolved mapping of their absorption anisotropy, remain indispensable for understanding the vortex-flow-induced CD effects; currently, this is an extremely challenging task.

In this decade, we have studied the self-assembly of phenyl bipyridine platinum­(II) complexes with isoxazole groups into supramolecular nanostructures exhibiting aggregation-induced emission enhancement (AIEE).^43–46^ It is envisioned that the AIEE active nanostructures of these platinum complexes could be exploited to directly visualize the macroscopic order controlled by a vortex flow in solution. With this in mind, the orientation of anisotropic platelet supramolecular nanostructures formed by the self-assembly of achiral platinum complex **1** is examined in a vortex-flow regime, and its potential to generate a chiral macroscopic order that develops the CD effect is considered (Figure 1). The absorption anisotropy of the platelet supramolecular nanostructures is determined using fluorescence microscopy, while the flow-directed ordering of the platelet supramolecular nanostructures is visualized using confocal laser scanning microscopy (CLSM). Finally, the role of the vortex flow in generating a helical macroscopic order in the supramolecular nanostructures is evaluated, in addition to its influence on generating CD and LD effects in the vortex flow.

**Figure 1:**
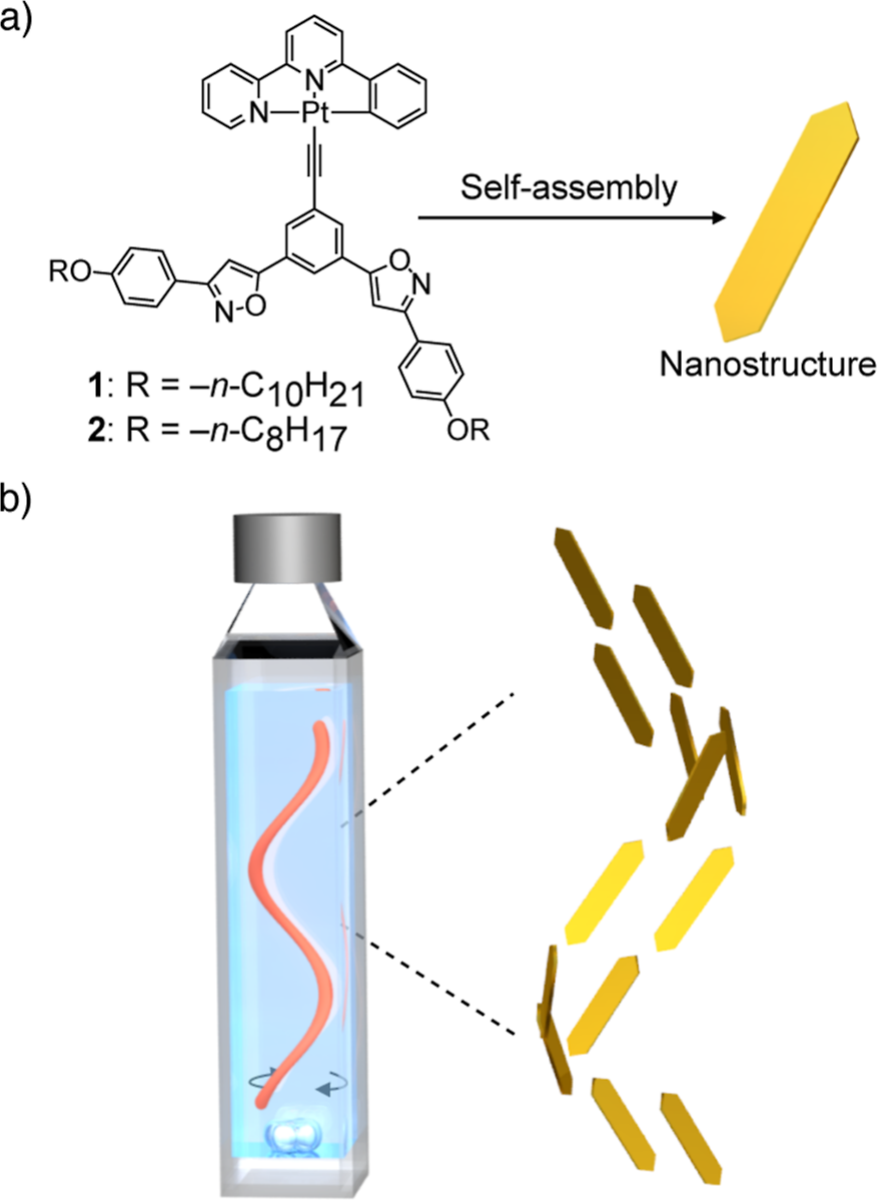
(a) Molecular structures of **1** and **2**. Formation of a supramolecular nanostructure via the self-assembly of **1**. (b) Illustration of the helical ordering of nanostructures in the vortex flow induced by stirring in a sample cuvette.

## Results and Discussion

The self-assembly behavior of **1** in toluene was assessed using ultraviolet–visible (UV–vis) absorption spectroscopy (Figure 2a). Heating a solution of **1** resulted in a gradual decrease in the absorption bands at 452 nm, whereas a new band emerged at 480 nm, with an isosbestic point at 473 nm. The heating curve for the degree of aggregation (α) at 420 nm resulted in a nonsigmoidal melting curve, characterizing the cooperative self-assembly of **1** (Figure 2b).^47^ Thermodynamic insights into the cooperative self-assembly were obtained by applying the van der Schoot model (Figures S1 and S2),^48^ providing an elongation temperature (*T*_e_) of 334.2(1) K at the studied concentration, and resulting in an association constant (*K*_e_) of 4.2(3) × 10^5^ M^–1^. The dimensionless equilibrium constant (*K*_a_) of 6.1(6) × 10^–4^, which was defined for the nucleation-to-elongation transition, is less than unity, thereby indicating that the supramolecular assembly of **1** is a highly cooperative process. The degree of polymerization (*N*_n_(*T*_e_)) was determined to be 11.8(4) at the elongation temperature, which agreed with the size of the nucleus at the nucleation–elongation transition. A thermodynamic study of the cooperative self-assembly established an enthalpy change of −56.5(1) kJ mol^–1^ and an entropy change of −101.5(4) J mol^–1^ K^–1^, indicating that the cooperative self-assembly of **1** is a common enthalpy-driven and entropy-opposed process (Figures S1 and S2).

**Figure 2:**
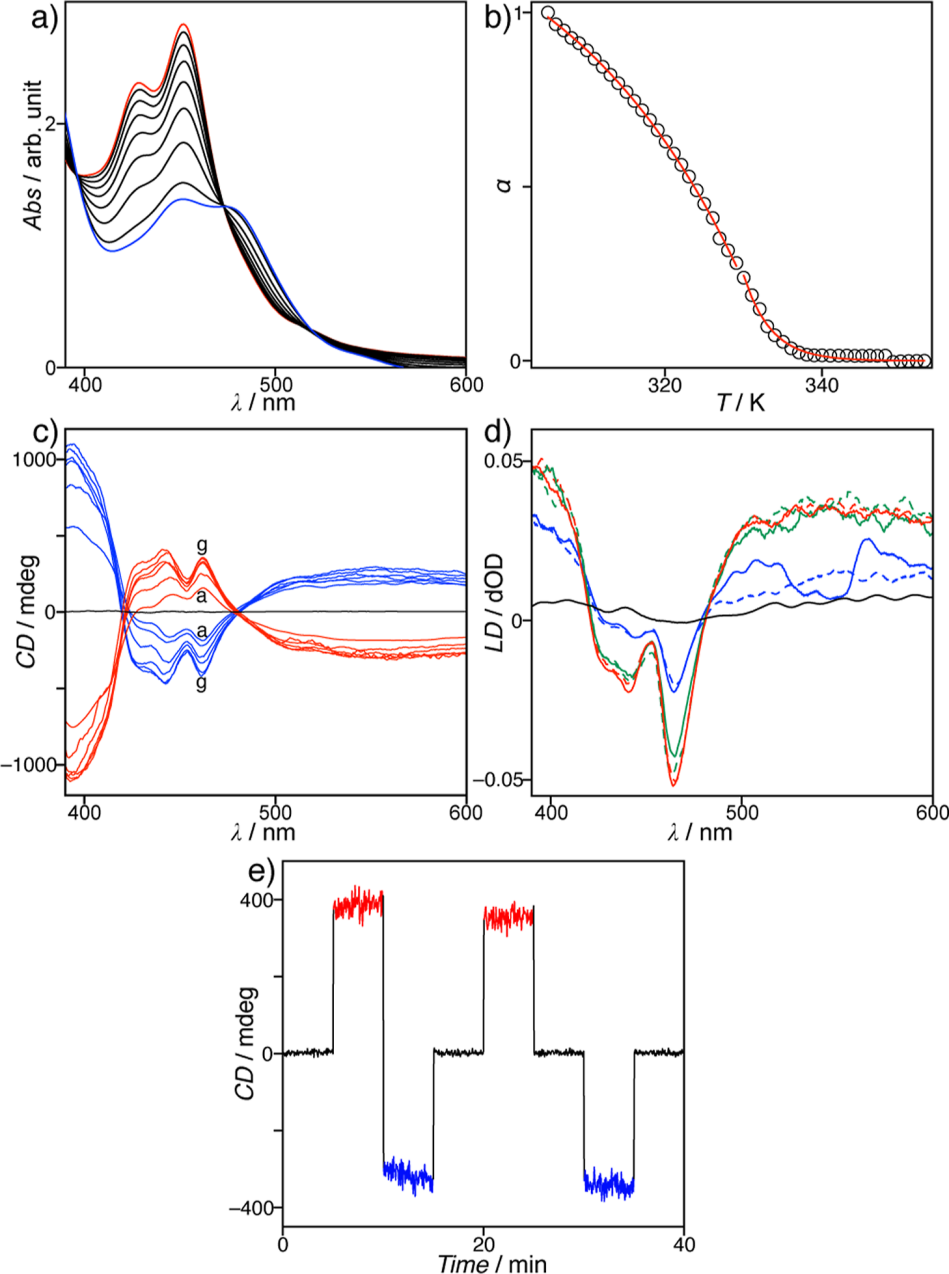
(a) UV–vis spectra of **1** (3.0 × 10^–4^ M in toluene) upon heating at a rate of 1 K min^–1^: (from red to blue) 298, 303, 308, 313, 318, 323, 328, 333, and 338 K. (b) Plot representing the degree of aggregation (α) as a function of temperature. All points were obtained from the UV–vis spectra presented in panel (a). (c) CD spectra of **1** (3.0 × 10^–4^ M in toluene) at 298 K without stirring (black curve) and with stirring: (from a–g) 100, 200, 300, 500, 900, and 1000 rpm for CW (red curve) and CCW (blue curve) rotations. (d) LD spectra recorded in toluene at 298 K with stirring at 0 rpm (black curve), 100 rpm (blue curves), 500 rpm (green curves), and 1000 rpm (red curves) for CW (solid curves) and CCW (dashed curves) rotations. (e) CD responses recorded at 444 nm with stirring at 0 rpm (black curves) and 1000 rpm for CW (red curves) and CCW (blue curves) rotation.

Since the supramolecular aggregates of achiral compound **1** were fundamentally achiral, the solution of **1** at 298 K was CD-silent without stirring. The absence of LD indicated that no specific macroscopic order was formed in the solution. However, clockwise (CW) stirring (100 rpm) of a toluene solution of **1** in a 1 cm square sample cuvette produced a negative–positive–negative trisignate CD signal with a strong LD signal, while counterclockwise (CCW) stirring produced a mirror-image CD signal (Figures 2c,d, and S3). The CD and LD signals were enhanced upon increasing the stirring rate, but disappeared immediately when stirring was stopped. Furthermore, the CD response at 444 nm was reproducible without delay when the stirring direction was reversed (Figure 2e). Notably, no significant changes were observed in the UV–vis absorption spectra of the solution in the presence or absence of stirring (Figure S4), thereby indicating that the solution homogeneity was maintained under both conditions.

The formation of supramolecular aggregates of **1** is sensitive to the solution temperature; therefore, the temperature-dependent CD spectra of **1** can provide crucial details for understanding this stirring-directed CD induction behavior. Intense CD signals were maintained during stirring at 1000 rpm and 298 K in both the CW and CCW rotational directions. Upon heating the solution, the CD signals gradually decreased until the temperature reached 338 K (Figures 3a and S5). Subsequently, heating curves reflecting the degree of aggregation were generated based on the CD intensity at 442 nm for both CW and CCW rotations. The changes in the CD intensity presented in Figure 3b reflect the typical nucleation–elongation behavior occurring in both rotational directions, which is in good agreement with the UV–vis absorption results. The elongation temperatures (*T*_e_) for the CW and CCW rotational directions were determined to be 331.8(1) and 329.5(3) K, respectively (Figure S5), which coincides with the *T*_e_ value determined by UV–vis absorption spectroscopy. When the solution temperature was lowered in the elongation regime, the CD intensity correlated with the degree of aggregation (α). Accordingly, the supramolecular aggregates of **1** are clearly responsible for the induction of CD signals under CW and CCW rotation, which are unique in the elongation region.

**Figure 3:**
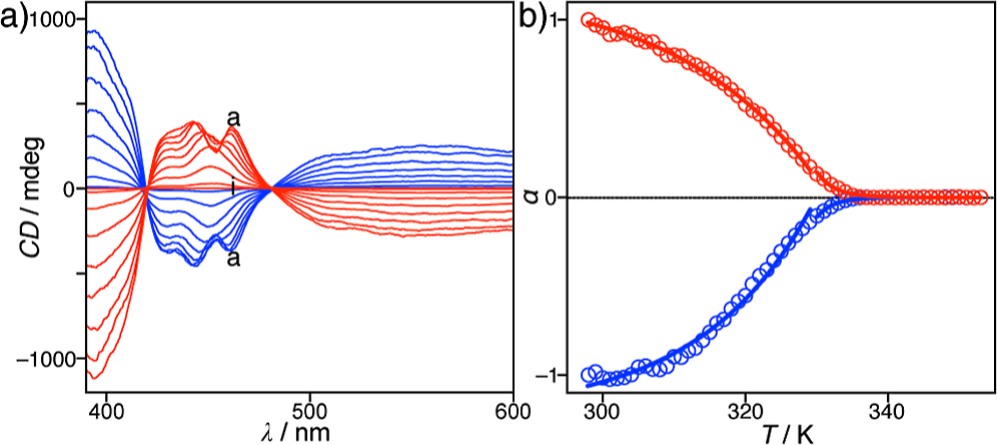
(a) CD spectra of **1** (3.0 × 10^–4^ M in toluene) recorded at different temperatures: (from a–i) 298, 303, 308, 313, 318, 323, 328, 333, and 338 K with stirring at 1000 rpm for CW (red curve) and CCW (blue curve) rotations. (b) Plots of the degree of aggregation (α) during CW (red) and CCW (blue) rotation as a function of the temperature. All points were obtained from the CD spectra of **1** (3.0 × 10^–4^ M) recorded in toluene.

Atomic force microscopy (AFM) and transmission electron microscopy (TEM) were subsequently employed to reveal the structure of the supramolecular aggregate of **1**. For this purpose, a toluene solution of **1** (3.0 × 10^–4^ M) at 298 K was spin-coated on a mica plate and deposited on a copper grid with lacy carbon. The AFM image confirmed the presence of uniform platelet nanostructures, consistent with those observed in the TEM image (Figures 4a,b, S7, and S8). In addition, the sizes, shapes, and dimensions of the nanostructures were precisely evaluated using the AFM images. More specifically, the number-average length (*L*_n_) and weight-average length (*L*_w_) of the principal axis of the nanostructure were determined to be 563 and 600 nm, respectively, giving a polydispersity index (PDI) of 1.06. The subordinate axis was also uniform, with *L*_n_ and *L*_w_ values of 40 and 43 nm, respectively, and a PDI of 1.08 (Figure S7, Tables S1 and S2). The PDI values for the principal and subordinate axes were close to unity, indicating that narrowly dispersed supramolecular nanostructures were formed. Thus, the cooperative self-assembly of **1** belonged to the class of living supramolecular polymerization.^49–51^ Furthermore, an aspect ratio of 14.1 between the principal and subordinate axes indicates that the nanostructures adopt diamond-shaped platelet structures. In contrast, amorphous films were formed on both the mica and copper grids when a solution of **1** was deposited at 353 K, indicating that monomeric **1** randomly agglomerated on these grid materials (Figures 4c,d, and S9). It is therefore evident that anisotropic platelet nanostructures are required to induce CD signals upon stirring.

**Figure 4:**
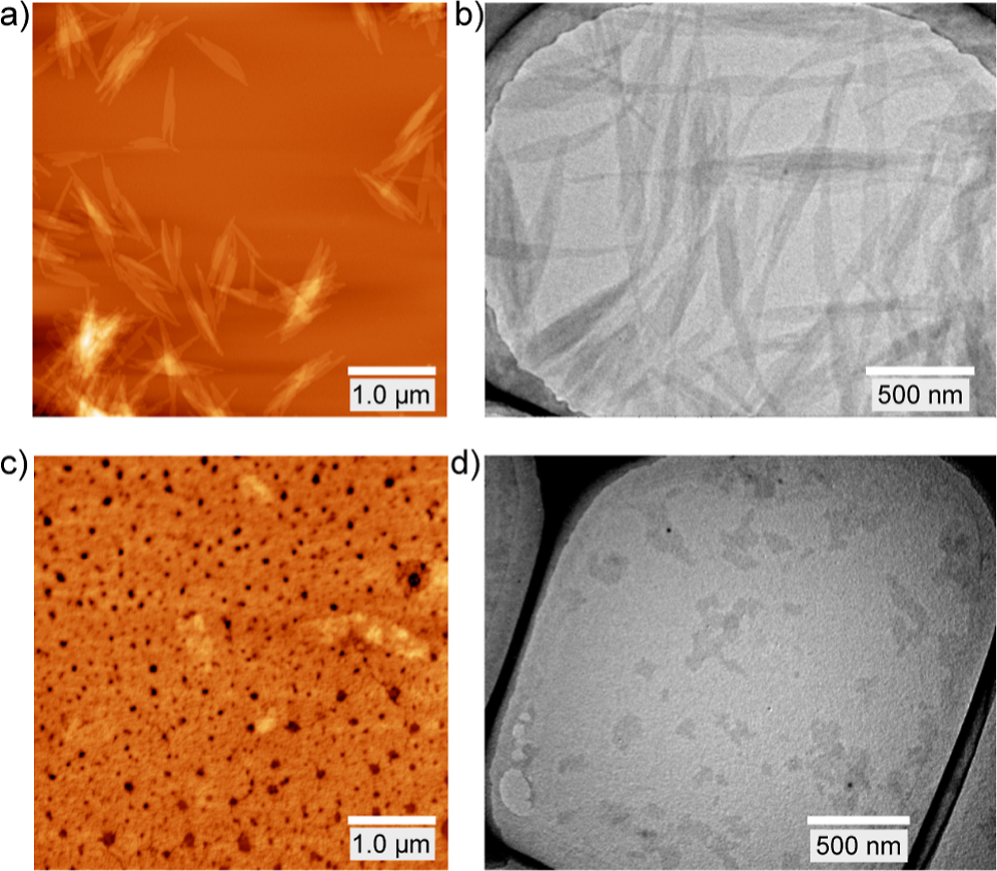
(a,c) AFM (5 μm × 5 μm) and (b,d) TEM images of **1** on a mica plate and on a copper grid with lacy carbon. A solution of **1** (3.0 × 10^–4^ M in toluene) was spin-coated onto a mica plate for AFM, and drop-cast onto a copper grid with lacy carbon for TEM at: (a,b) 298 K and (c,d) 353 K.

To study the sizes and concentrations of the nanostructures, dynamic light scattering (DLS) analysis of a toluene solution of **1** (3.0 × 10^–4^ M) was performed over a temperature range of 298–338 K (Figure S10a–c). At temperatures below 333 K, the *Z*-average hydrodynamic diameter remained nearly constant at approximately 560 nm, which closely matches the average size of the nanostructures determined by AFM. Above 338 K, however, no significant nanostructures were detected by DLS, indicating that the nanostructures were disassembled. The particle number concentration of the nanostructures remained approximately constant at an average of 1.23 × 10^10^ mL^–1^ up to 313 K, but exhibited a gradual decrease at temperatures exceeding 318 K, coinciding with the onset of the decrease in the CD intensities observed at approximately 310 K­(Figure S10d). Thus, these results suggest that the concentrations of the nanostructures in solution govern the magnitude of the stirring-induced CD intensity.

To study the contribution of the nanostructures to the CD effect under stirring, the anisotropic optical properties of the nanostructures were studied using a modified fluorescence microscope (Figures 5a and S10). The degree of absorption anisotropy is defined as *D*_ex_ = (*I*_max_ – *I*_min_)/(*I*_max_ + *I*_min_), where *I*_max_ and *I*_min_ are the maximum and minimum fluorescence intensities, respectively. In addition, absorption anisotropy measurements were employed to reveal the conformations of the supramolecular chains and particle shapes that were smaller than the resolution of fluorescence microscopy.^52–54^ After casting a solution of AIEE-active **1** on a glass substrate (Figure S11), the nanostructures were excited using a He–Cd laser (λ = 442 nm). The fluorescence intensities of the nanostructures on the glass substrate were then individually measured using an electron-multiplying charge-coupled device camera (Figures 5a, and S13–S17). As shown in Figure 5b, the anisotropic structures of the nanostructures were clearly visible. The polarization direction of the excitation light was therefore rotated continuously using a motor, and the fluorescence intensity of the nanostructure at each orientation angle of the linearly polarized excitation was measured to calculate the absorption anisotropy. The plot of the fluorescence intensity of the nanostructure versus the orientation angle of the excitation resulted in periodic modulation, which fit well with the cos^2^ function, confirming that the nanostructure possesses absorption anisotropy (Figures 5b,c, and S18–S21). In two representative examples, the directions of the absorption maxima are tilted from the principal axes of the nanostructures by the angles of ∠−29° and ∠−59°. These angles reflect the complex mesoscale hierarchical structure rather than molecular-level orientation of **1**. The absorption anisotropies of a total of 59 nanostructures were examined on the glass substrate (Figure 5d), and the results indicated that the nanostructures were anisotropic in absorption, with an average value *D̅*_ex_ of 0.059.

**Figure 5:**
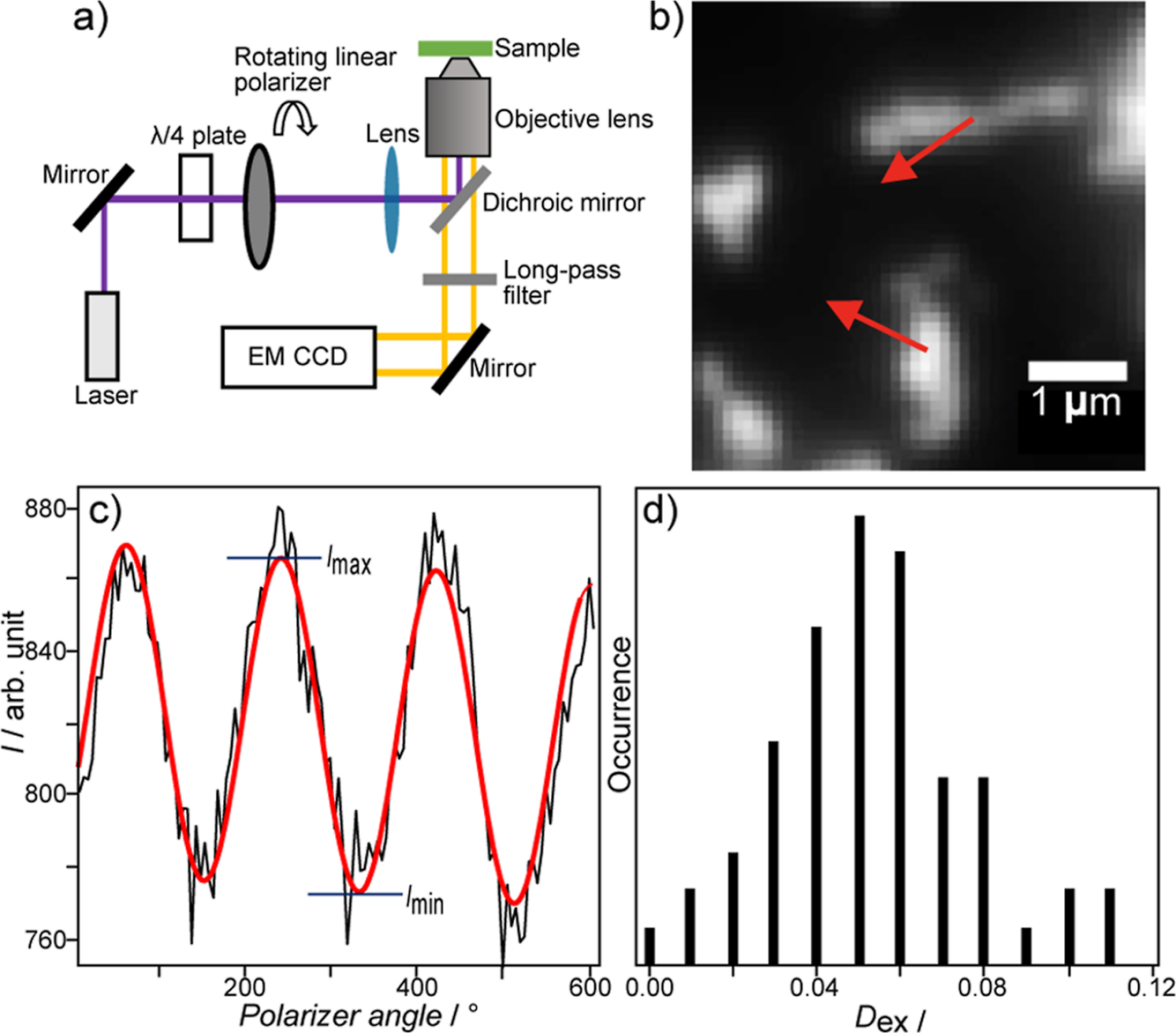
(a) Schematic illustration of the experimental setup used to measure the absorption anisotropy. (b) Microscopic image of emission from the nanostructures. The red arrows indicate the direction of maximum absorption. (c) An example of the change in emission intensity versus the orientation angle of the excitation light (black). The red line represents a cos^2^ fit of the data. (d) Histogram of the absorption anisotropy.

Powder X-ray diffraction (PXRD) analysis was performed to evaluate the crystallinity of **1**, revealing that the precipitates exhibited moderate crystallinity (Figure S22). Although single crystals of **1** could not be obtained, its analog **2** was crystallized by the slow diffusion methanol into a dichloromethane solution (Figure 1). A single crystal of **2** exhibited a long, needle-like shape (Figure S23a). X-ray crystallographic analysis revealed that a single crystal of **2** was in the triclinic unit cell with space group P1̅. The asymmetric unit comprises two molecules of **2** arranged in an antiparallel fashion, exhibiting effective π–π stacking interactions between the phenylbipyridine units (Figure 6a). The dimeric structures were grown along the major axis of the single crystal with an inclination angle of 45° (Figures 6b, S23, and S24).

**Figure 6:**
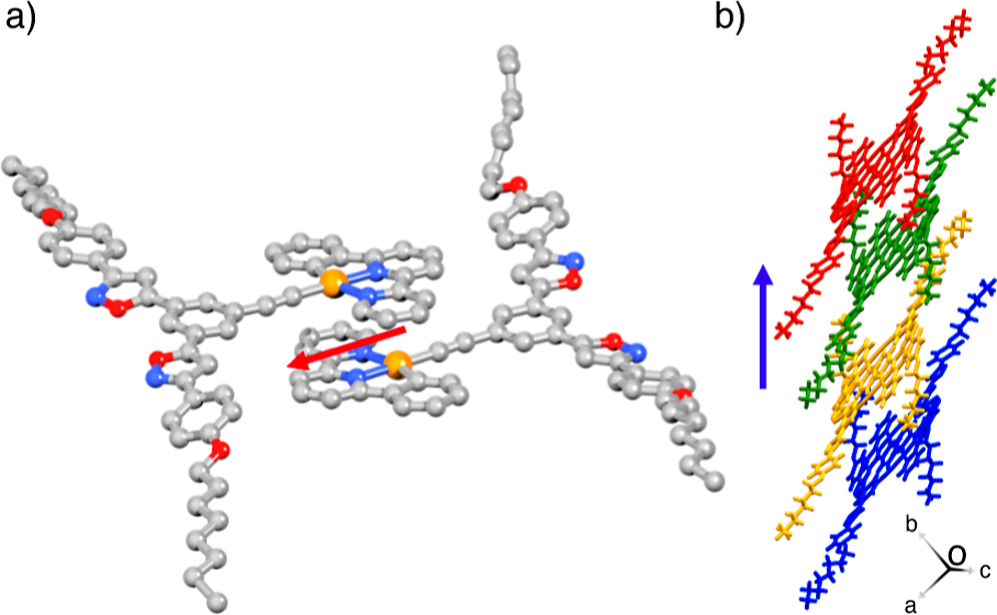
(a) Crystal structure of **2** arranged in an antiparallel fashion in the asymmetric unit. The hydrogens are omitted for clarity. The red arrow indicates the direction of the electronic transition dipole at 446.0 nm. (b) Stacked structure of the dimeric forms along the major axis (blue arrow) of the crystal.

To interpret the electronic absorption spectra of the dimeric crystal structure **2**•**2**, time-dependent density functional theory (TD-DFT) calculations at the M06/6–31G** + LanL2DZ level of theory were conducted without geometry optimization. Two major electronic transitions at 446.0 and 460.6 nm were identified in the visible region, originating from intra- and intermolecular charge-transfer electron promotions (Figures S25–S27, and Tables S4–S6). The transition dipole moments are located along the major molecular axes (Figures 6a and S27), thereby defining the direction of the absorption anisotropy. Assuming that **1** adopts a crystal structure analogous to that of **2**, the absorption anisotropies observed in the nanostructures could originate from the tilted orientation of **1** relative to the major axes of the nanostructures.^55^

Direct visualization of the macroscopic order of the anisotropic nanostructures in the flow is potentially informative for elucidating the dissipative CD effect in a vortex flow. In the current system, the intense emission characteristics of the nanostructures allowed visualization of their orientations in the flow using CLSM. In the flow direction (i.e., from the top right to the bottom left of each image), the movement and direction of the nanostructure were captured by time-lapse measurements under 488 nm excitation (Figures 7a–h and S28–S31, and Movies S1, S2, S3 and S4). In general, ellipsoidal particles are known to orient their long axes along the flow direction.^56^ In Figure 7a, each anisotropic platelet nanostructure is shown as a rectangular white dot. A time interval (Δ*t*) of 200 ms was used between slices to determine the nanostructure velocity. Using a flow with a velocity of 44 μm s^–1^, the four images presented in Figure 7a–d, which were recorded at 200 ms intervals, indicate that the nanostructures move with the flow direction, generating a highly oriented nanostructure ordering along the flow. In contrast, a less-ordered nanostructure orientation was obtained upon reducing the flow velocity (Figure 7e–h). Subsequently, the positions of the nanostructures at *t*_*n*_ and *t*_*n*+1_ were used to create a vector, and the angle of the particle at *t*_*n*+1_ relative to that of the vector was determined as the orientation angle (θ) (Figure 7i). Furthermore, the extent of the order in the flow can be evaluated by obtaining the two-dimensional orientational order parameter *S* = ⟨2 cos^2^ θ – 1⟩ ^57,58^ based on the orientation angle (θ) in the nanostructure displacement at each 200 ms interval (Tables S9, S11, S13, and S15). Since the order parameter of the orientation angle approaches unity as the flow velocity increases (Figure 7j), thereby indicating that the nanostructures are clearly oriented in the flow direction, generating a macroscopic order along the flow.

**Figure 7:**
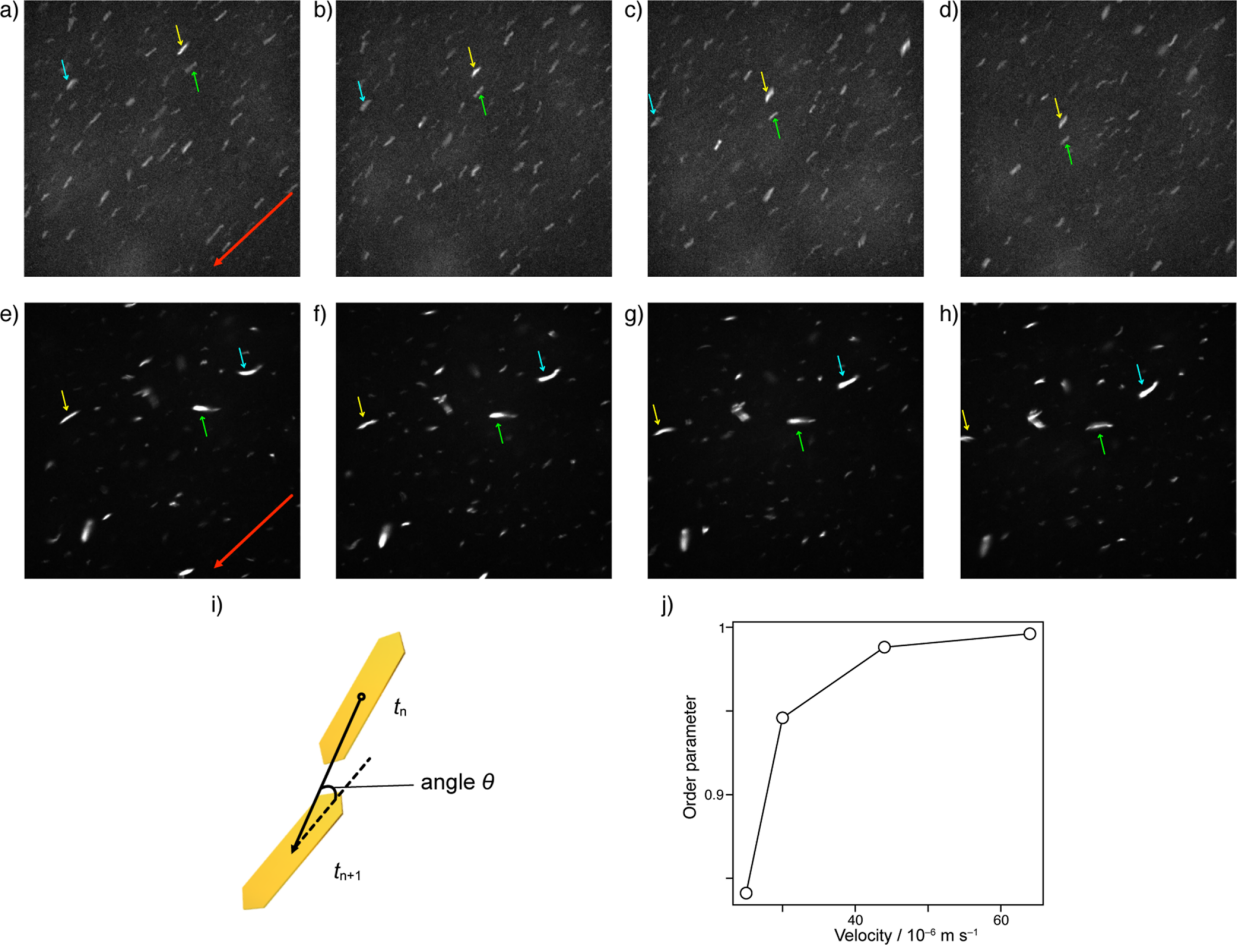
(a–h) Time-lapse CLSM images (82 μm × 82 μm) of a toluene solution of **1** (3.0 × 10^–4^ M) under the flow conditions (λ_ex_ = 488 nm): (a–d) in a velocity of 44 μm s^–1^; (e–h) in a velocity of 25 μm s^–1^. The red arrow represents the flow direction. The yellow, blue, and green arrows indicate the position of the same particles at each time interval. (i) Definition of the angle θ of the principal axis with respect to the displacement vector from *t*_*n*_ to *t*_*n*+1_. (j) Plot of the order parameter of the molecular aggregates to the flow direction versus the velocity of the molecular aggregates.

The induction of the CD effect by stirring in a 1 cm sample cuvette requires a vortex flow that generates chiral macroscopic ordering of the anisotropic nanostructures. Therefore, visualization of the vortex flow by stirring in a 1 cm sample cuvette was attempted by continuously injecting blue ink into ethanol using a syringe pump. When CW stirring was applied at 600 rpm, the ink trail exhibited a right-handed vortex flow at the center of the sample cuvette (Figure 8a). The vortex flow velocities were enhanced by increasing the stirring rate (Table S16). While the vortex flow at the center of a sample cuvette showed instability at a stirring rate of 300 rpm, subsequent increases in stirring rate resulted in an upward expansion of the vortex, thereby stabilizing the vortex flow at the center of a sample cuvette (Movies S5, S6, S7 and S8). Thus, the stirring rates are positively correlated with the CD intensities. However, the vortex flow completely disappeared at the bottom of the cuvette and the flow ran horizontally toward the cuvette wall, presumably without any chiral order (Figure 8b). In other words, the macroscopic chirality generated by the vortex flow was most likely enhanced at the center of the sample cuvette, and gradually disappeared upon moving toward the bottom of the cuvette. Due to the fact that the anisotropic platelet nanostructures were oriented in the flow direction, the CD and LD effects were sensitive to the position of the incident light (Figure 8c).

**Figure 8:**
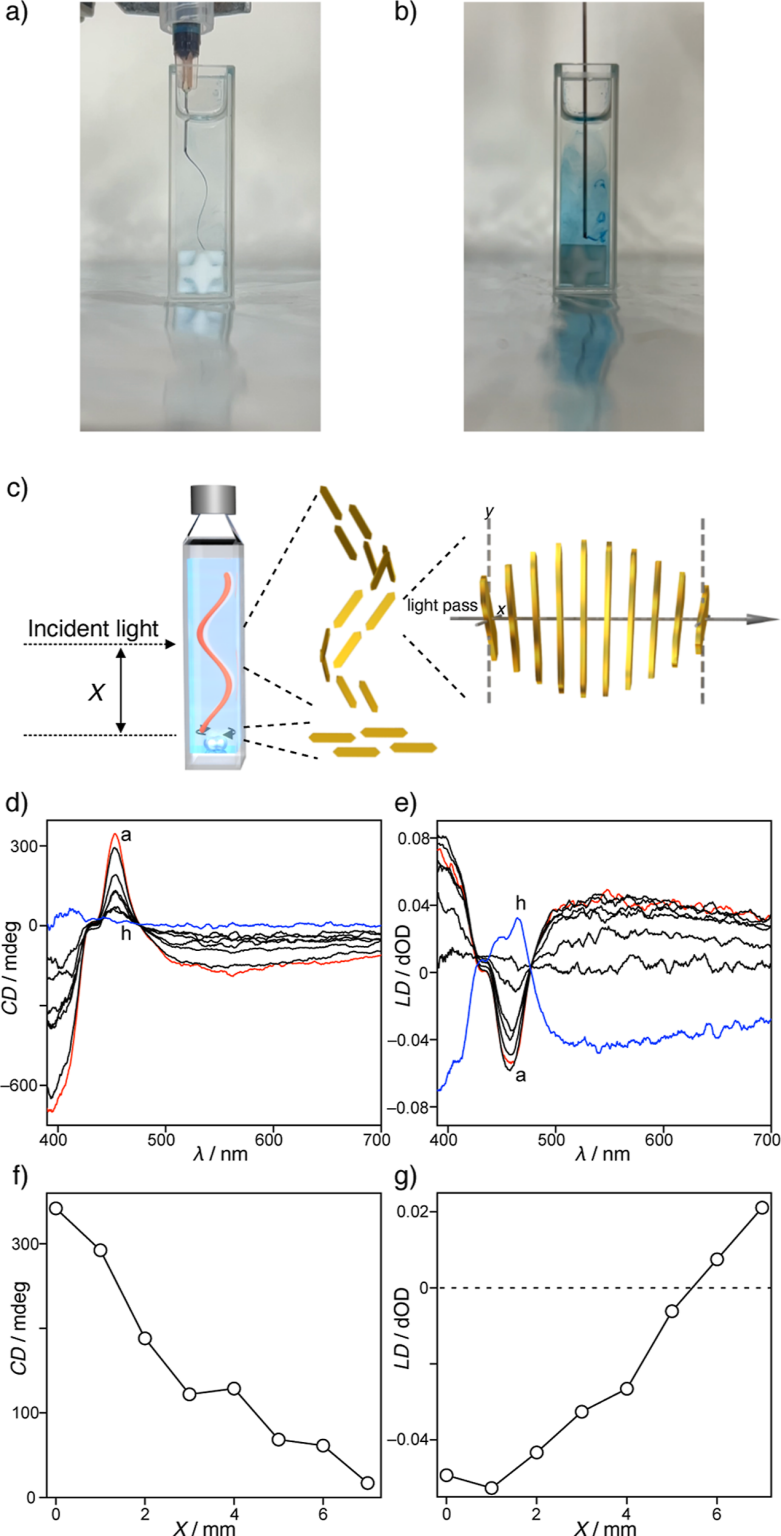
Photographic images of the ink trails in the flow generated by stirring CW at 600 rpm, in the (a) middle and (b) bottom of a 1 cm sample cuvette. (c) Incident light position during the CD measurement and schematic illustrations of the helically and horizontally ordered nanostructures, and helically displaced nanostructures along the optical path. (d,e) CD and LD spectra of the solution of **1** (3.0 × 10^–4^ M) under stirring CW at 1000 rpm and upon shifting the position of the incident light from (a) the center (*X* = 0 mm) to (h) the bottom (*X* = 7 mm) by pulling up the cuvette in 1 mm steps. (f,g) Plots of the CD and LD intensities at 452 nm versus the incident light position (*X*) from panel (c).

Finally, the CD and LD spectra recorded for a solution of **1** were measured by shifting the position (*X*) of the incident light from the center of the sample cuvette to the bottom (Figure 8d,e). It was found that the trisignate CD signals gradually weakened and disappeared upon reaching the bottom of the cuvette. In addition, intense CD signals were maintained using a margin-masked cuvette, but were considerably weakened in a center-masked cuvette (Figure S32). These observations clearly indicate that the chiral macroscopic ordering of the nanostructures exists only at the center of the sample cuvette, while the nanostructures are randomly oriented near the bottom and wall. In contrast, the intense LD signal detected at the center of the sample cuvette was gradually inverted by shifting the incident light to the bottom area of the cuvette. In general, the sign of an LD signal indicates the vertical and horizontal orientation orders of an anisotropic nanostructure. Thus, the observed inversion of the LD signal can be accounted for by considering that the vertical excess of orientation at the center of the cuvette is transformed into a horizontal excess at the bottom. The plots of the CD and LD intensities versus the position (*X*) of the incident light show good linear correlations (Figure 8f,g), demonstrating that the chiral macroscopic order is established solely by the vortex flow-oriented nanostructures at the center of the sample cuvette, whereas these nanostructures are dominantly reoriented to the achiral horizontal orientation at the bottom. Considering that Ribó et al. reported that the helical arrangement of anisotropic nanotubes simulates a CD signal,^59^ it was concluded that vortex flow-directed CD induction observed herein was produced by the helical macroscopic order generated by the vortex flow-oriented anisotropic platelet nanostructures, which act as macroscopic polarizers. These polarizers are helically displaced along the optical path from the front to the back of the cuvette (Figure 8c), resulting in selective polarization of the absorbed light and thereby generating the observed CD and LD signals.

## Conclusion

In conclusion, this study elucidated the mechanistic intricacies of circular dichroism (CD) and linear dichroism (LD) that were induced by stirring a solution containing an achiral platinum complex. The cooperative supramolecular assembly of the achiral platinum complex produced anisotropic platelet supramolecular nanostructures that were oriented within the flow, thereby generating a flow-oriented macroscopic order in solution. The direction of rotation in the vortex flow was found to drive right- and left-handed helical macroscopic orders, resulting in intense CD and LD effects. The results presented herein can be generalized to the formation of chiral macroscopic orders in solutions containing anisotropic nanostructures.

## Supplementary Material



















## Notes

Experimental and analytical procedures, UV–vis spectra, CD spectra, DLS spectra, CPL spectra, AFM images, TEM images, CLSM images, and supporting movies (PDF)
Some colored lines are tracking lines of the trajectory of particles made by complex **1** (AVI)
Some colored lines are tracking lines of the trajectory of particles made by complex **1** (AVI)
Some colored lines are tracking lines of the trajectory of particles made by complex **1** (AVI)
Some colored lines are tracking lines of the trajectory of particles made by complex **1** (AVI)
The flow velocities of blue ink trails injected by a syringe pump at stirring rates of 300 rpm (MOV)
The flow velocities of blue ink trails injected by a syringe pump at stirring rates of 600 rpm (MOV)
The flow velocities of blue ink trails injected by a syringe pump at stirring rates of 800 rpm (MOV)
The flow velocities of blue ink trails injected by a syringe pump at stirring rates of 1000 rpm (MOV)
