# Supplementary material for: Vortex-Flow-Directed Chiral Macroscopic Ordering of Platelet Nanostructures Formed via the Supramolecular Assembly of Platinum Complexes with Bis(phenylisoxazolyl)benzene
Source: J Am Chem Soc. 2025 Aug 13;147(34):30674–83. doi: 10.1021/jacs.5c03761 (PMC12395485; doi:10.1021/jacs.5c03761)
Supplement: Supplementary file 1 [file ja5c03761_si_001.pdf]

Supporting Information For:

Vortex-Flow-Directed Chiral Macroscopic Ordering of Platelet Nanostructures Formed via the Supramolecular Assembly of Platinum Complexes with Bis(phenylisoxazolyl)benzene

Masaya Yoshida,[‡,§] Kyota Yasuda,[‡,¶,‖] Takuma Matsumoto,[#] Yudai Ono,[‡] Naoyuki Hisano,[§] Mao Kawasaki,[‡,§] Takehiro Hirao,[§] Ye Yuan,[‡] Shin-ichi Tate,[‡,¶,‖] Martin Vacha[#] and Takeharu Haino*[‡,§]

[§]Department of Chemistry, Graduate School of Advanced Science and Engineering, Hiroshima University, 1-3-1 Kagamiyama, Higashi-Hiroshima, Hiroshima 739-8526, JAPAN

[‡]International Institute for Sustainability with Knotted Chiral Meta Matter (SKCM^2), Hiroshima University, 1-3-1 Kagamiyama, Higashi-Hiroshima, Hiroshima 739-8526, JAPAN

[¶]Program of Mathematical and Life Sciences, Graduate School of Integrated Sciences for Life, Hiroshima University 1-3-1 Kagamiyama, Higashi-Hiroshima, Hiroshima 739-8526, JAPAN

[‖]Research Center for the Mathematics on Chromatin Live Dynamics, Hiroshima University, 1-3-1 Kagamiyama, Higashi-Hiroshima, Hiroshima 739-8526, JAPAN

[#]Department of Materials Science and Engineering, Institute of Science Tokyo Ookayama 2-12-1-S8-44, Meguro-ku, Tokyo 152-8552, JAPAN

To whom correspondence should be addressed: *E-mail: haino@hiroshima-u.ac.jp

Contents

1. **General Information** S3-S5
2. **Experimental Procedures and Characterization Data** S6-S7
3. ***T*-Dependent and Van't Hoff plots of Nanostructure Solutions** S8
4. **Curve Fitting Data of Self-Assembly of 1** S9
5. **Dependence of CD intensity of Nanostructure Solutions on Stirring Speed** S10
6. **UV- vis absorption spectra of Nanostructure Solutions with Stirring at Various Speeds** S11
7. **Temperature-dependent CD spectra of Nanostructure Solutions** S11
8. **CPL spectra of Nanostructure Solutions** S12
9. **AFM Data of Nanostructures** S13-S15
10. **TEM Images of Nanostructures** S16-S17
11. **Temperature-dependent DLS spectra of Nanostructure Solutions** S18
12. **Absorption Polarization Anisotropy of Control Sample** S19
13. **Temperature-Dependent Emission Spectra of Complex 1** S19
14. **Microscopic Images of Emission from Nanostructures** S20-S24
15. **Plots and Fitting Curves of Emission Intensity Changes** S25-S28
16. **PXRD Patterns of Solid 1** S29
17. **Photographs and Crystal Structure of 2.** S29-S31
18. **TD-DFT Calculation of Dimer 2•2** S32-S36
19. **Time-Lapse CLSM Experiments of Complex 1..** S37-S44
20. **Picture of Center-Masked and Margin-Masked Sample Cuvettes, and CD and LD spectra of 1** S45
21. **Flow Velocities of Blue Ink Trails** S46
22. **TD-DFT Calculation of Monomer 2** S47-S49
23. **^{1}H and ^{13}C NMR, and Mass spectra** S50-S53
24. **Reference** S54

1. General Information

All solvents were commercial reagent grade and were used without further purification. Toluene for UV-vis, Fluorescence (FL), circular dichroism (CD), and confocal laser scanning microscope measurement was a Specially Prepared Reagent for HPLC (NACALAI TESQUE, INC., Kyoto, Japan). UV-vis absorption spectra were recorded on a JASCO V-760 spectrometer. FL spectra were measured using a JASCO FP-6500 spectrometer. CD spectra were recorded on a JASCO J-1500 spectrometer.

Atomic Force Microscopy: AFM measurements were carried out by using Agilent 5100 microscope in air at ambient temperature with a silicon cantilever (NCHV, Bruker, Germany) in the tapping mode in a scan rate of 1 Hz. The cantilever with a resonace frequency of 320 kHz was employed using a silicon probe tip with a nominal spring constant of 42 N/m and a radius of 8 nm. The vertical noise in the AFM instrument was <0.02 nm. AFM micrographs were recorded with a resolution of 1024 × 1024. The WSxM software[60] was used for the AFM image analysis.

Absorption anisotropy measurement on individual nanostructures using fluorescence microscopy: The absorption anisotropy (linear dichroism) cannot be measured directly on individual nanostructures because the change of transmitted intensity due to absorption of a single nanostructure is too small for reliable analysis. Instead, we assume that the amount of absorbed light is directly proportional to the intensity of fluorescence that the nanostructure subsequently emits, and that the proportionality is same for all polarization directions of the incident light. The advantage of monitoring absorption *via* the detected fluorescence lies in the fact that fluorescence microscopy is virtually zero-background technique which enables reliable detection of very weak signal even from single molecules of small organic dyes. Further, while the spatial resolution in optical microscopy is determined by the diffraction limit, i.e., 200 ~ 300 nm, the use of fluorescence microscopy enables the study of fluorescence-monitored absorption of objects with sizes below the spatial resolution, and even of organic dyes smaller than 1 nm, as mentioned above. The only requirement for such measurement is that the nano-object (or single molecule) efficiently absorbs and emits light, and that there are no other light absorbing and emitting objects around (within distances smaller than the diffraction limit). Here, we use wide-field fluorescence microscopy to study the absorption anisotropy of the nanostructures at a fixed excitation wavelength of 442 nm. The output of the excitation laser (continuous wave He-Cd laser, Kimmon, Tokyo, Japan, intensity of 1.74 W/cm^2) is first circularly polarized using a quarter-wave plate (Berek compensator 5540, New Focus), and the polarization state is verified above the objective lens (Uplan FLN 100×/1.3 NA, Oil, Olympus, Tokyo, Japan) at the microscope sample stage of an inverted optical microscope (IX71, Olympus, Tokyo, Japan). At this point, the Berek compensator is used to pre-compensate for the polarization distortion by the optical elements within the microscope and to achieve high degree of circular polarization at the sample stage within +- 1%. Further, a linear polarizer is inserted into the laser path to select a linear polarization component of the excitation laser, the direction of which is determined by the transmission axis of the polarizer. This ensures that the incident light within the microscopic wide-field image (approx. 20 μm in diameter) is linearly polarized with the same direction at each sample location. The individual nanostructures within the microscopic field of view are therefore excited with the same linearly polarized laser light. Next, the linear polarizer is continuously rotated with a motor with a speed of 1°/100 ms, and wide-field fluorescence images are

simultaneously and continuously detected using an electron-multiplying (EM) charge-coupled device (CCD) camera (iXon, Andor Technology, Belfast, UK) with an exposure time of 500 ms. The result is a series of microscopic images taken with steps of 5°, each corresponding to fluorescence excited with specific linear polarization. The fluorescence intensity of individual nanostructures is then analyzed from each image in the series, and plotted as a function of the linear polarizer angle, as shown in the example in Fig. 5c. Since the absorption is theoretically proportional to the square of the dot product of the vectors of electric field and transition dipole moment ($|\mathbf{E} \cdot \boldsymbol{\mu}|^2$), for rotating linear polarization (rotating **E**) the fluorescence intensity is fitted with a $\cos^2$ function of the linear polarizer angle. As mentioned above, here we assume the direct proportionality between absorption and fluorescence (by the PLQY). The degree of absorption anisotropy, defined as $D_{ex} = (I_{max} - I_{min})/(I_{max} + I_{min})$, where I_{max} and I_{min} are the maximum and minimum fluorescence intensities, is further analyzed from the intensity plots for individual nanostructures and summarized in the histogram in Fig. 5d. In the fluorescence image in Fig. 5b the individual nanostructures are oriented randomly within the sample plane and this is reflected in different phases of the $\cos^2$ modulated fluorescence intensities (Fig. 5c) of each nanostructure. However, because of the high degree of the circular polarization of the laser, the nanostructure orientation does not affect its D_{ex} value.

Confocal laser scanning microscopy: Microscopic images were collected using an inverted laser scanning microscope (IX83, Olympus) equipped with a spinning disk (CSU-W1, YOKOGAWA, Tokyo, Japan). Samples were dropped onto a cover glass (25 mm, Deckgläser, Germany) set in a cell chamber (TOKAI HIT, Shizuoka, Japan) and observed. Images were collected using a 100x objective oil lens (NA: 1.4, UPLSAPO 100XO, Olympus, Tokyo, Japan). The stocked solution of complex **1** was excited with a 488 nm laser (OBIS, Coherent Inc., Santa Clara, CA, USA). Time-lapse photography was carried out in single-plane mode for 24 seconds at 200 ms intervals.

Computational Methods: Time-dependent density functional theory (TD-DFT) calculations were performed using Gaussian 16 Rev. C01 program.[61] The initial geometries of monomer **2** and dimer **2•2** were obtained from the crystal structures and used for the TD-DFT calculations without geometry optimization. The UV/vis absorption spectra and transition dipole moments were estimated by the TD-DFT at the M06/6-31g** + LanL2DZ level of theory. The 6-31g** basis set was applied for the carbon, hydrogen, oxygen, and nitrogen atoms and the LanL2DZ basis set was applied for the platinum atom. The GaussView 6 program was used to visualize the molecular structures.

Powder X-ray diffraction: PXRD data were collected on a Rigaku SmartLab diffractometer using Cu*K*α radiation (λ = 1.54184 Å).

Single-crystal X-ray diffraction analysis: X-ray crystallographic data were obtained at 173 K on a Rigaku XtaLAB Synergy R DW system equipped with a HyPix diffractometer using Mo*K*α radiation (λ = 0.71073 Å). Using Olex2,[62] the structure was solved with the SHELXD[63] structure solution program using Dual Space or SHELXT[64] structure solution program using Intrinsic Phasing and refined with the SHELXL[65] refinement package using Least Squares minimization.

Visualization of stirred vortices: Video images were captured using a smartphone (iPhone 14, Apple, USA) with the slow-shot function (240 fps). The vortex flow by stirring in a 1 cm sample cuvette was visualized by continuously injecting the blue ink for fountain pens (SEIBOKU, SAILOR, Hiroshima, Japan) into ethanol using a syringe pump (YSP-101, YMC, Kyoto, Japan) (10 µL / min). The acquired video was converted to a 30 fps image stack after audio was removed using ffmpeg. The images were processed using Fiji (ImageJ2, version 2.16.0/1.45p) with a manual tracking plugin to estimate the flow speed.[66]

Analysis of cooperative self-assembly by temperature-dependent UV-vis absorption spectra:
The degree of aggregation (α_{agg}) was calculated from equation 1:

$$\alpha_{agg}(T) = \frac{\varepsilon(T) - \varepsilon_m}{\varepsilon_a - \varepsilon_m} \qquad (1)$$

where $\varepsilon(T)$ is the measured extinction coefficient at the absolute temperature T and ε_m, and ε_a are the extinction coefficients of the monomer and fully aggregated state, respectively. The latter two values could be determined from the data or by extrapolating the measured extinction coefficient to their asymptotic value at high and low temperatures. Non-sigmoidal melting curves were obtained by plotting $\alpha_{agg}(T)$ vs T. The curve-fitting analysis of the plots was carried out based on the van der Schoot mathematical model.[30,31]
In the elongation regime, $\alpha_{agg}(T)$ is given by equation 2:

$$\alpha_{agg}(T) = \alpha_{sat}\left(1 - exp\left[\frac{-\Delta H_e}{RT_e^2}(T - T_e)\right]\right) \qquad (2)$$

where ΔH_e is the molecular enthalpy release due to noncovalent interactions during elongation, T_e is the elongation temperature, and R is the gas constant. α_{sat} is introduced as a parameter to ensure that αagg(T)/α_{sat} does not exceed unity. At temperatures above the elongation temperature T_e (i.e., the nucleation regime), the fraction of aggregated molecules is described by equation 3:

$$\alpha_{agg}(T) = K_a^{\frac{1}{3}} exp\left[\left(\frac{2}{3}K_a^{\frac{1}{3}} - 1\right)\frac{h_e}{RT_e^2}(T - T_e)\right] \qquad (3)$$

where K_a is the dimensionless equilibrium constant of the activation step at T_e. The average length of the stack, $\langle N_n(T_e)\rangle$, averaged over the nucleated species at the elongation temperature, is given by equation 4:

$$\langle N_n(T_e)\rangle = \frac{1}{K_a^{\frac{1}{3}}} \qquad (4)$$

Hence, a higher degree of cooperativity is expressed in a smaller Ka value and will lead to a larger nucleus before elongation sets in.

2. Experimental Procedures and Characterization Data

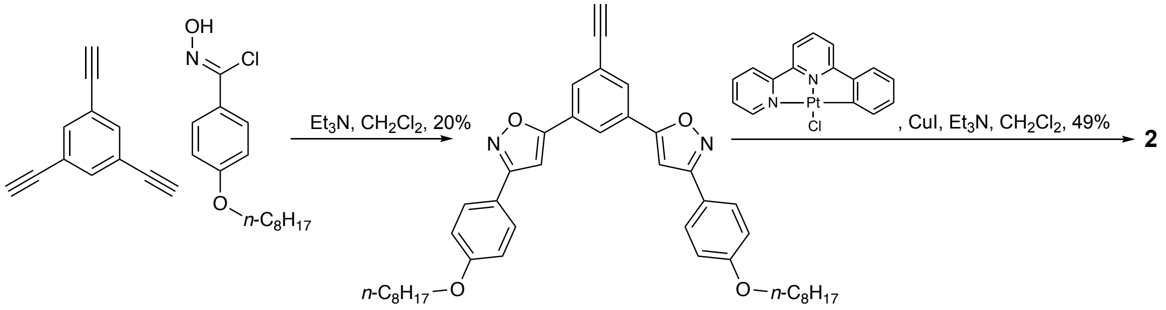

Scheme S1. Synthesis of compound **2**.

5,5'-(5-ethynyl-1,3-phenylene)bis(3-(4-(octyloxy)phenyl)isoxazole)

To a mixture of *N*-hydroxy-4-(octyloxy)benzimidoyl chloride (0.57 g, 2.0 mmol) and 1,3,5-triethynyl benzene (0.15 g, 1.0 mmol) in dry dichloromethane (23 mL) was added dry triethylamine (1.2 mL, 7.0 mmol). After being stirred at room temperature for 48 h under an argon atmosphere, the resulting mixture was poured into water and extracted with methylene chloride. The organic layer was washed with saturated aqueous sodium chloride, dried over anhydrous sodium sulfate, and concentrated in *vacuo*. The crude product was purified by column chromatography on silica gel (10% ethyl acetate in *n*-hexane, eluent) to give the desired compound (127 mg, 20 %) as white solid: ^{1}H NMR (400 MHz, $CDCl_3$): δ 8.25 (t, 1H, J = 1.7 Hz), 8.01 (d, 2H, J = 1.7 Hz), 7.81 (AA'BB', 4H), 7.00 (AA'BB', 4H), 6.91 (s, 2H), 4.02 (t, 4H, J = 6.7), 3.24 (s, 1H), 1.82 (quint, 4H, J = 6.6 Hz), 1.50 (m, 4H), 1.20–1.40 (m, 16H), 0.89 (t, 6H, J = 6.7 Hz) ppm; ^{13}C NMR (101 MHz, chloroform-d_1) δ 168.2, 162.9, 160.9, 130.4, 128.8, 128.3, 124.2, 123.0, 121.0, 115.0, 98.7, 81.9, 79.5, 68.3, 32.0, 29.5, 29.4, 29.3, 26.2, 22.8, 14.3 ppm; HRMS (ESI^-) calcd for $C_{42}H_{49}O_4N_2$ *m*/*z* 645.36868 $[M + Cl]^-$, found *m*/*z* 645.36859.

6-(Phenyl-2,2'-bipyridine){5,5'-(5-Ethynyl-1,3-phenylene)bis-(3-(4-octyloxy)isoxazole)}platinum (2)

To a solution of 5,5'-(5-ethynyl-1,3-phenylene)bis(3-(4-(octyloxy)phenyl)isoxazole) (127 mg, 0.20 mmol) and (6-phenyl-2,2'-bipyridine)platinum chloride (91 mg, 0.20 mmol) in dry dichloromethane (15 mL) and dry dimethylformamide (9 mL) was added dry triethyl amine (220 µL, 1.4 mmol). The resulting solution was deoxygenated by bubbling nitrogen for 30 min, and CuI (8 mg, 4 µmol) was added. After being stirred at room temperature for 21 h under an argon atmosphere in the dark, the resulting reaction mixture was poured into water. The aqueous layer was extracted with dichloromethane. The organic layer was washed with saturated aqueous sodium chloride, dried over anhydrous sodium sulfate and concentrated in *vacuo*. Column chromatography on silica gel (5% methanol in CH_2Cl_2, eluent) gave the desired product **2** (105 mg, 49%) as an orange solid: ^{1}H NMR (500 MHz, chloroform-d_1) δ 9.23 (ddd, 1H, J = 5.3, 1.5, 0.7 Hz), 8.20 (t, 1H, J = 8.0 Hz), 8.07 (t, 1H, J = 1.58 Hz), 8.05 (d, 2H, J = 1.58 Hz), 8.03 (dt, 1H, J = 7.8, 1.5 Hz), 7.97 (dd, 1H, J = 7.5, 1.2 Hz), 7.89 (dt, 1H, J = 7.8, 0.7 Hz), 7.81 (AA'BB', 4H), 7.61 (dd, 1H, J = 7.8, 1.5 Hz), 7.59 (d, 1H, J = 8.0 Hz), 7.55 (d, 1H, J = 8.0 Hz), 7.36 (dd, 1H,

J = 7.5, 1.2 Hz), 7.22 (dt, 1H, *J* = 7.5, 1.2 Hz), 7.08 (dt, 1H, *J* = 7.5, 1.2 Hz), 6.99 (AA'BB', 4H), 6.88 (s, 2H), 4.02 (t, 4H, *J* = 6.6 Hz), 1.81 (quint, 4H, *J* = 6.6 Hz), 1.48 (m, 4H), 1.52–1.27 (m, 16H), 0.89 (t, 6H, *J* = 7.0 Hz) ppm; ^{13}C NMR (125.7 MHz, chloroform-d_1) δ 169.6, 165.9, 162.9, 160.8, 158.2, 154.7, 152.0, 146.9, 142.0, 139.0, 139.0, 138.6, 131.9, 130.7, 130.5, 128.4, 128.2, 127.9, 124.7, 124.1, 122.7, 121.5, 119.5, 118.7, 117.7, 115.0, 109.7, 105.0, 98.1, 68.3, 32.0, 29.5, 29.4, 29.4, 26.2, 22.8, 14.3 ppm; HRMS (ESI$^-$) calcd for $C_{58}H_{58}O_4N_4ClPt$ *m*/*z* 1104.3800 [M + Cl]$^-$, found *m*/*z* 1104.3770.

3. *T*-Dependent and Van't Hoff plots of Nanostructure Solutions

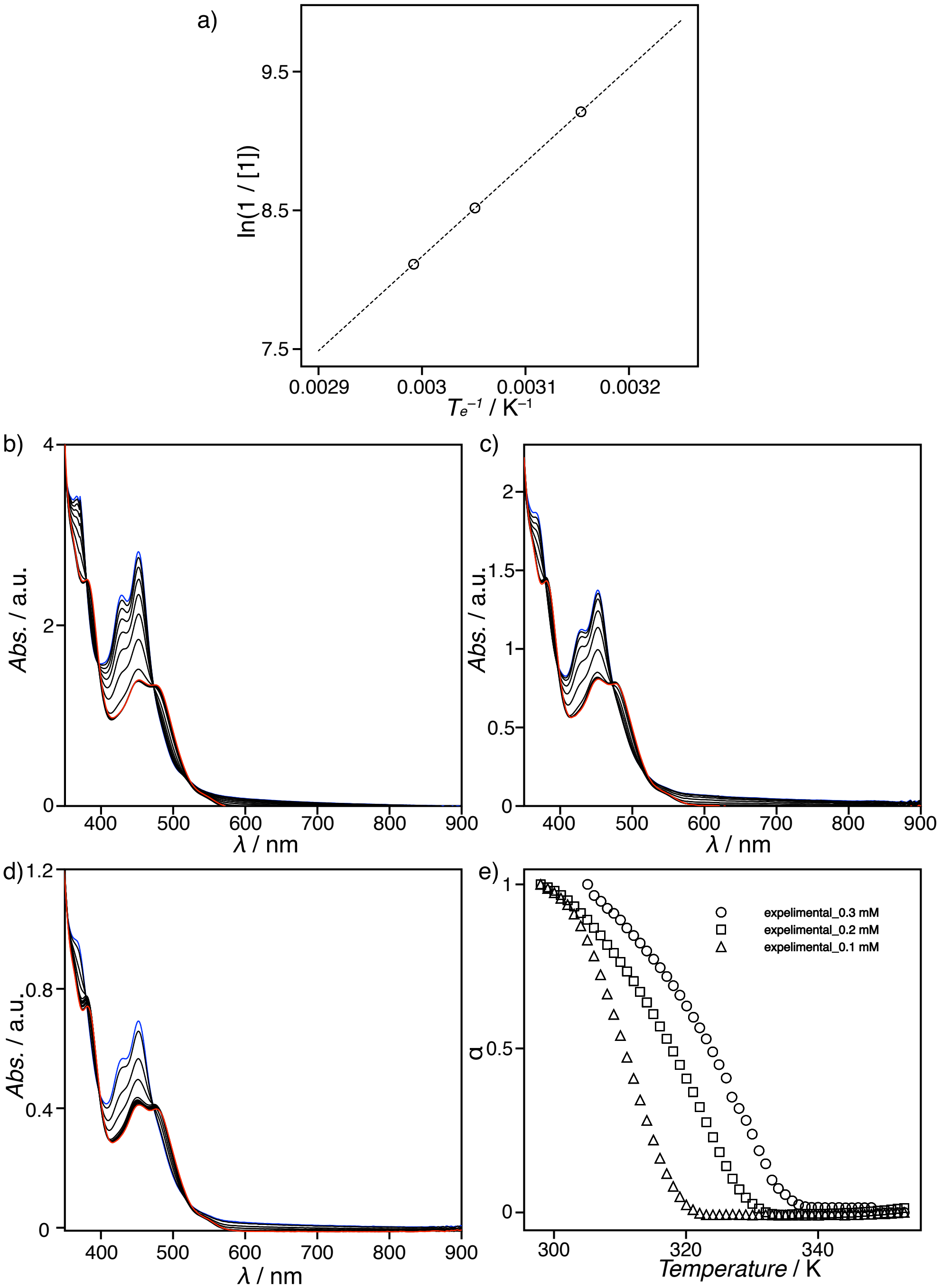

Figure S1. (a) Van't Hoff plot of **1**. (b-d) Temperature-dependent UV-vis absorption spectra of **1** at the concentration of (b) 3.0×10^{-4} M, (c) 2.0×10^{-4} M, and (d) 1.0×10^{-4} M in toluene. Temperatures are 298, 303, 308, 313, 318, 323, 328, 333, 338, 343, 348, and 353 K. The blue and red lines represent the temperature of 25 °C and 80 °C, respectively. (e) Heating plots of a degree of aggregation (b)-(d) at 420 nm of **1**.

4. Curve Fitting Data of Self-Assembly of 1

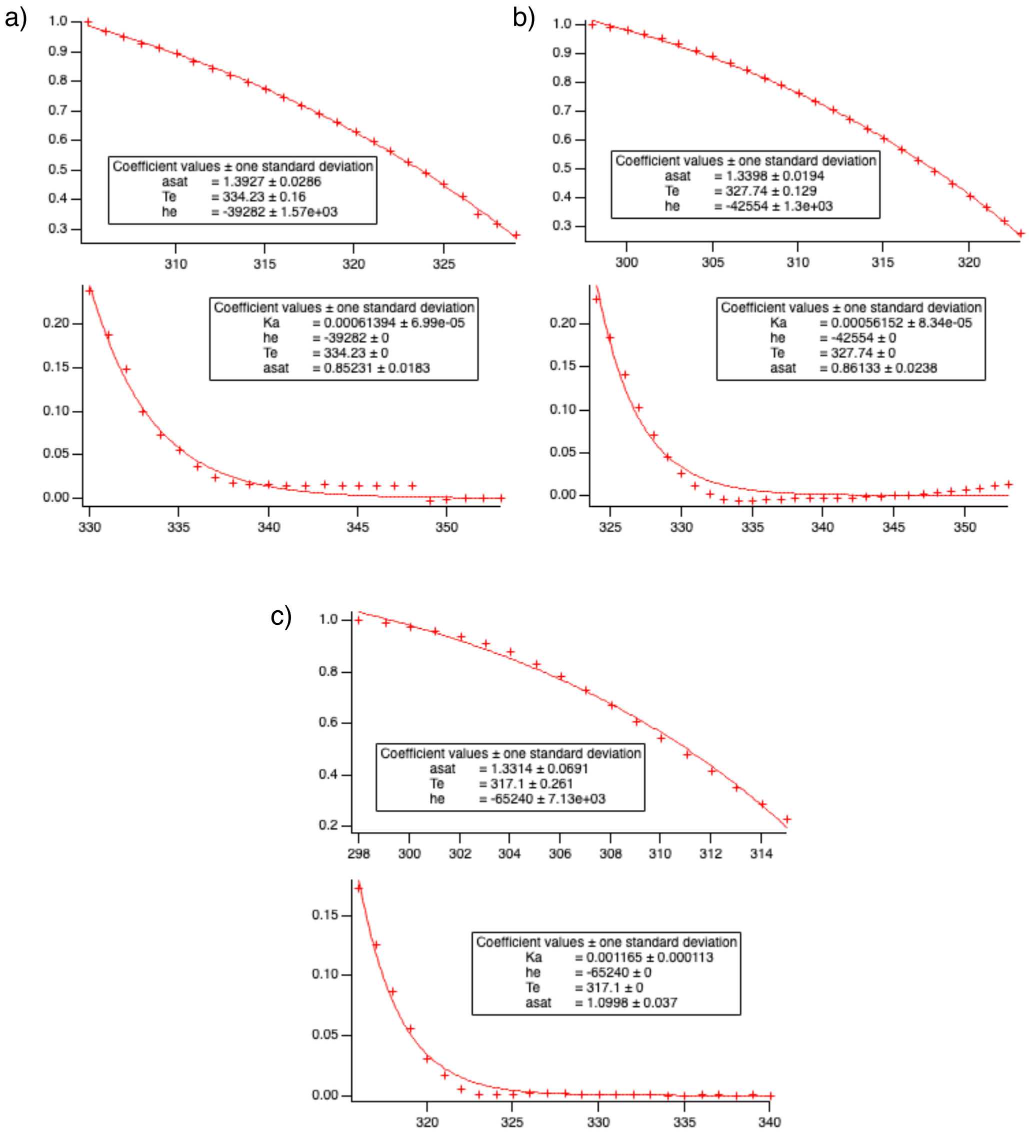

Figure S2. Fitting curves and elongation temperatures (T_e) of **1** at the concentration of (a) 3.0×10^{-4} M, (b) 2.0×10^{-4} M, and (c) 1.0×10^{-4} M in toluene.

5. Dependence of CD Intensities of Nanostructure Solutions on Stirring Speed

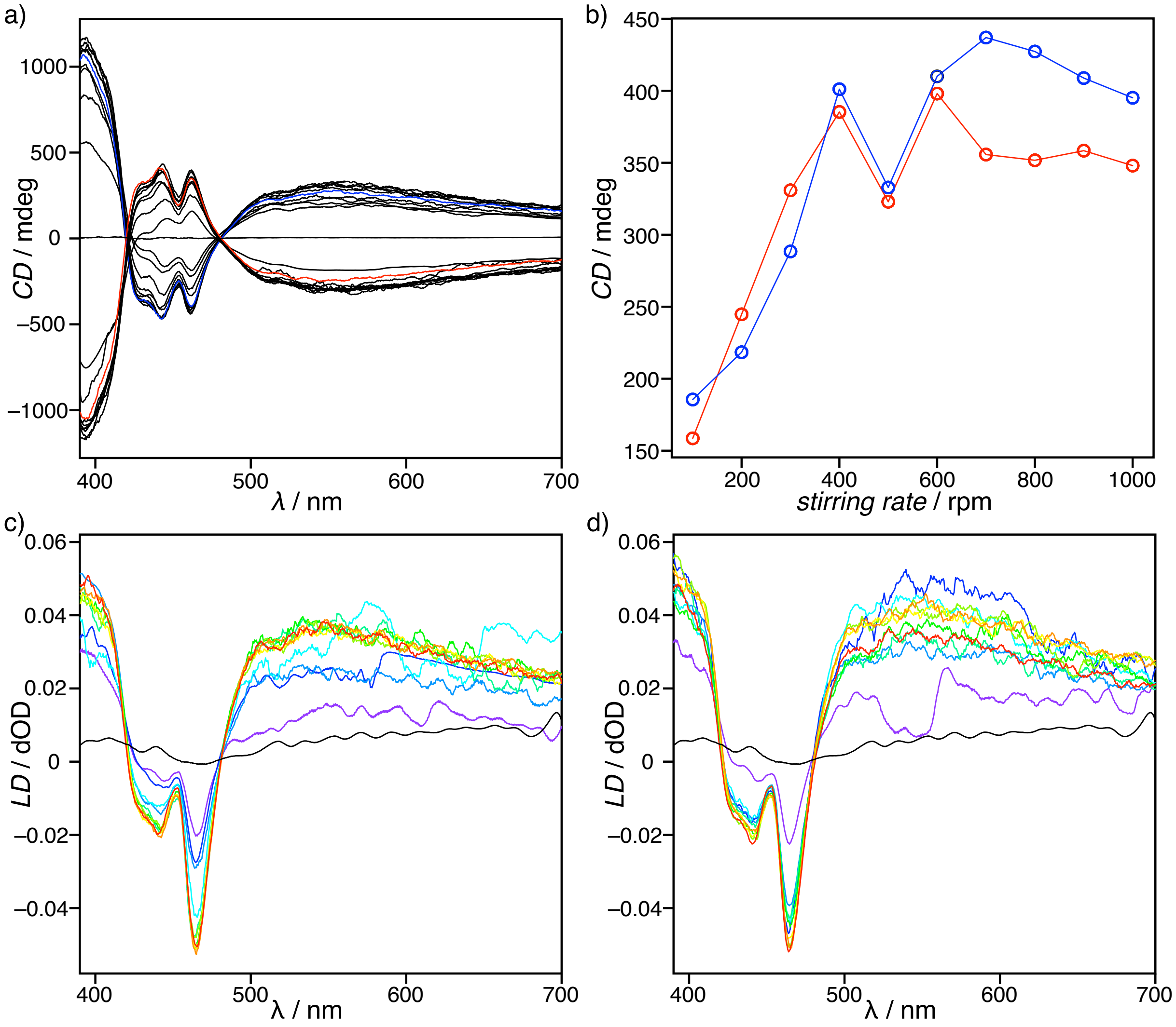

Figure S3. (a) CD spectra of **1** (3.0×10^{-4} M) in toluene at 298 K with stirring at 0, 100, 200, 300, 400, 500, 600, 700, 800, 900, and 1000 rpm. The red and blue lines indicate the 1000 rpm clockwise and counterclockwise rotation, respectively. (b) Plots of absolute CD intensity versus stirring rate at 444 nm of a toluene solution of assembly **1** (3.0×10^{-4} M) at 298 K. (c, d) LD spectra under the (c) clockwise and (d) counterclockwise rotation conditions of complex **1** (3.0×10^{-4} M) at 298 K. The color of the lines shows the stirring rate: 100 (purple), 200 (blue), 300 (light blue), 400 (pale blue), 500 (green), 600 (light green), 700 (yellow-green), 800 (yellow), 900 (orange), and 1000 (red) rpm.

6. UV- vis absorption spectra of Nanostructure Solutions with Stirring at Various Speeds

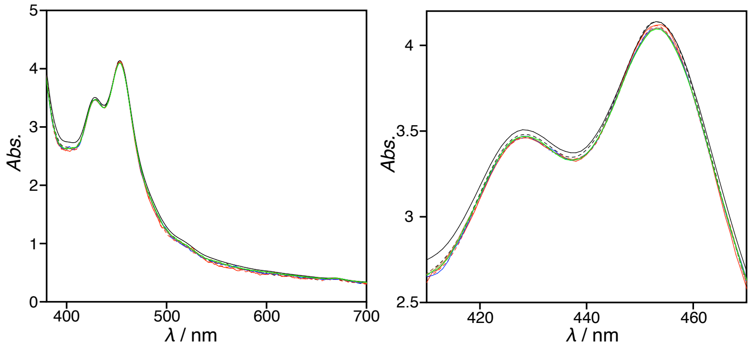

Figure S4. UV-vis absorption spectra of a toluene solution of **1** (3.0×10^{-4} M) at 298 K in a quartz optical cell stirring at 0 (solid black line), 100 (dotted black line), 200 (solid red line), 300 (dotted red line), 400 (solid blue line), 500 (dotted blue line), 600 (solid brown line), 700 (dotted brown line), and 800 (solid green line) rpm in a clockwise (CW) direction using a Teflon-coated magnetic stirring bar.

7. Temperature-dependent CD spectra of Nanostructure Solutions

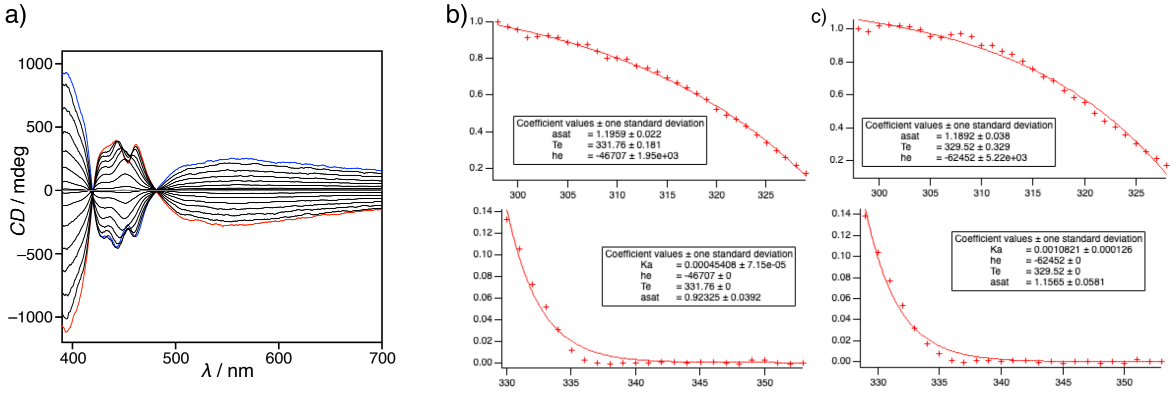

Figure S5. (a) Temperature-dependent CD spectra of a toluene solution of **1** (3.0×10^{-4} M). The temperatures were 298, 303, 308, 313, 318, 323, 328, 333, 338, 343, 348, and 353 K. The fitting curves and elongation temperature (T_e) of **1** at the concentration of 3.0×10^{-4} M in toluene with stirring in (b) clockwise and (c) counterclockwise rotation.

8. CPL Spectra of Nanostructure Solutions

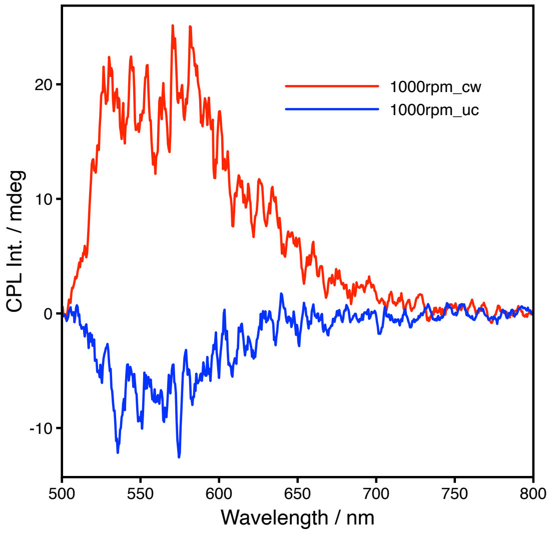

Figure S6. CPL spectra of a toluene solution of **1** (3.0×10^{-4} M) at 298 K in a quartz optical cell stirring at 1000 rpm in a clockwise (CW) direction using a Teflon-coated magnetic stirring bar (red line) and counterclockwise (CCW) (blue line).

9. AFM Data of Nanostructures

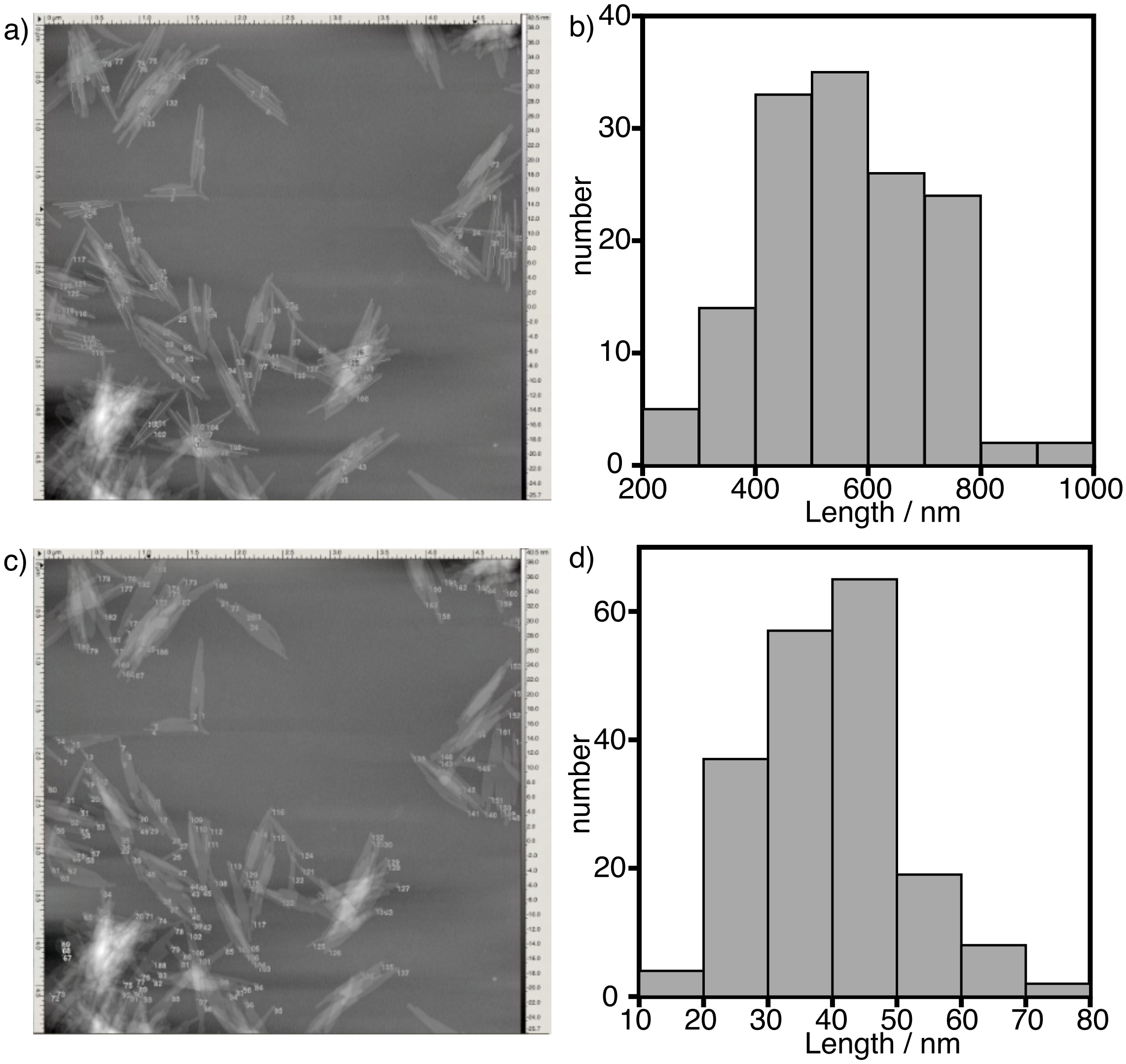

Figure S7. (a), (c) AFM image (5 µm × 5 µm) of a spin-coated sample of a toluene solution of **1** (3.0×10^{-4} M) on mica. Length distribution analyses were obtained from the supramolecular polymer of **1** at the long axis (b) and short axis (d) for calculating the number-average length (L_n), weight-average length (L_w), and PDI.

Table S1. Lengths (*L*) of the long axis of supramolecular polymers **1** measured on the image in Figure S7a.

No.	*L* (nm)	No.	*L* (nm)	No.	*L* (nm)	No.	*L* (nm)
1	552.815462	41	396.831792	81	646.378154	121	573.455095
2	556.833334	42	239.457028	82	911.923245	122	452.734417
3	431.044776	43	985.433343	83	534.996733	123	702.056072
4	736.074875	44	471.513865	84	682.197234	124	731.851992
5	726.358365	45	315.007639	85	780.349212	125	712.455721
6	592.673681	46	528.269767	86	669.586543	126	730.351893
7	854.840889	47	474.864499	87	650.129569	127	526.484036
8	497.232407	48	572.622975	88	695.436617	128	533.903786
9	496.128351	49	496.272498	89	760.089403	129	740.466884
10	469.791527	50	419.467414	90	587.56285	130	624.580242
11	369.709848	51	423.089434	91	574.638784	131	732.828663
12	460.462422	52	298.011612	92	613.85712	132	520.540791
13	759.869801	53	383.790305	93	522.803079	133	655.916402
14	285.216443	54	435.556728	94	690.205884	134	560.822422
15	390.166968	55	481.446303	95	568.695703	135	663.09241
16	556.147842	56	499.02726	96	500.744266	136	580.459418
17	455.543117	57	725.882263	97	576.502818	137	577.680262
18	601.300071	58	550.004994	98	573.808381	138	470.298752
19	538.372981	59	200.433357	99	733.869013	139	638.75325
20	528.78853	60	478.739783	100	675.806651	140	735.896705
21	595.703125	61	508.023732	101	740.933614	141	582.284348
22	752.206735	62	484.851234	102	433.251598		
23	732.486976	63	335.069111	103	501.220168		
24	490.716583	64	711.551611	104	585.713662		
25	364.383461	65	296.085311	105	735.896705		
26	404.301593	66	659.468976	106	671.204698		
27	475.667144	67	394.572341	107	537.242526		
28	634.03278	68	474.462667	108	635.89143		
29	580.315641	69	668.214273	109	671.204698		
30	794.39923	70	591.84844	110	549.831573		
31	694.06393	71	422.52554	111	726.752144		
32	665.729898	72	608.081717	112	394.421251		
33	690.620277	73	752.808716	113	401.490724		
34	341.41301	74	764.982616	114	499.313837		
35	398.420745	75	681.305461	115	484.974152		
36	748.106792	76	840.780047	116	452.418335		
37	396.260617	77	589.547797	117	605.350606		
38	327.294161	78	456.431982	118	424.692424		
39	444.791791	79	609.315527	119	428.186744		
40	548.898496	80	452.022922	120	319.926704		

$L_n = 563 \pm 37$, $L_w = 600 \pm 85$, PDI = 1.06[67,68]

Table S2. Lengths (L) of the short axis of supramolecular polymers **1** measured on the image in Figure S7c.

No.	L (nm)	No.	L (nm)	No.	L (nm)	No.	L (nm)	No.	L (nm)
1	45.0173069	41	28.9054392	81	34.914808	121	42.1974621	161	51.9454806
2	29.7009889	42	23.6977211	82	37.0096225	122	63.3502693	162	42.1974621
3	29.7009889	43	41.3780159	83	25.9067358	123	49.3769951	163	26.6880183
4	22.2057735	44	28.1856888	84	41.3780159	124	49.7913547	164	45.1760015
5	33.3086603	45	28.1856888	85	40.0320275	125	26.6880183	165	40.8784642
6	33.3086603	46	23.6977211	86	45.024149	126	31.8368811	166	51.5484392
7	29.8381116	47	31.8368811	87	33.3086603	127	51.9454806	167	33.1024127
8	43.3186525	48	26.169755	88	39.3417684	128	44.565487	168	47.3954422
9	19.9302917	49	38.6391803	89	44.565487	129	41.8716081	169	57.5734611
10	28.9054392	50	39.3417684	90	29.8381116	130	40.0320275	170	39.8605833
11	26.6880183	51	41.3780159	91	23.4069405	131	40.0320275	171	37.0096225
12	16.5512064	52	34.121186	92	33.5136386	132	33.1024127	172	52.3395101
13	31.8368811	53	40.8784642	93	44.411547	133	34.121186	173	37.0096225
14	45.7783748	54	34.121186	94	43.1602657	134	31.4037061	174	39.3417684
15	41.3780159	55	31.8368811	95	31.4037061	135	45.1760015	175	47.3954422
16	56.6138362	56	48.1125093	96	48.254644	136	45.1760015	176	28.1856888
17	31.8368811	57	45.024149	97	59.215396	137	53.3760367	177	34.914808
18	37.0096225	58	61.0378331	98	29.607698	138	51.4154108	178	47.3954422
19	43.1602657	59	23.6977211	99	40.8784642	139	36.6376571	179	78.0718842
20	35.1104107	60	38.1037385	100	37.1942103	140	29.8381116	180	67.0272771
21	38.1037385	61	34.914808	101	37.7425575	141	23.6977211	181	42.1974621
22	20.935804	62	61.0378331	102	41.3780159	142	34.914808	182	26.6880183
23	28.1856888	63	52.3395101	103	38.1037385	143	45.1760015	183	44.718897
24	31.8368811	64	49.3769951	104	39.8605833	144	44.565487	184	37.0096225
25	24.8268095	65	46.813881	105	54.6440528	145	74.019245	185	39.8605833
26	38.6391803	66	37.7425575	106	31.6210353	146	66.2048254	186	50.3385289
27	31.8368811	67	26.169755	107	43.1602657	147	51.9454806	187	55.0187592
28	31.8368811	68	36.4502509	108	42.1974621	148	41.3780159	188	55.0187592
29	41.3780159	69	29.8381116	109	41.3780159	149	40.8784642	189	56.4927369
30	43.3186525	70	24.8268095	110	37.7425575	150	48.254644	190	43.3186525
31	18.5048113	71	37.0096225	111	22.2057735	151	44.411547	191	61.2618259
32	42.1974621	72	61.5962879	112	37.7425575	152	56.4927369	192	66.2048254
33	13.3440092	73	28.9054392	113	36.4502509	153	55.0187592		
34	20.935804	74	37.0096225	114	29.8381116	154	42.1974621		
35	28.1856888	75	37.1942103	115	33.3086603	155	49.7913547		
36	31.6210353	76	47.1055591	116	43.1602657	156	45.024149		
37	26.6880183	77	40.0320275	117	42.1974621	157	51.9454806		
38	23.6977211	78	48.254644	118	45.024149	158	45.024149		
39	44.718897	79	48.254644	119	31.6210353	159	40.7105848		
40	26.169755	80	39.8605833	120	35.1104107	160	48.6785583		

$L_n = 40 \pm 2$, $L_w = 43 \pm 6$, PDI = 1.08[67,68]

10. TEM Images of Nanostructures

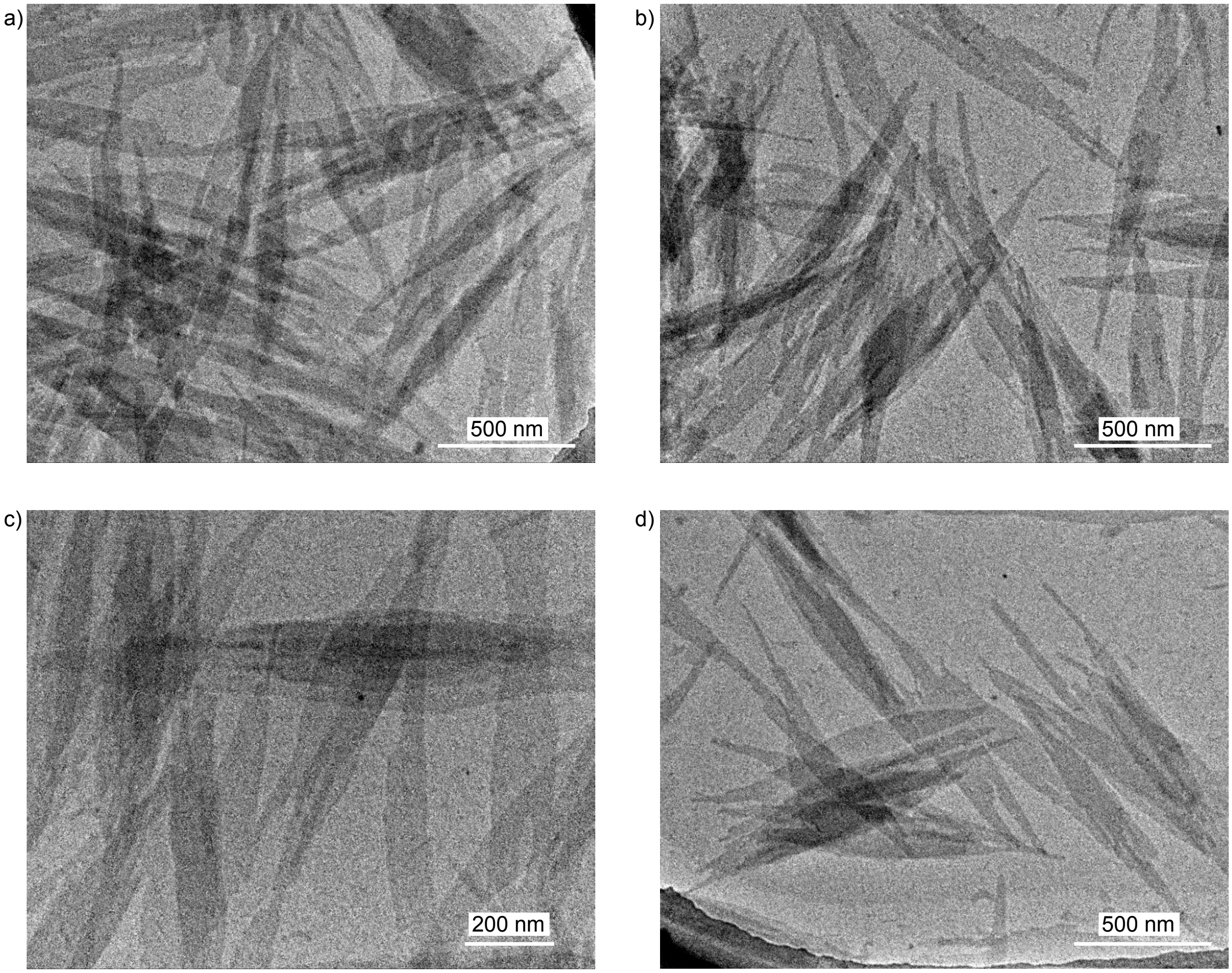

Figure S8. Transmission Electron Microscopic (TEM) images of a drop-casted sample of complex **1** at 298 K in toluene.

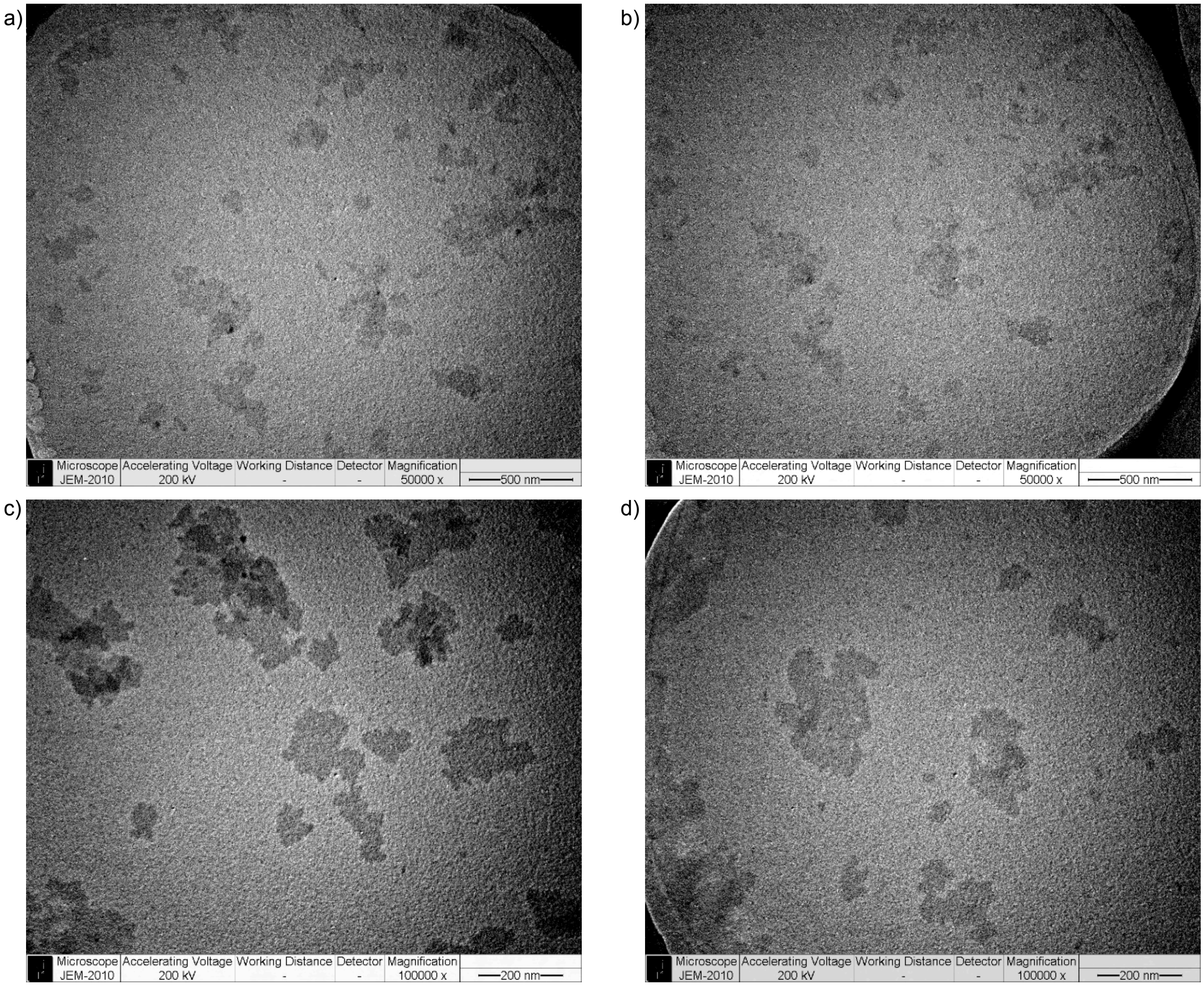

Figure S9. Transmission Electron Microscopic (TEM) images of a drop-casted sample of complex **1** at 353 K in toluene.

11. Temperature-dependent DLS spectra of Nanostructure Solutions

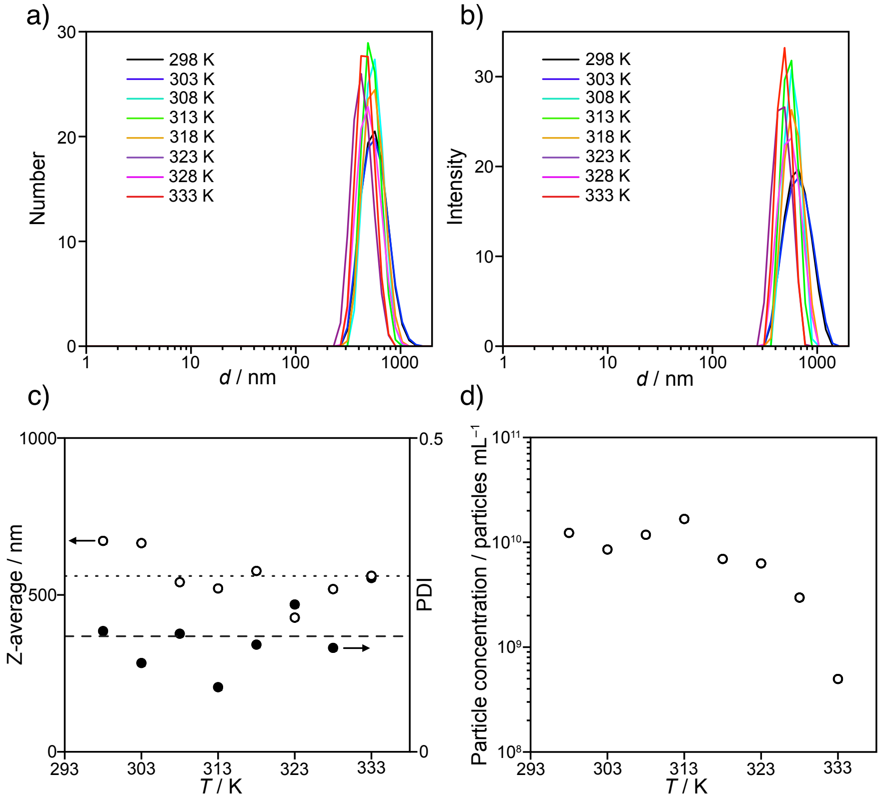

Figure S10. (a,b) Temperature-dependent DLS spectra of a toluene solution of **1** (3.0×10^{-4} M). (c) Z-averages and polydispersity indices (PDI) of a toluene solution of **1** (3.0×10^{-4} M) at various temperatures. (d) Particle concentrations of a toluene solution of **1** (3.0×10^{-4} M) at various temperatures.

12. Absorption Polarization Anisotropy of Control Sample

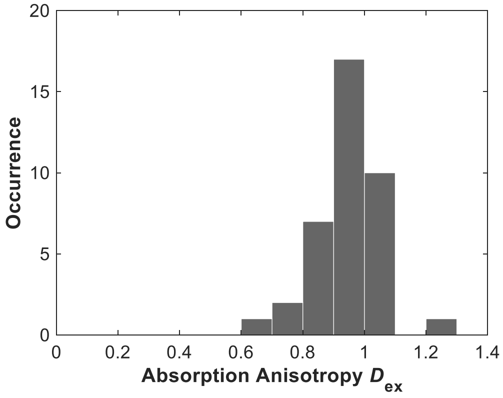

Figure S11. Results of the control experiment. Absorption polarization anisotropy was measured for a single PDI derivative.

Note: Control experiments on the absorption polarization anisotropy were carried out under the same conditions using single PDI derivative (*N*, *N*'-dioctyl-3,4,9,10-perylenedicarboximide). It is assumed that the single PDI derivative shows anisotropy due to the uniqueness of the transition dipole moment. The results (Figure S10) show that the anisotropy values are distributed around 1 value for the single PDI derivative. Thus, it was confirmed that the developed apparatus for measurement can accurately measure polarization anisotropy.

13. Temperature-dependent emission spectra of complex 1.

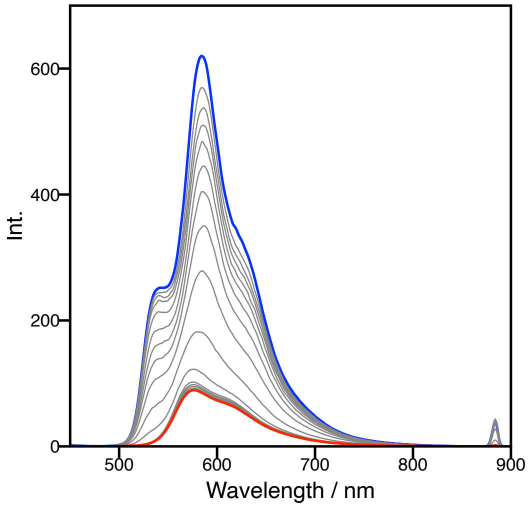

Figure S12. Temperature-dependent FL spectra change of **1** in toluene (3.0×10^{-4} M) during heating runs. The temperatures were 298, 303, 308, 313, 318, 323, 328, 333, 338, 343, 348, and 353 K. The blue and the red curves indicate the spectra at 298 K and at 353 K, respectively.

14. Microscopic Images of Emission from Nanostructures

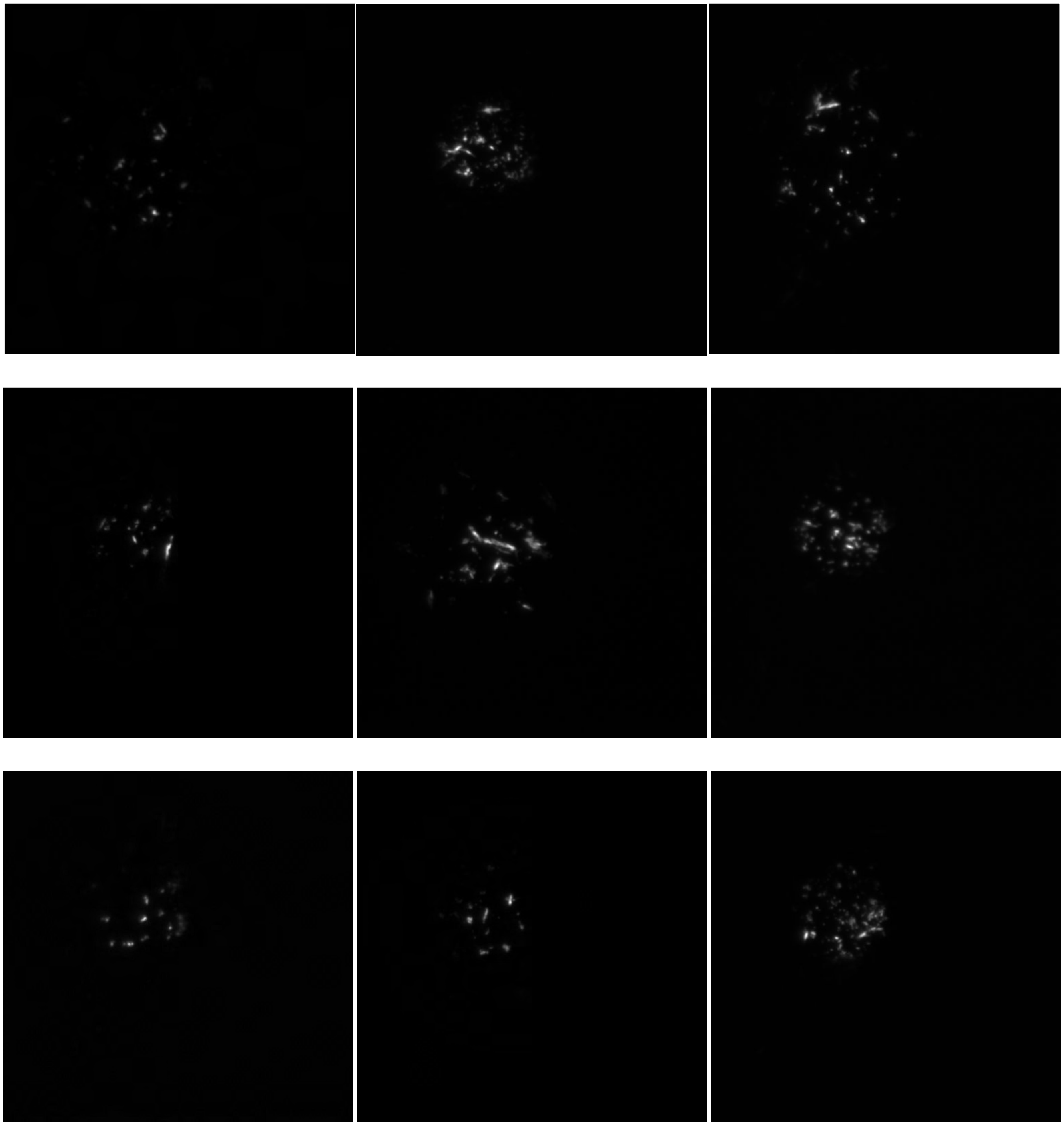

Figure S13. Microscopic images (50.5 µm × 50.5 µm) of emission from nanostructures for absorption polarization anisotropy.

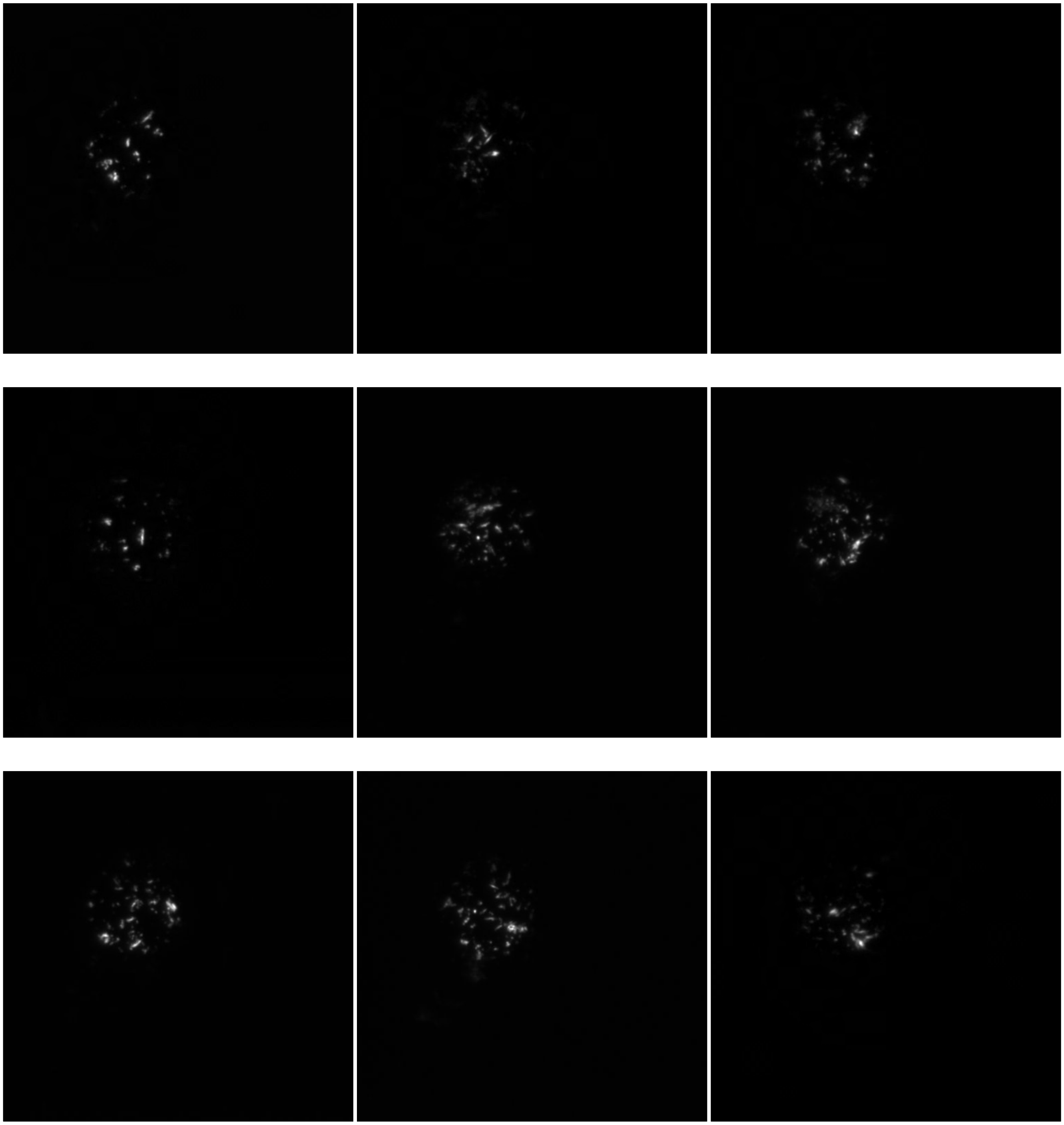

Figure S14. Microscopic images (50.5 μm × 50.5 μm) of emission from nanostructures for absorption polarization anisotropy.

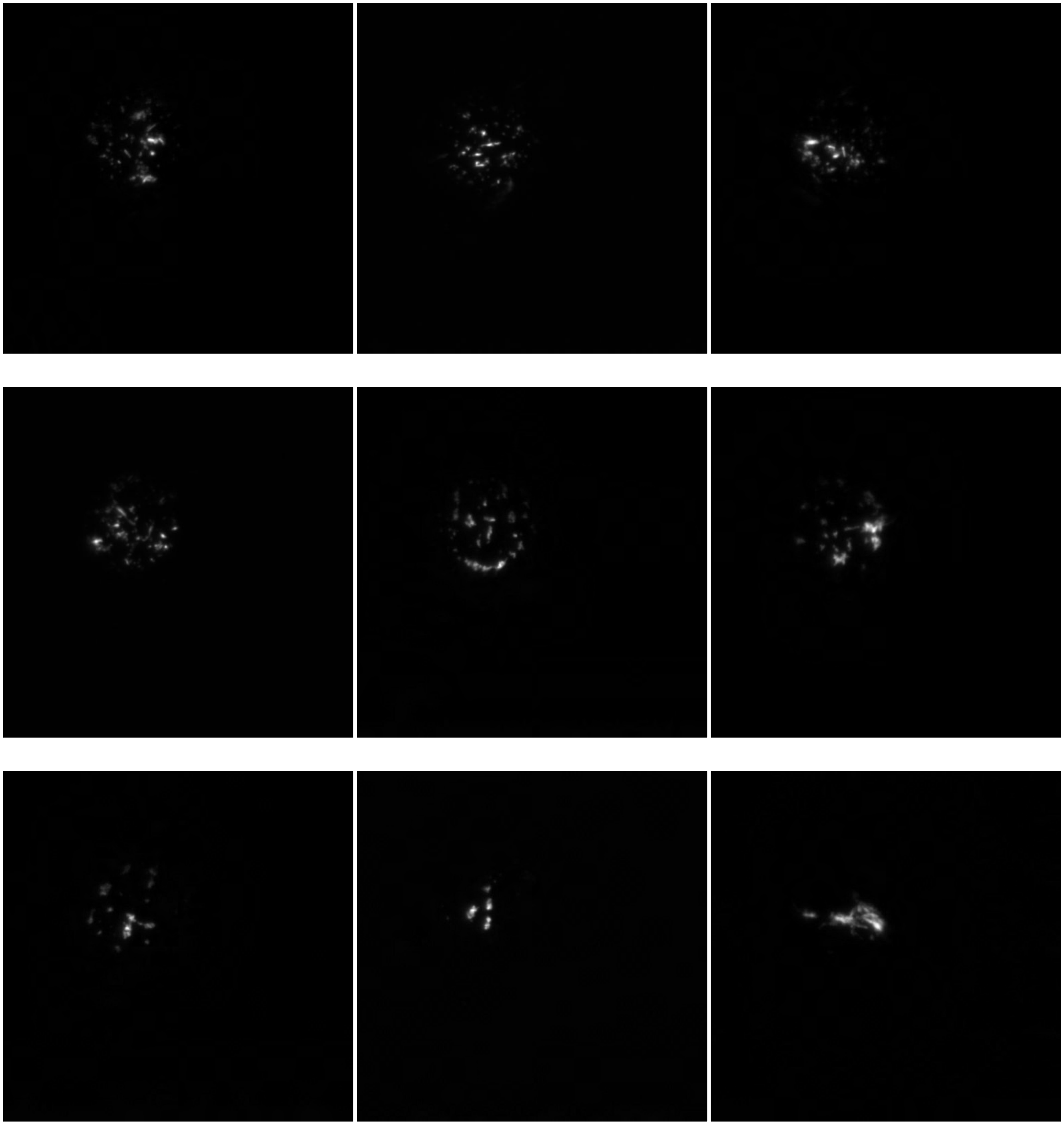

Figure S15. Microscopic images (50.5 μm × 50.5 μm) of emission from nanostructures for absorption polarization anisotropy.

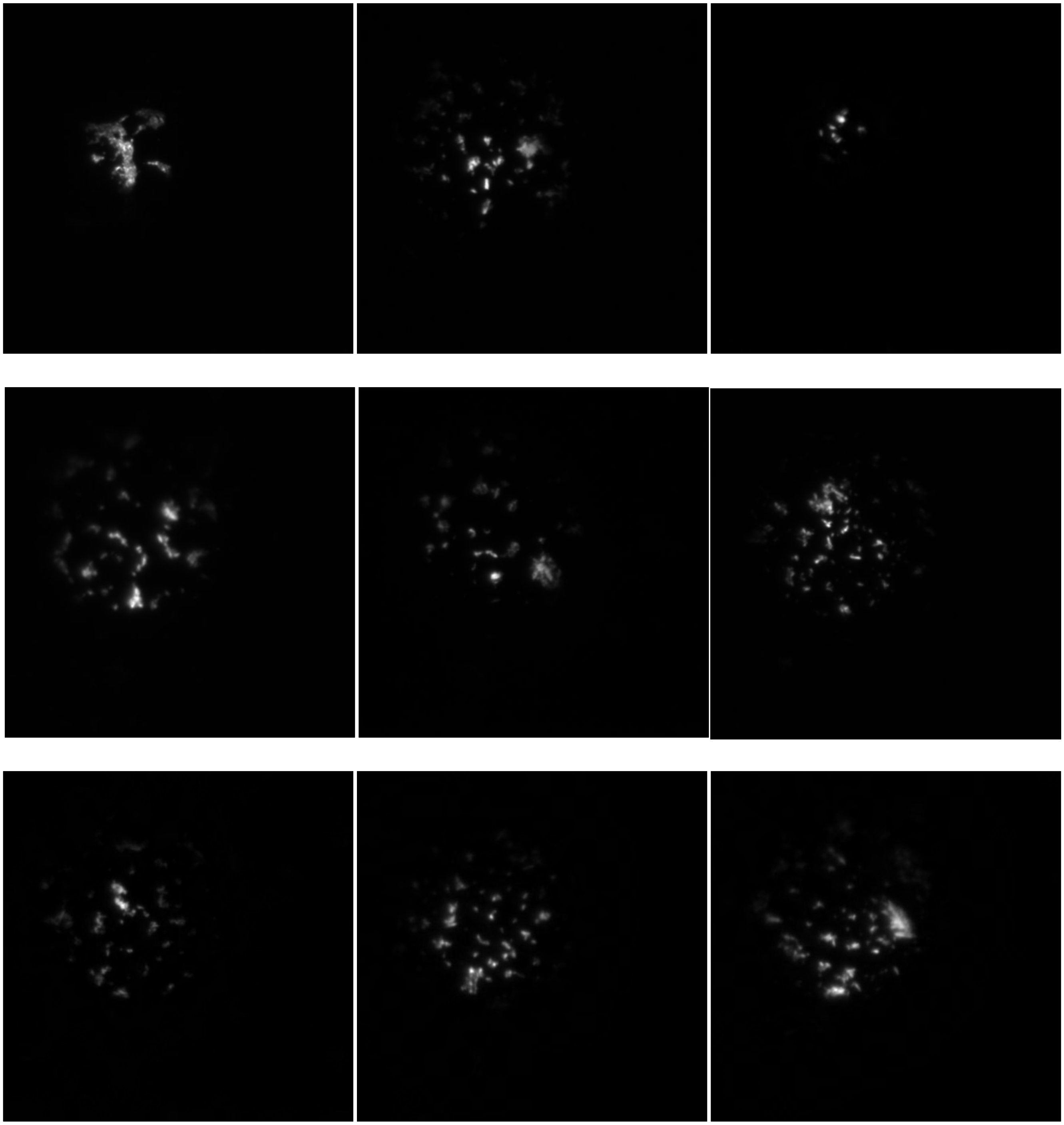

Figure S16. Microscopic images (50.5 μm × 50.5 μm) of emission from nanostructures for absorption polarization anisotropy.

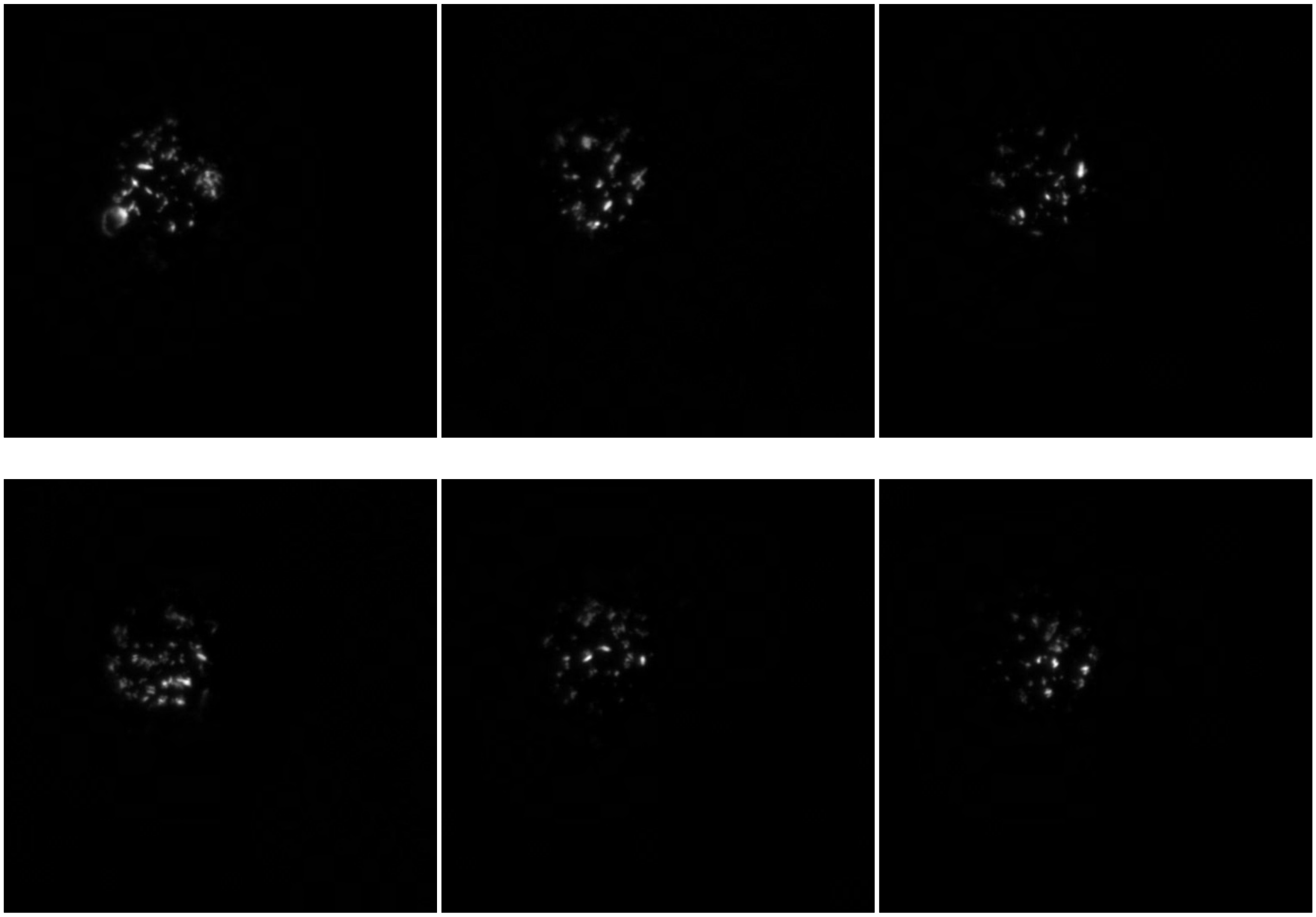

Figure S17. Microscopic images (50.5 µm × 50.5 µm) of emission from nanostructures for absorption polarization anisotropy.

15. Plots and Fitting Curves of Emission Intensity Changes

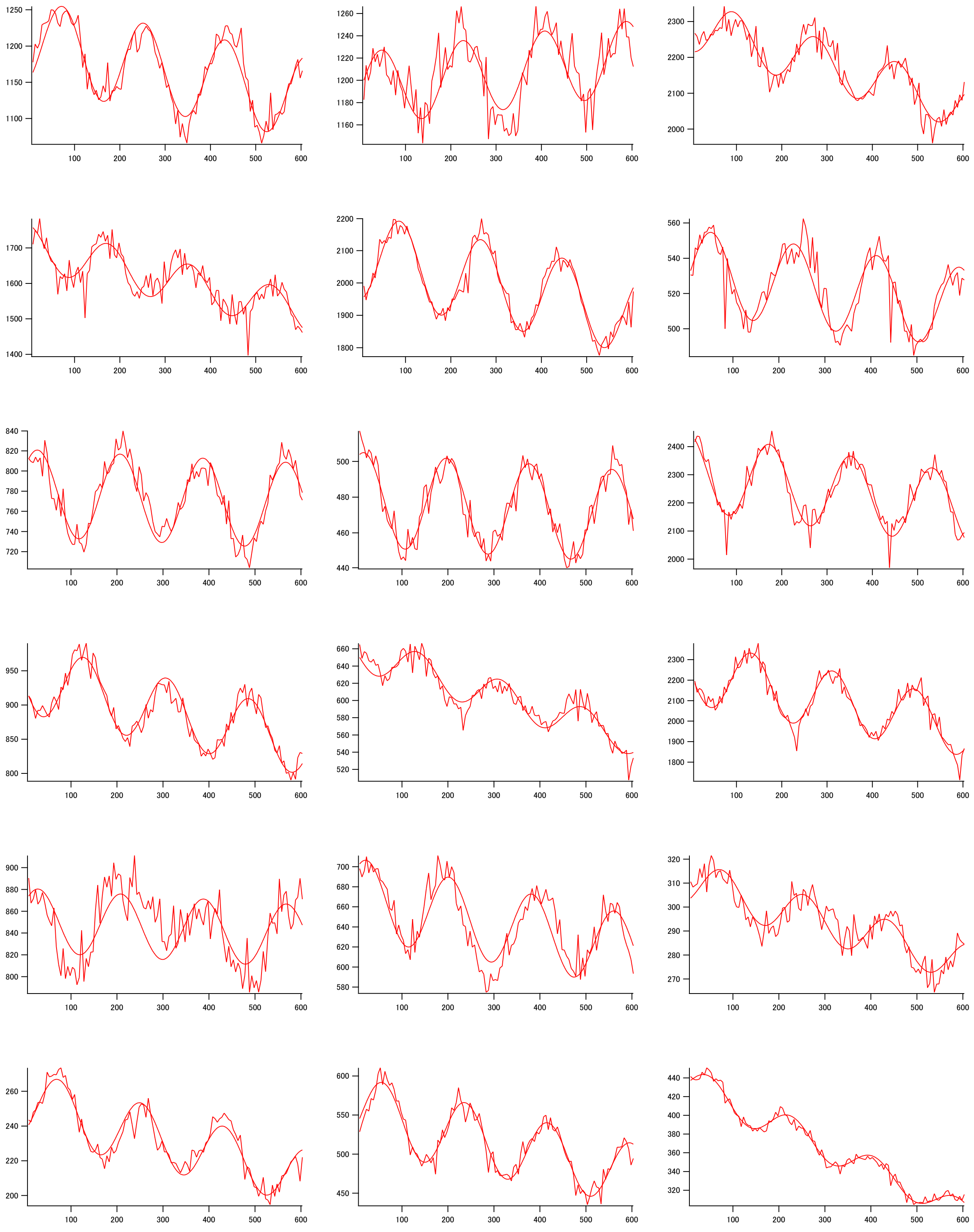

Figure S18. The plots and fitting curves of the emission intensity change versus the excitation light's orientation angle.

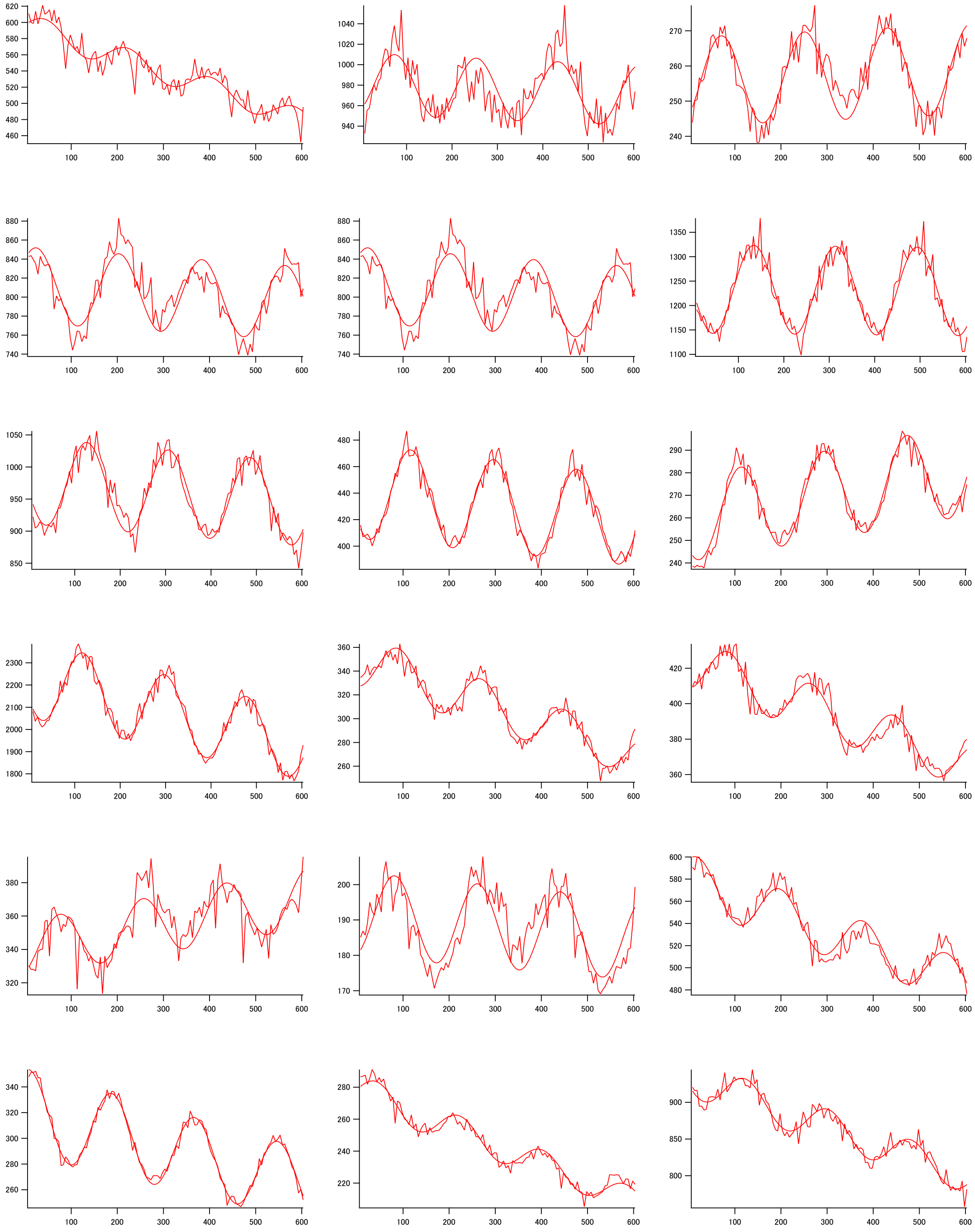

Figure S19. The plots and fitting curves of the emission intensity change versus the excitation light's orientation angle.

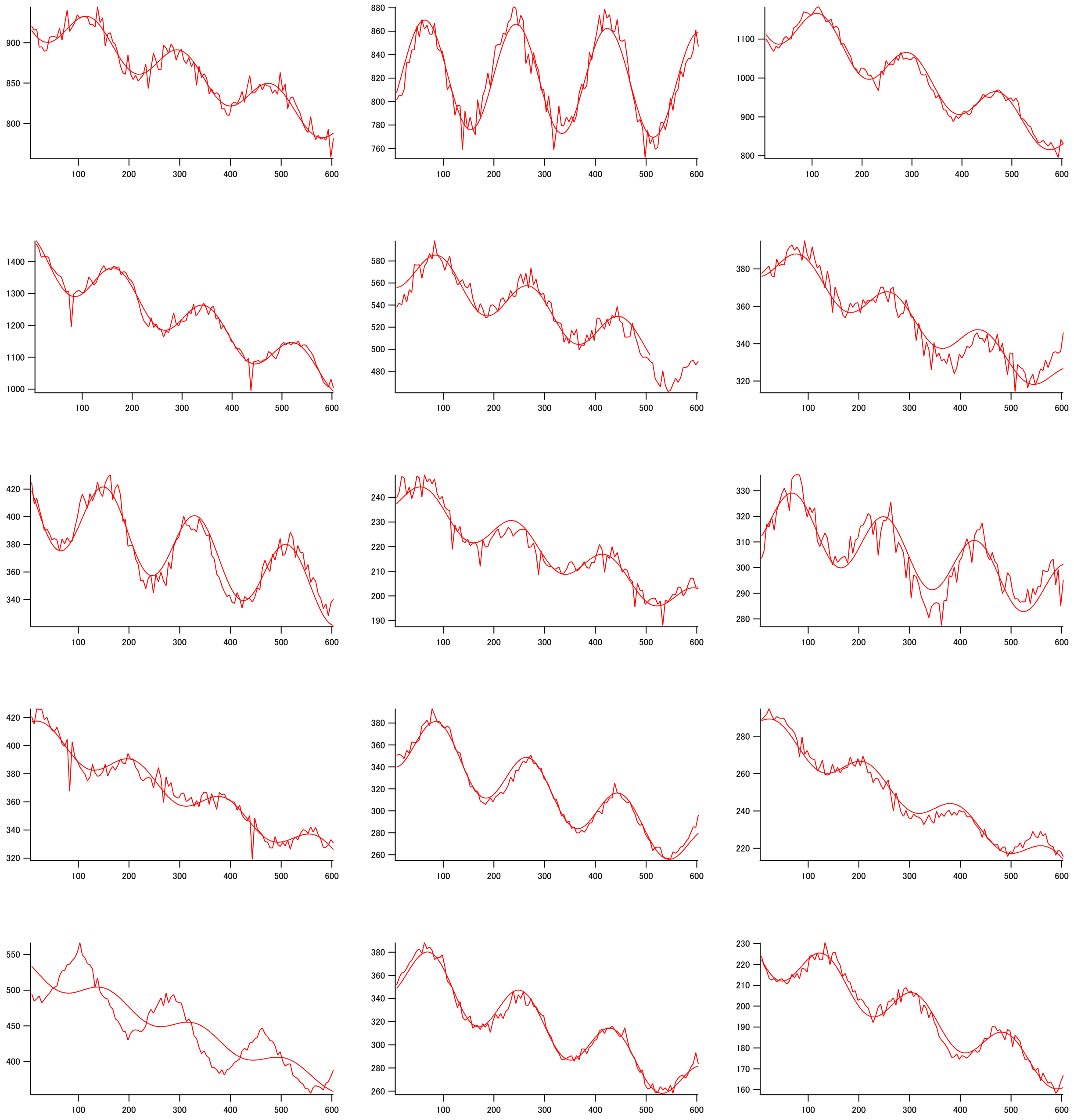

Figure S20. The plots and fitting curves of the emission intensity change versus the excitation light's orientation angle.

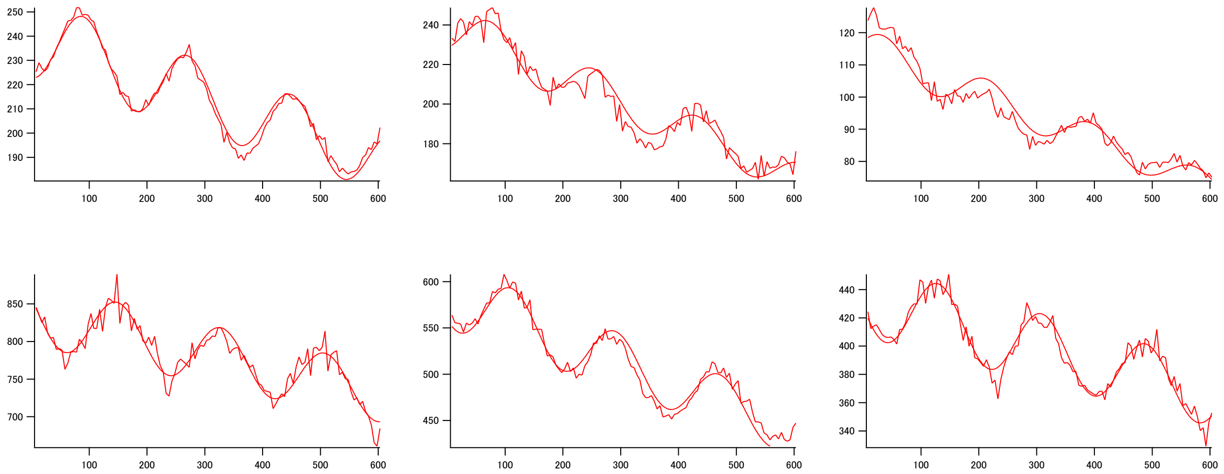

Figure S21. The plots and fitting curves of the emission intensity change versus the excitation light's orientation angle.

16. PXRD Patterns of Solid 1

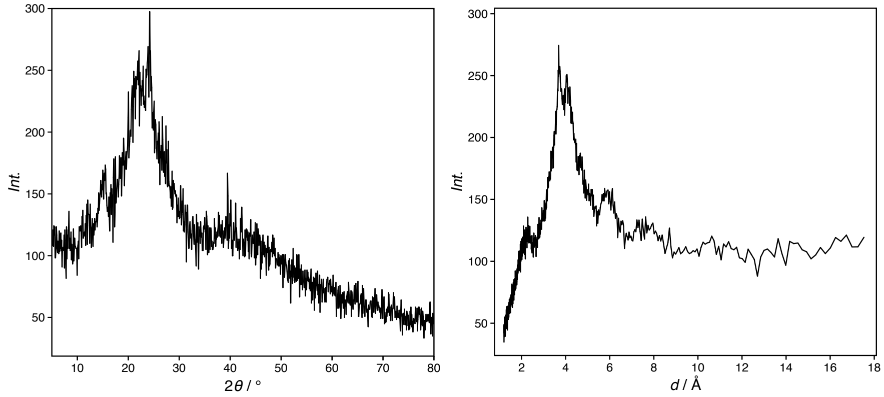

Figure S22. PXRD patterns of solid **1** from its toluene solution. (left) Plot of the intensity versus 2θ, and (right) d-spacing plot.

17. Photographs and Crystal Structure of 2

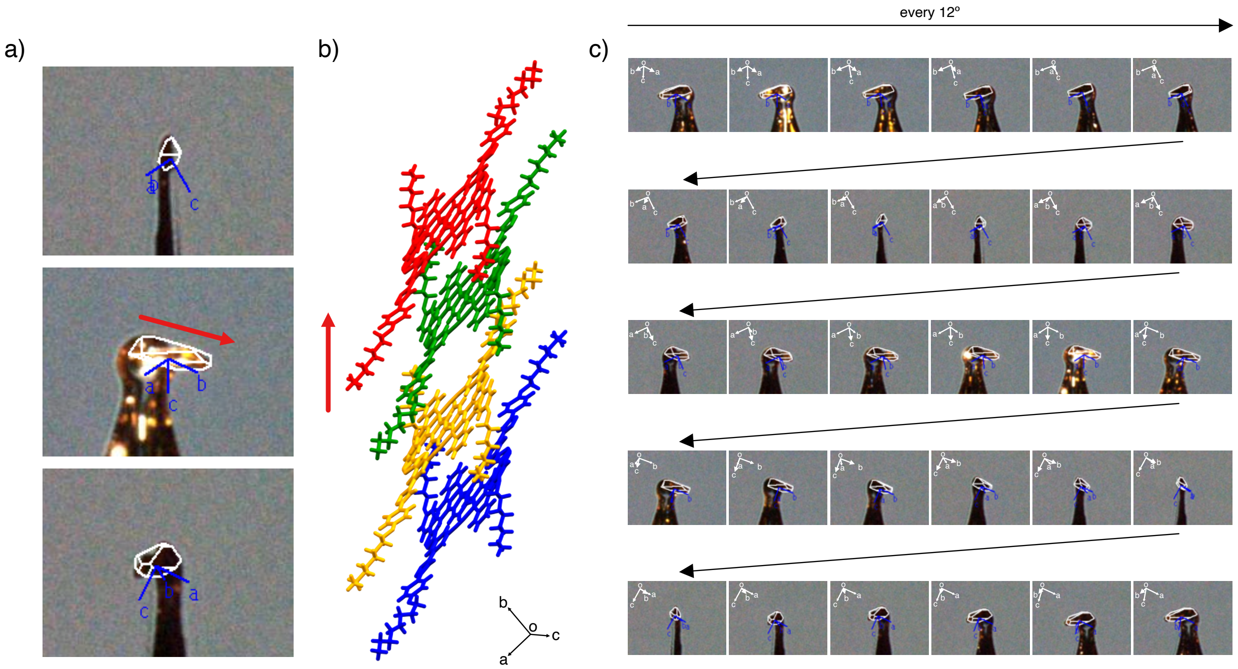

Figure S23. (a) Photographs of a single crystal of **2** (from top to bottom: rotation angles of 0°, 90°, and 180°). (b) Slip-stacked structure of the dimeric forms of **2**. The red arrow indicates the direction of the major axis of the crystal shown in the middle image in (a). (c) Sequential photographs taken at 12° intervals showing the rotation of the crystal from 0° to 360°. Note: The dimeric form of **2** is slip-stacked and tilted by ∠45° from the major axis of the crystal (Figure S23b). Thus, the transition dipole moments are oriented in the tilted alignment, consistent with the tilted alignment of the absorption anisotropy direction.

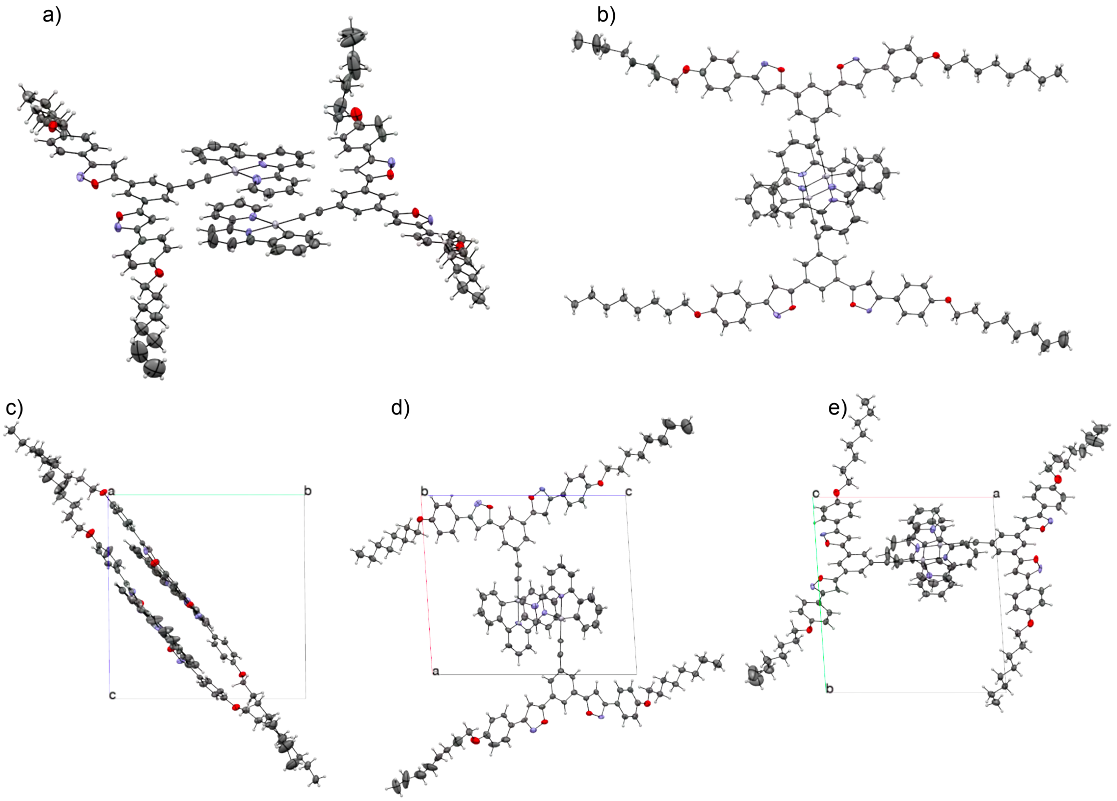

Figure S24. Crystal structures of **2**: (a) Side view, (b) top view, and (c, d, e) a-, b-, and c-axis projections.

Table S3. Crystallographic parameters of **2**.

Crystal	**2**
Formula	$C_{58}H_{58}N_4O_4Pt$
Formula weight	1070.17
Crystal system	triclinic
Space group	*P*–1
a/ Å	15.8388(6)
b/ Å	17.2592(7)
c/ Å	17.9659(8)
α/ º	89.332(3)
β/ º	86.333(3)
γ/ º	86.110(3)
V/ Å^3	4889.8(3)
$d_{calc.}$/ g cm^{-3}	1.454
Z	4
$2\theta_{max}$/ º	4.194° ≤ 2θ ≤ 50.7°
μ (MoKα)/ mm^{-1}	2.921
Temperature/ K	100
Crystal form	needle
Crystal size/ mm^3	0.13 × 0.03 × 0.01
Crystal color	Clear yellow
# of total reflections	67376
# of unique reflections	17778
R_{int}	0.1203
$R1(F_o)$	0.0614
$wR2(F_o^2)$	0.1600
G. O. F.	1.016
# of parameters used	1230
$\Delta\rho_{max}$/ eÅ^{-3}	2.935
$\Delta\rho_{min}$/ eÅ^{-3}	-1.349
CCDC number	2450131

18. TD-DFT Calculation of Dimer 2•2

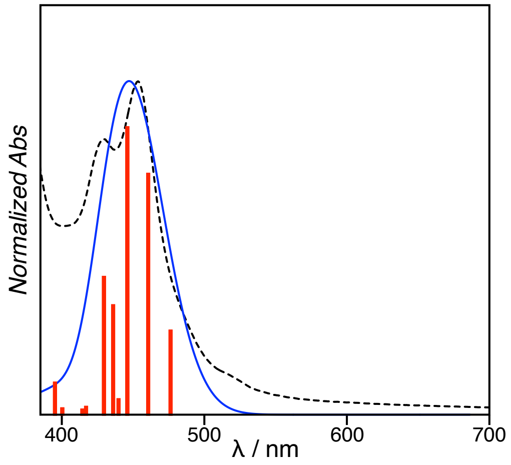

Figure S25. TD-DFT-predicted (M06/6-31g** + LanL2DZ) UV-vis absorption spectra of the crystal structure of dimeric **2** (blue solid line) and electron transitions (red lines) overlaid on the experimental UV-vis spectra (Black dashed line) of **1** (3.0 × 10^{-4} M) in toluene at 298 K. The predicted spectrum was rendered by using a Gaussian band shape of 0.1 eV. A shift factor of −43 nm was applied.

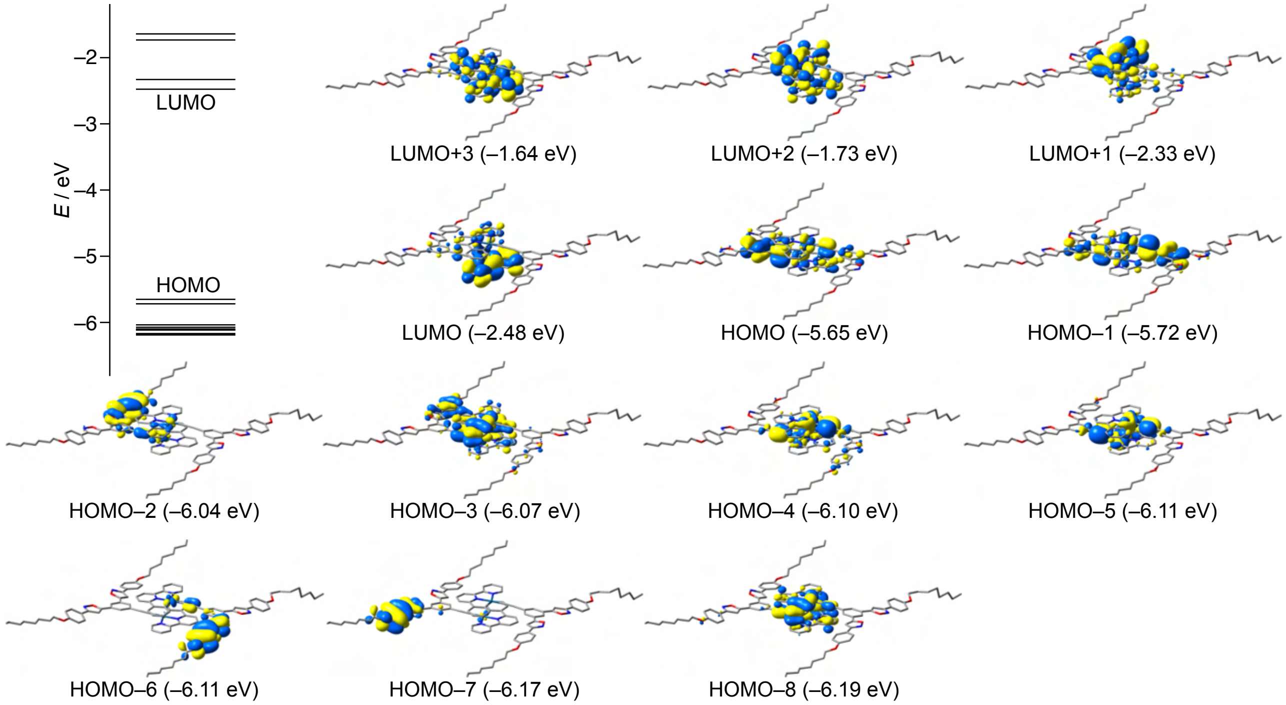

Figure S26. Energy diagram and orbital plots of the crystal structure of **2** calculated by TD-DFT at M06/6-31g** + LanL2DZ level; hydrogen atoms are omitted for clarity; isovalue 0.02.

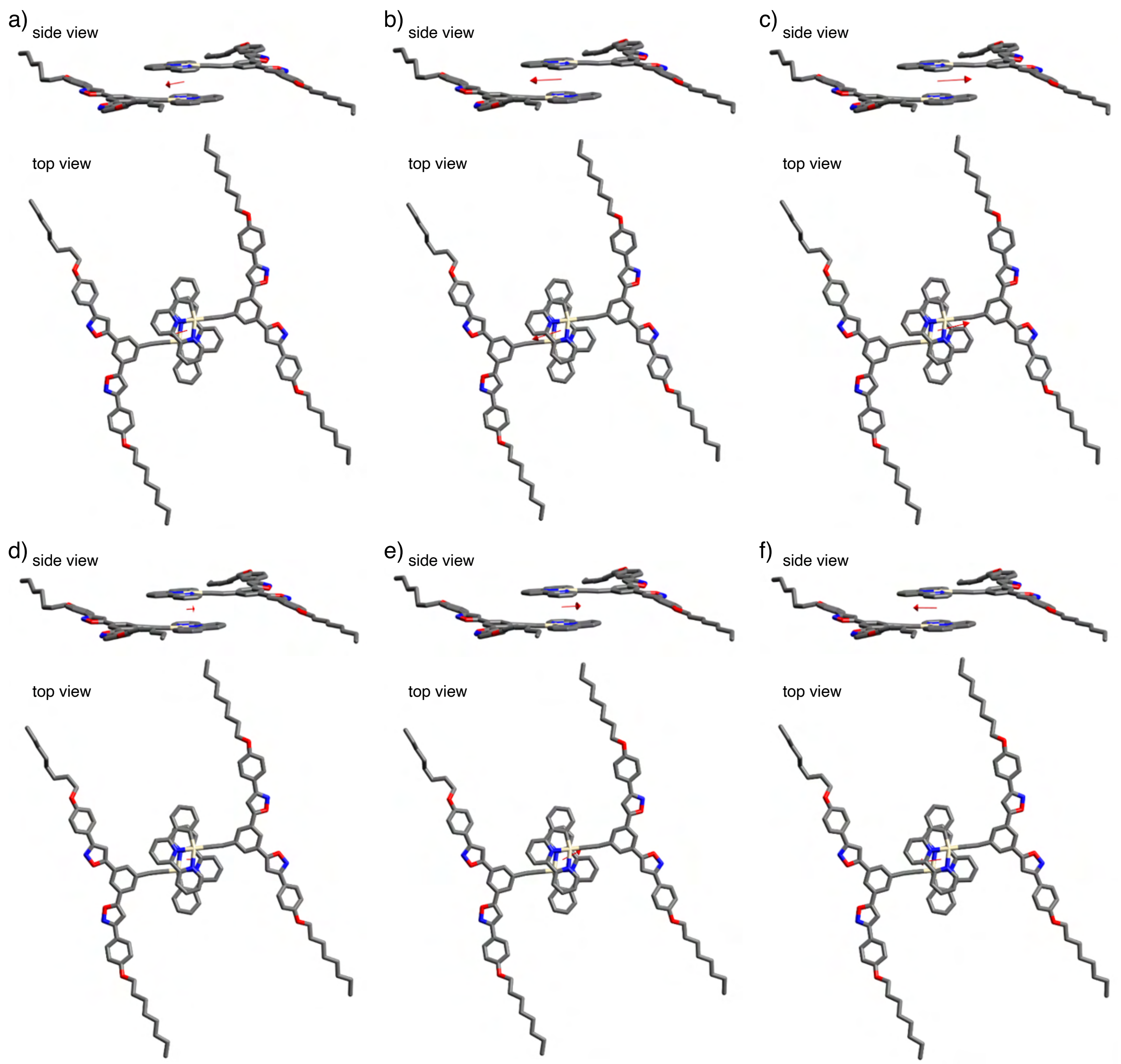

Figure S27. Transition dipole moments of the crystal structure of **2** in (a) excited state 1, (b) excited state 2, (c) excited state 3, (d) excited state 4, (e) excited state 5, and (f) excited state 6. The transition electric dipole moment vectors are shown in red.

Table S4. The electric dipole moments concerning transitions from a ground state to an excited state.

Excited State	*X*	*Y*	*Z*	*Dip. S.*	*Osc.*
1	0.1747	–0.6655	–0.0348	0.4746	0.0278
2	0.263	–1.1148	–0.0971	1.3214	0.0797
3	–0.3593	1.1812	0.0797	1.5307	0.0951
4	–0.1472	0.2422	0.0015	0.0803	0.0051
5	–0.0508	0.7495	0.077	0.5703	0.0362
6	0.3185	–0.7744	–0.0942	0.7101	0.0456

Table S5. TD-DFT-calculated singlet excited state data for of the crystal structure of **2**.†

Excited State	*E* (eV)	*λ* (nm)	*λ* –43 (nm)	*f*	*R* (10^{-40} cgs)
1	2.3875	519.31	476.31	0.0278	–0.6447
2	2.462	503.59	460.59	0.0797	–11.6341
3	2.5357	488.96	445.96	0.0951	–34.3514
4	2.568	482.81	439.81	0.0051	8.1964
5	2.5884	479	436.00	0.0362	–5.8323
6	2.6236	472.57	429.57	0.0456	42.9950
7	2.6952	460.02	417.02	0.0026	–6.2477
8	2.7104	457.44	414.44	0.0017	–1.7129
9	2.7966	443.34	400.34	0.0021	1.0099
10	2.8294	438.21	395.21	0.0106	1.5948
11	2.9097	426.1	383.10	0.0011	–0.4494
12	2.9133	425.57	382.57	0.0011	1.9824
13	2.9649	418.17	375.17	0.0029	–3.1421
14	3.0145	411.3	368.3	0.002	2.7231
15	3.1369	395.25	352.25	0.0141	0.1095
16	3.2057	386.76	343.76	0.0077	–7.7067
17	3.2118	386.03	343.03	0.0106	–6.8742
18	3.2308	383.75	340.75	0.0015	1.6013
19	3.2378	382.92	339.92	0.0213	53.4427
20	3.2987	375.86	332.86	0.0009	0.4089
21	3.3264	372.73	329.73	0.1244	–28.5200
22	3.363	368.67	325.67	0.0001	0.0545
23	3.3642	368.54	325.54	0.0002	–0.3096
24	3.3882	365.93	322.93	0.016	10.3061
25	3.4341	361.04	318.04	0.0008	–0.7373
26	3.4573	358.61	315.61	0.0005	–0.6430
27	3.4632	358.01	315.01	0.0094	0.0893
28	3.4737	356.92	313.92	0.1072	7.8816
29	3.4918	355.08	312.08	0.0012	0.3198
30	3.4997	354.27	311.27	0.0677	7.1080

†Excited state energies (*E*), wavelengths (*λ*), oscillator strength (*f*), and rotatory strength (*R*) were calculated at the M06/6-31G** + LanL2DZ level of theory.

Table S6 Electron transitions of the excited states for the crystal structure of **2**.

Excited state	1	476.31 nm	CI coefficient
HOMO–1	->	LUMO	-0.19614
HOMO	->	LUMO	0.65621

Excited state	2	460.59 nm	CI coefficient
HOMO–8	->	LUMO	-0.10501
HOMO–3	->	LUMO	0.13595
HOMO–1	->	LUMO	0.63688
HOMO	->	LUMO	0.19062
HOMO	->	LUMO+1	0.11189

Excited state	3	445.96 nm	CI coefficient
HOMO–3	->	LUMO+1	-0.11265
HOMO	->	LUMO	-0.10325
HOMO	->	LUMO+1	0.65176

Excited state	4	439.81 nm	CI coefficient
HOMO–5	->	LUMO	0.53216
HOMO–4	->	LUMO	-0.40283
HOMO–2	->	LUMO	0.11143
HOMO	->	LUMO+1	0.117

Excited state	5	436.00 nm	CI coefficient
HOMO–8	->	LUMO	-0.31336
HOMO–3	->	LUMO	0.46188
HOMO–2	->	LUMO	0.19353
HOMO–1	->	LUMO	-0.18338
HOMO–1	->	LUMO+1	-0.27495

Excited state	6	429.57 nm	CI coefficient
HOMO–8	->	LUMO	-0.16578
HOMO–3	->	LUMO	0.19197
HOMO–1	->	LUMO+1	0.62997

Excited state	7	417.02 nm	CI coefficient
HOMO–5	->	LUMO	0.25499
HOMO–5	->	LUMO+1	0.32447
HOMO–4	->	LUMO	0.33073
HOMO–4	->	LUMO+1	0.41066
HOMO–3	->	LUMO+1	-0.11738

Excited state	8	414.44 nm	CI coefficient
HOMO–8	->	LUMO	0.22287
HOMO–8	->	LUMO+1	0.26484
HOMO–6	->	LUMO+1	-0.10506
HOMO–3	->	LUMO	0.15347
HOMO–3	->	LUMO+1	0.48453
HOMO–2	->	LUMO+1	0.18534
HOMO	->	LUMO+1	0.15677

Excited state	9	400.34 nm	CI coefficient
HOMO–5	->	LUMO	0.32846
HOMO–5	->	LUMO+1	-0.25515
HOMO–4	->	LUMO	0.41553
HOMO–4	->	LUMO+1	-0.33625

Excited state	10	395.21 nm	CI coefficient
HOMO–8	->	LUMO	0.52483
HOMO–8	->	LUMO+1	-0.15849
HOMO–3	->	LUMO	0.31682
HOMO–3	->	LUMO+1	-0.21473
HOMO–2	->	LUMO	0.13037

Excited state	11	383.10 nm	CI coefficient
HOMO–10	->	LUMO	0.10024
HOMO–10	->	LUMO+1	-0.12845

HOMO–5	->	LUMO+1	0.51739
HOMO–4	->	LUMO+1	-0.39934
HOMO–2	->	LUMO+1	0.12311

Excited state	12	382.57 nm	CI coefficient
HOMO–13	->	LUMO	-0.30392
HOMO–11	->	LUMO	-0.14189
HOMO–10	->	LUMO	0.57835
HOMO–5	->	LUMO+1	-0.143

Excited state	13	375.17 nm	CI coefficient
HOMO–8	->	LUMO+1	0.5867
HOMO–3	->	LUMO+1	-0.2907
HOMO–2	->	LUMO+1	-0.11437

Excited state	14	368.30 nm	CI coefficient
HOMO–13	->	LUMO	0.13043
HOMO–13	->	LUMO+1	0.18733
HOMO–11	->	LUMO+1	-0.16276
HOMO–10	->	LUMO	0.1264
HOMO–10	->	LUMO+1	0.61004
HOMO–5	->	LUMO+1	0.10902

Excited state	15	352.25 nm	CI coefficient
HOMO–1	->	LUMO+2	-0.19079
HOMO	->	LUMO+2	0.64893

Excited state	16	343.76 nm	CI coefficient
HOMO–13	->	LUMO	-0.20105
HOMO–1	->	LUMO+2	0.60789
HOMO	->	LUMO+2	0.14694
HOMO	->	LUMO+3	-0.18725

Excited state	17	343.03 nm	CI coefficient
HOMO–13	->	LUMO	0.54931
HOMO–13	->	LUMO+1	-0.15028
HOMO–10	->	LUMO	0.2805
HOMO–10	->	LUMO+1	-0.12115
HOMO–1	->	LUMO+2	0.20175
HOMO	->	LUMO+2	0.1138

Excited state	18	340.75 nm	CI coefficient
HOMO–3	->	LUMO	-0.25933
HOMO–2	->	LUMO	0.62612
HOMO	->	LUMO+3	0.10777

Excited state	19	339.92 nm	CI coefficient
HOMO–2	->	LUMO	-0.11742
HOMO–1	->	LUMO+2	0.17794
HOMO	->	LUMO+3	0.63489

Excited state	20	332.86 nm	CI coefficient
HOMO–6	->	LUMO	0.67563
HOMO–4	->	LUMO	0.13068
HOMO–3	->	LUMO	0.10292

Excited state	21	329.73 nm	CI coefficient
HOMO–1	->	LUMO+3	0.67257

Excited state	22	325.67 nm	CI coefficient
HOMO–7	->	LUMO	0.57268
HOMO–3	->	LUMO+1	0.15284
HOMO–2	->	LUMO+1	-0.3589

Excited state	23	325.54 nm	CI coefficient
HOMO–7	->	LUMO	0.39282
HOMO–3	->	LUMO+1	-0.21861
HOMO–2	->	LUMO+1	0.52445

Excited state	24	322.93 nm	CI coefficient
HOMO–13	->	LUMO	0.16003
HOMO–13	->	LUMO+1	0.62828
HOMO–10	->	LUMO+1	-0.21126

Excited state	25	318.04 nm	CI coefficient
HOMO–8	->	LUMO+2	0.12413
HOMO–6	->	LUMO+2	-0.15595
HOMO–5	->	LUMO+2	0.36839
HOMO–5	->	LUMO+3	0.10474
HOMO–4	->	LUMO+2	0.49185
HOMO–4	->	LUMO+3	0.13129
HOMO–3	->	LUMO+2	0.14523

Excited state	26	315.61 nm	CI coefficient
HOMO–12	->	LUMO	-0.18647
HOMO–9	->	LUMO	0.67023

Excited state	27	315.01 nm	CI coefficient
HOMO–6	->	LUMO+1	0.64003
HOMO–4	->	LUMO+1	0.12933
HOMO–3	->	LUMO+2	-0.16203

Excited state	28	313.92 nm	CI coefficient
HOMO–8	->	LUMO+2	0.16009
HOMO–8	->	LUMO+3	0.11783
HOMO–6	->	LUMO+1	0.20139
HOMO–5	->	LUMO+3	-0.13166
HOMO–4	->	LUMO+2	-0.24173
HOMO–3	->	LUMO+2	0.46415
HOMO–2	->	LUMO+2	0.22536

Excited state	29	312.08 nm	CI coefficient
HOMO–7	->	LUMO+1	0.69007

Excited state	30	311.27 nm	CI coefficient
HOMO–8	->	LUMO+2	-0.164
HOMO–5	->	LUMO+2	0.39444
HOMO–5	->	LUMO+3	-0.31839
HOMO–4	->	LUMO+2	-0.20679
HOMO–4	->	LUMO+3	0.26709
HOMO–3	->	LUMO+2	-0.11748
HOMO–3	->	LUMO+3	-0.11684
HOMO–2	->	LUMO+3	-0.1178

Table S7. Cartesian coordinates of dimer **2•2** obtained the crystal structure analysis and employed in a TD-DFT calculation.

Pt	6.239	13.079	6.345
O	-2.327	11.797	4.393
O	-2.651	14.491	8.348
O	-2.19	6.118	-0.758
N	8.195	12.807	6.175
O	-3.604	18.909	14.54
N	6.952	14.466	7.756
C	4.312	13.265	6.499
N	-2.777	10.947	3.356
N	-3.279	15.217	9.395
C	8.96	15.276	8.771
H	9.909	15.272	8.821
C	-2.597	16.524	11.288
C	-1.954	18.148	12.952
H	-1.282	18.703	13.331
C	-0.359	12.432	5.597
C	6.21	11.668	4.934
C	-0.992	11.634	4.568
C	6.236	15.258	8.579
H	5.288	15.231	8.529
C	-3.225	18.147	13.477
C	1.022	12.484	5.667
H	1.538	11.991	5.039
C	-2.286	15.72	10.112
C	-0.786	7.504	0.656
H	-0.01	7.002	0.434
C	-1.129	13.161	6.524
H	-2.079	13.142	6.48
C	-1.7	10.317	2.951
C	7.526	11.257	4.536
C	6.845	16.115	9.504
H	6.334	16.689	10.064
C	-1.658	17.339	11.872
H	-0.776	17.348	11.521
C	8.312	14.448	7.88
C	-0.568	10.715	3.668
H	0.322	10.404	3.546
C	-0.489	13.906	7.501
C	1.674	13.242	6.637
C	9.016	13.493	6.974
C	8.61	11.893	5.269
C	0.904	13.95	7.562
H	1.332	14.464	8.237
C	-1.99	7.185	0.084
C	-3.009	8.986	1.277
H	-3.777	9.505	1.484
C	5.145	11.071	4.254
H	4.256	11.347	4.446
C	3.107	13.306	6.605
C	-0.693	8.538	1.545
H	0.147	8.745	1.938
C	8.256	16.081	9.561
H	8.709	16.644	10.177
C	-1.808	9.288	1.879
C	-1.302	14.627	8.491
C	-1.088	5.245	-1.006
H	-0.762	4.86	-0.154
H	-0.346	5.746	-1.427
C	0.934	-0.16	-5.891
H	1.613	0.509	-6.154
H	1.367	-0.812	-5.284
C	-3.115	7.946	0.382
H	-3.949	7.75	-0.029
C	0.25	1.412	-3.973
H	0.676	0.835	-3.289
H	0.931	2.054	-4.293
C	5.359	10.091	3.313
H	4.612	9.683	2.892
C	-0.42	3.225	-2.302
H	-0.083	2.764	-1.493
H	0.324	3.755	-2.684
C	-0.208	0.542	-5.146
H	-0.703	1.11	-5.786
H	-0.835	-0.144	-4.805
C	-1.547	4.15	-1.917
H	-1.935	4.549	-2.736
H	-2.258	3.628	-1.467
C	-0.885	2.176	-3.335
H	-1.491	1.533	-2.886
H	-1.402	2.632	-4.044
C	0.451	-0.875	-7.114
H	-0.1	-0.269	-7.65
H	-0.083	-1.653	-6.848
H	1.22	-1.173	-7.643
C	6.607	9.691	2.974
H	6.724	9.014	2.318
C	-3.845	16.509	11.849
H	-4.501	15.916	11.504
C	7.704	10.26	3.571
H	8.579	9.978	3.331
C	10.819	12.251	6.022
H	11.747	12.053	5.982
C	-4.173	17.337	12.907
H	-5.062	17.343	13.242
C	10.349	13.202	6.955
H	10.949	13.629	7.555
C	9.967	11.616	5.185
H	10.293	10.992	4.549
C	-1	15.358	9.545
H	-0.136	15.594	9.863
Pt	7.958	11.776	9.998
O	16.488	13.256	11.953
O	16.848	10.162	8.263
O	16.089	17.756	18.144
O	17.423	5.565	2.121
N	5.999	11.999	10.092
N	16.915	14.055	12.979
N	7.313	10.398	8.563
N	17.494	9.424	7.263
C	15.524	10.009	8.154
C	7.909	13.172	11.41
C	6.598	13.598	11.707
C	5.2	11.266	9.294
C	12.512	11.553	9.855
C	11.106	11.569	9.773
C	18.053	8.001	4.798
H	18.734	8.582	5.115
C	15.15	13.068	12.032
C	6.124	8.736	6.691
H	5.725	8.165	6.046
C	5.94	10.354	8.412
C	9.89	11.625	9.891
C	14.663	13.727	13.108
H	13.76	13.77	13.399
C	5.513	12.951	11.005
C	13.316	10.795	8.954
H	12.906	10.311	8.246
C	18.477	4.594	0.256
H	17.903	5.057	-0.405
H	18	3.779	0.55
C	7.491	8.777	6.84
H	8.043	8.222	6.301
C	15.276	9.156	7.137
H	14.43	8.839	6.845
C	8.059	9.609	7.76
H	9.005	9.634	7.838
C	8.943	13.808	12.113
H	9.839	13.54	11.941
C	13.954	22.923	20.944
H	13.583	23.385	20.152
H	13.225	22.4	21.363
C	14.525	12.248	10.997
C	13.164	12.285	10.832
H	12.649	12.833	11.413
C	15.774	14.331	13.699
C	15.83	15.204	14.883
C	16.814	7.971	5.445
C	16.093	6.308	3.871
H	15.418	5.713	3.564
C	15.3	11.472	10.128
H	16.243	11.432	10.239
C	14.695	10.765	9.108
C	15.063	21.973	20.5
H	15.482	21.563	21.297
H	15.761	22.487	20.02
C	14.461	23.953	21.932
H	15.131	24.522	21.478
H	14.921	23.481	22.671
C	4.165	13.127	11.067
H	3.792	13.758	11.671
C	14.679	15.786	15.368
H	13.849	15.609	14.943
C	15.858	7.107	4.957
H	15.012	7.063	5.387
C	19.511	3.244	-1.61
H	18.911	3.689	-2.259
H	19.058	2.424	-1.287
C	19.765	4.188	-0.418
H	20.35	3.735	0.241
H	20.238	4.998	-0.737
C	17.034	15.476	15.526
H	17.833	15.072	15.209
C	18.301	7.197	3.706
H	19.153	7.211	3.285
C	17.089	16.315	16.614
H	17.92	16.485	17.043
C	6.372	14.602	12.652
H	5.482	14.874	12.846
C	3.843	11.47	9.344
H	3.263	10.989	8.766
C	17.302	6.369	3.225
C	15.573	19.764	19.327
H	16.373	20.162	18.901
H	15.851	19.37	20.192
C	7.411	15.191	13.292
H	7.242	15.879	13.925
C	5.349	9.54	7.489
H	4.403	9.529	7.4
C	16.544	8.836	6.598
C	15.02	18.676	18.436
H	14.279	18.204	18.894
H	14.669	19.068	17.597
C	14.533	20.861	19.58
H	13.728	20.457	19.991
H	14.264	21.258	18.714
C	14.716	16.638	16.485
H	13.914	17.018	16.824
C	3.339	12.373	10.239
H	2.398	12.489	10.298
C	13.389	24.848	22.526
H	12.678	24.281	22.918
H	12.98	25.383	21.799
C	8.721	14.8	13.038
H	9.446	15.211	13.492
C	13.914	25.796	23.601
H	14.56	26.417	23.202
H	14.353	25.278	24.307
H	13.168	26.304	23.983
C	15.925	16.918	17.086
C	18.671	5.486	1.429
H	19.369	5.118	2.025
H	18.954	6.386	1.13
C	20.845	2.853	-2.318
H	21.343	3.678	-2.546
H	21.4	2.325	-1.693
C	20.619	2.047	-3.582
H	20.159	1.201	-3.356
H	20.038	2.558	-4.2
C	21.627	1.146	-5.755
H	22.469	0.876	-6.177
H	21.194	1.835	-6.3
H	21.035	0.368	-5.682
C	21.933	1.742	-4.258
H	22.441	1.08	-3.727
H	22.476	2.568	-4.328
C	-6.9007	23.4219	21.7081
C	-6.412	22.275	20.804
C	-6.044	22.601	19.515
C	-5.487	21.451	18.601
C	-5.409	22.098	16.944
C	-4.844	21.032	15.962
C	-3.454	20.732	16.066
C	-2.745	19.875	15.013
H	-6.1854	24.2175	21.693
H	-7.8424	23.782	21.3497
H	-7.0139	23.063	22.7097
H	-5.5691	21.8191	21.28
H	-7.1939	21.5467	20.7472
H	-6.9003	23.0183	19.0277
H	-5.2945	23.3625	19.5732
H	-4.5091	21.1653	18.9282
H	-6.1445	20.6075	18.6334
H	-4.7743	22.9594	16.9319
H	-6.392	22.3804	16.6296
H	-5.3887	20.1236	16.1137
H	-5.0337	21.3702	14.9647
H	-2.9361	21.6675	16.1056
H	-3.3102	20.2477	17.0092
H	-1.8905	19.4055	15.4538
H	-2.4294	20.4976	14.2021

19. Time-Lapse CLSM Experiments of complex 1

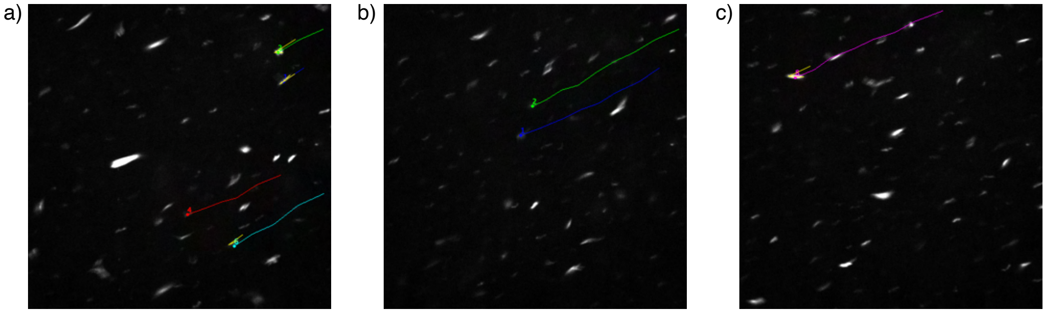

Figure S28. Time-lapse (at 200 ms intervals) CLSM images (82 µm × 82 µm) of a toluene solution of **1** (3.0×10^{-4} M) under the flow condition (λ_{ex} = 488 nm). Some colored lines are tracking lines of the trajectory of particles made by complex **1** (Supplementary Movie S1).

Table S8. The calculated velocity of particles observed in the CLSM images in Figure S28.

27.551	28.091	20.754	21.466	26.12	23.786	32.398
26.833	28.375	22.542	25.412	23.324	31.128	21.541
26.497	27.2	23.637	15.94	20.396	31.2	22.285
28.678	19.233	21.136	17.978	31.159	28.091	26.315
23.296	20.861	23.637	18.26	22.285	32.917	38.666
22.909	21.121	23.624	16.08	27.306	33.988	41.608
22.242	31.88	28.375	21.136	24.423	26.593	31.516
24.697	31.516	29.12	19.677	20.754	31.648	28.844
30.061	32.13	29.6	20	26.156	29.729	29.339
31.2	27.943	28.588	17.307	19.677	29.35	42.791
20.999	31.128	26.593	17.564	20.396	35.822	27.586
29.087	26.156	28.678	20.8	9.765	32.653	31.118
28.375	26.892	22.069	17.763	19.083	28.999	30.061
34.437	25.057	25.044	22.285	18.608	27.989	32.683
26.833	33.275	21.121	20.048	27.736	30.411	27.527
28.273	21.967	26.892	23.324	26.058	23.296	27.632
23.324	27.586	14.311	22.542	24.331	25.525	31.516
26.485	27.943	16.492	27.212	32.839	19.333	32.917
27.586	21.121	24.067	20.048	34.363	26.156	
22.185	24.331	18.243	23.255	27.306	34.465	

Average Velocity = 25 ± 5 µm s^{-1}

Table S9. The measured angle of particles observed in the CLSM images in Figure S28.

12.243	20.719	24.746	44.762	12.756	49.255	30.63
2.91	0.439	17.837	47.818	23.704	31.506	12.341
30.845	21.683	15.43	49.111	72.791	51.35	45.749
2.643	16.563	28.789	12.445	84.017	28.19	35.176
17.914	21.778	20.225	15.544	75.825	24.896	AVERAGE
11.657	12.873	3.994	6.093	22.21	25.133	23.2011371
14.604	13.435	10.101	9.058	13.561	0.723	STDEV
33.369	20.102	22.302	6.891	14.14	13.707	17.4816913
7.567	34.315	2.991	22.554	1.964	7.024	
2.249	26.602	11.903	15.504	46.355	18.902	
8.279	21.148	5.753	9.991	65.072	10.181	
30.444	28.035	1.598	34.481	88.394	23.464	
9.677	23.405	16.147	8.495	71.166	23.487	
27.022	26.712	45.979	31.002	33.671	17.554	
21.386	1.967	34.211	23.752	26.028	48.141	
27.617	1.941	42.247	19.504	26.338	36.254	
36.254	2.768	4.715	28.254	20.047	29.934	
14.467	8.15	32.404	20.353	27.449	33.293	
1.864	0.464	21.829	7.914	38.547	22.492	
21.795	0.85	31.007	3.944	34.528	8.563	

Average Angle (θ_0) = 23 °

Note: The equation of the 2D order parameter (S) is given as $S = \langle 2(\cos\theta - \theta_0)^2 - 1 \rangle$.[69-71] An order parameter of 0.84119 was determined on based on the data in Table S9.

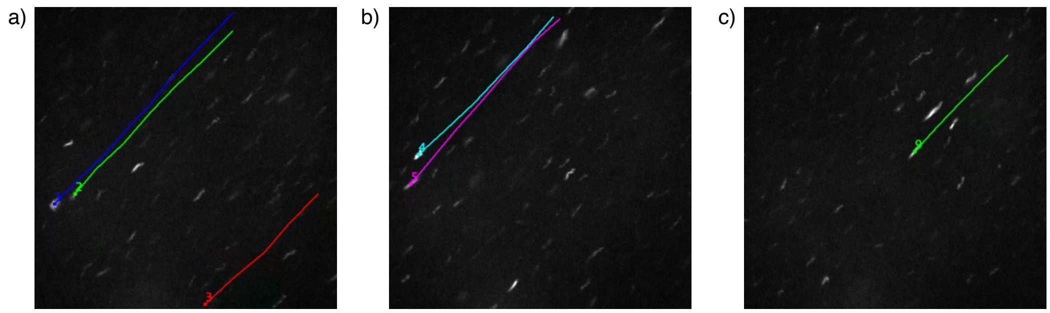

Figure S29. Time-lapse (at 200 ms intervals) CLSM images (82 μm × 82 μm) of a toluene solution of **1** (3.0×10^{-4} M) under the flow condition (λ_{ex} = 488 nm). Some colored lines are tracking lines of the trajectory of particles made by complex **1** (Supplementary Movie S2)

Table S10. The calculated velocity of particles observed in the CLSM images in Figure S29.

44.864	37.609	35.236	49.793	40.358
38.166	47.524	58.842	51.505	39.663
36.389	40.871	62.267	54.898	53.223
34.88	50.729	47.227	48.702	35.679
38.88	38.608	51.505	45.991	45.481
42.241	41.299	44.779	47.037	48.662
32.653	41.291	42.46	61.188	40.358
38.483	40.2	46.4	50.501	57.472
44.978	39.135	56.569	44.181	42.88
43.65	33.495	49.244	48.859	45.824
46.955	36.177	49.793	49.986	46.386
35.072	28.9	56.143	42.88	44.181
41.876	29.12	52.636	52.055	41.299
40.729	35.723	42.611	48.352	48.087
39.663	32.829	46.441	56.074	45.269
40.176	41.922	56.006	42.611	46.441
38.241	36.878	50.501	42.46	49.12
37.609	39.036	57.361	41.492	37.905
33.294	41.492	50.375	43.081	
47.531	34.733	56.143	49.793	

Average Velocity = 44 ± 7 μm s^{-1}

Table S11. The measured angle of particles observed in the CLSM images in Figure S29.

2.407	6.951	12.885	9.729
3.535	3.848	2.412	0.433
6.788	8.007	7.805	3.781
3.204	15.265	3.444	15.831
2.911	0.407	2.543	4.458
9.707	3.551	6.396	0.709
8.549	22.852	8.507	7.69
4.087	8.633	8.017	11.489
9.656	16.82	3.978	5.425
5.446	8.009	4.648	5.329
2.041	3.38	11.813	2.506
3.921	4.976	15.034	17.324
4.634	5.978	5.793	13.522
1.869	10.637	7.239	0.574
7.498	3.804	8.977	AVERAGE
4.379	2.53	8.418	6.74317568
9.28	1.616	8.135	STDEV
9.056	1.808	7.143	4.43832364
7.23	3.527	12.095	
7.885	2.728	5.503	

Average Angle (θ_0) = 7 °

Note: The equation of the 2D order parameter (S) is given as $S = \langle 2(\cos\theta - \theta_0)^2 - 1\rangle$.[69-71] An order parameter of 0.9881 was determined on based on the data in Table S11.

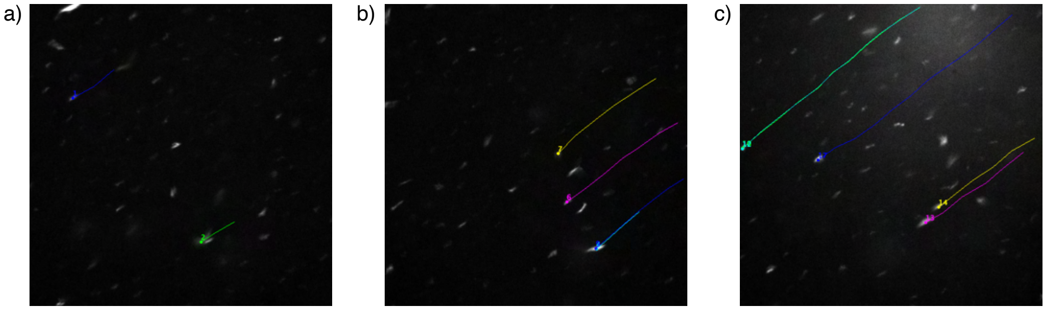

Figure S30. Time-lapse (at 200 ms intervals) CLSM images (82 μm × 82 μm) of a toluene solution of **1** (3.0×10^{-4} M) under the flow condition (λ_{ex} = 488 nm). Some colored lines are tracking lines of the trajectory of particles made by complex **1** (Supplementary Movie S3).

Table S12. The calculated velocity of particles observed in the CLSM images in Figure S30.

34.623	29.296	32.368	36.878	26.629	33.294
32.946	31.486	31.159	31.364	29.762	28.999
29.762	31.292	25.525	30.631	31.86	28.644
28.844	36.389	26.242	37.12	32.179	27.527
30.463	26.242	23.365	24.697	28.307	31.729
21.967	39.856	25.512	35.091	28.408	29.611
24.993	33.38	23.255	28	26.12	31.86
27.551	29.087	27.77	35.063	30.114	32.644
25.525	31.97	29.296	34.733	33.419	34.241
32.486	31.292	29.762	30.631	35.679	34.11
31.729	23.365	28.408	38.006	25.121	30.568
36.486	27.736	22.242	32.368	28.988	28.644
34.11	22.74	23.365	27.586	27.247	32
25.108	24.993	30.87	24.645	27.77	32.653
29.372	26.412	30.88	32.249	31.364	28.375
34.409	27.527	27.863	35.822	28.182	27.736
24.852	29.611	30.568	32.888	30.411	30.242
29.503	32.368	25.96	33.505	30.568	28.856
33.61	26.046	29.437	33.61	30.88	29.087
32.644	28.182	32.179	24.488	31.759	28.844

Average Velocity = 30 ± 4 μm s^{-1}

Table S13. The measured angle of particles observed in the CLSM images in Figure S30.

19.581	15.26	20.327	12.476	9.477
3.687	19.367	13.458	15.198	10.991
5.938	29.074	8.743	14.356	2.445
14.931	11.744	3.98	9.798	3.871
17.358	31.955	15.002	10.358	14.424
36.781	34.492	5.334	6.694	10.43
11.987	31.366	15.43	8.888	13.908
3.635	23.421	5.528	4.284	6.822
11.392	22.566	9.884	30.638	3.488
12.836	17.45	12.017	7.203	32.869
23.337	20.707	6.931	26.779	49.875
14.86	3.162	22.493	8.707	12.325
36.734	14.59	13.031	5.817	14.141
10.048	8.774	0.472	10.313	21.916
15.326	27.399	0.329	1.734	15.546
24.971	32.84	21.897	8.71	AVERAGE
3.939	15.255	5.047	12.708	13.9204947
3.443	7.995	19.784	1.897	STDEV
3.259	9.219	5.909	6.863	9.61088452
12.951	14.036	12.529	0.707	

Average Angle (θ_0) = 14 °

Note: The equation of the 2D order parameter (S) is given as $S = \langle 2(\cos\theta - \theta_0)^2 - 1\rangle$.[69-71] An order parameter of 0.94588 was determined on based on the data in Table S13.

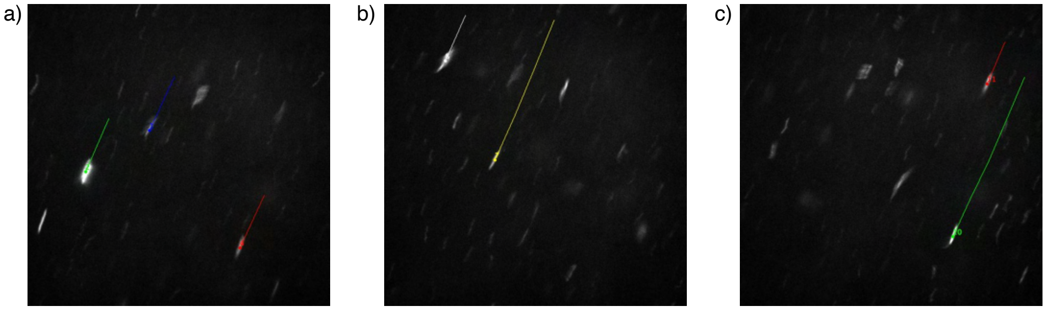

Figure S31. Time-lapse (at 200 ms intervals) CLSM images (82 μm × 82 μm) of a toluene solution of **1** (3.0×10^{-4} M) under the flow condition (λ_{ex} = 488 nm). Some colored lines are tracking lines of the trajectory of particles made by complex **1** (Supplementary Movie S4).

Table S14. The calculated velocity of particles observed in the CLSM images in Figure S31.

80.18	84.076	58.679	55.101
71.956	68.188	55.483	58.405
71.63	71.015	55.886	55.107
78.469	66.607	61.662	
76.219	60.821	69.231	
77.17	57.606	61.574	
78.392	67.338	62.097	
73.111	66.938	60.021	
56.89	68.805	55.823	
64.923	69.231	50.457	
60.847	63.684	57.999	
55.264	67.622	57.21	
57.789	65.97	56.619	
55.483	67.755	56.308	
74.726	54.834	51.262	

Average Velocity = 64 ± 8 μm s^{-1}

Table S15. The measured angle of particles observed in the CLSM images in Figure S31.

2.759	6.226	5.371	5.766
4.302	1.576	3.655	8.13
6.284	1.449	3.685	6.144
6.077	1.331	2.626	AVERAGE
7.211	3.441	3.963	4.84770833
2.544	3.86	3.366	STDEV
11.007	5.434	4.706	2.55401609
14.167	5.778	4.774	
6.776	3.061	10.268	
5.344	7.214	1.055	
3.366	2.367	3.406	
2.337	4.013	4.01	
4.825	4.272	4.57	
7.056	2.36	2.574	
6.414	4.937	6.833	

Average Angle (θ_0) = 5 °

Note: The equation of the 2D order parameter (S) is given as $S = \langle 2(\cos\theta - \theta_0)^2 - 1\rangle$.[69-71] An order parameter of 0.99612 was determined on based on the data in Table S15.

20. Picture of Center-Masked and Margin-Masked Sample Cuvettes, and CD and LD spectra of 1.

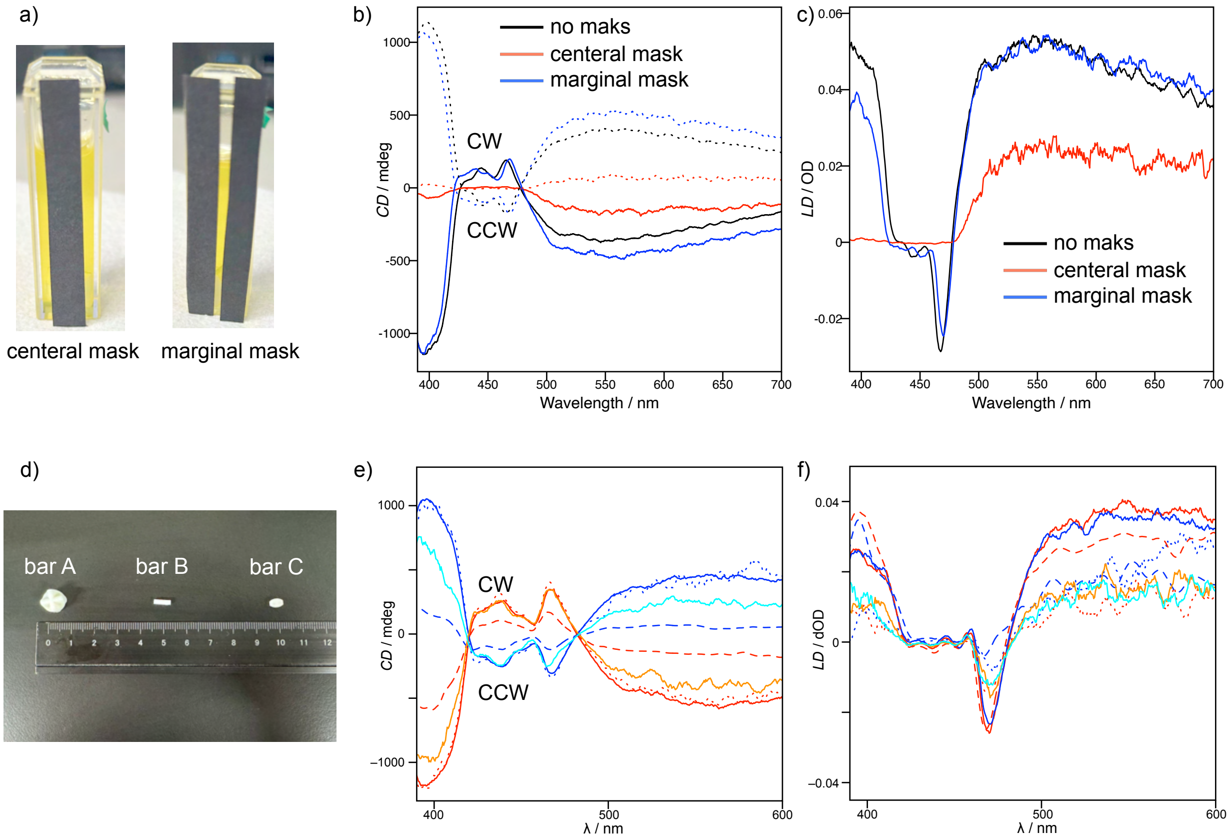

Figure S32. a) Picture of center-masked and margin-masked sample cuvettes. b,c) CD and LD spectra of **1** (3.0×10^{-4} M in toluene) at 298 K with stirring 1000rpm. (d) The image of stirring bars A, B, and C. (e) CD and (f) LD spectra of **1** (3.0×10^{-4} M in toluene) at 298 K with stirring 1000rpm for CW (red curve) and CCW (blue curve) rotations. The solid, dotted, and dashed curves show the CD and LD experiments using stirring bar A, B, and C, respectively. The orange and cyan curves represent the experimental results of CW and CCW rotations, respectively, by stirring bars B and C together.

Note: The influence of vortex flow stability on CD induction was investigated using three differently shaped stir bars (A, B, and C) (Figure S32). Stir bars A and B generated comparable CD and LD signals, whereas the rugby ball-shaped stir bar C produced significantly weaker signals. Simultaneous use of bars B and C resulted in reduced symmetry of the CD and LD signals under clockwise (CW) and counterclockwise (CCW) stirring, highlighting that vortex flow stability is crucial in the CD and LD induction.

21. Flow Velocities of Blue Ink Trails

Table S16. The flow velocities of blue ink trails injected by a syringe pump at stirring rates of 300, 600, 800, and 1000 rpm in Figure 8a (Supplementary Movies S5–S8, respectively).

Rotational velocity / rpm	Flow velocity /μm s^{-1}
300	10,370 ± 8,783
600	23,770 ± 9,281
800	30,441 ± 23,679
1000	40,404 ± 21,222

22. TD-DFT Calculation of Monomer 2

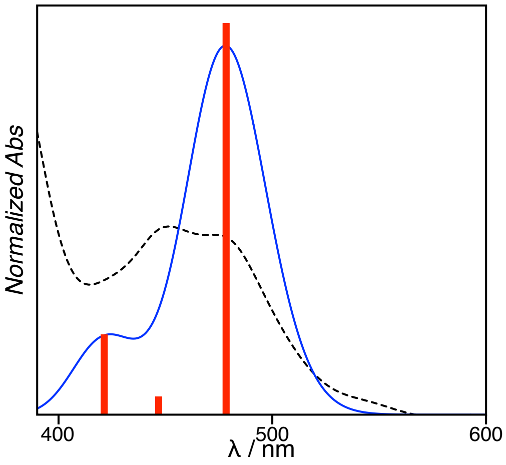

Figure S33. TD-DFT-predicted (M06/6-31g** + LanL2DZ) UV-vis absorption spectra of monomer **2** obtained from the crystal structure (blue solid line) and electron transitions (red lines) overlaid on the experimental UV-vis spectra (Black dashed line) of **1** (3.0×10^{-4} M) in toluene at 363 K. The predicted spectrum was rendered by using a Gaussian band shape of 0.1 eV. A shift factor of –100 nm was applied.

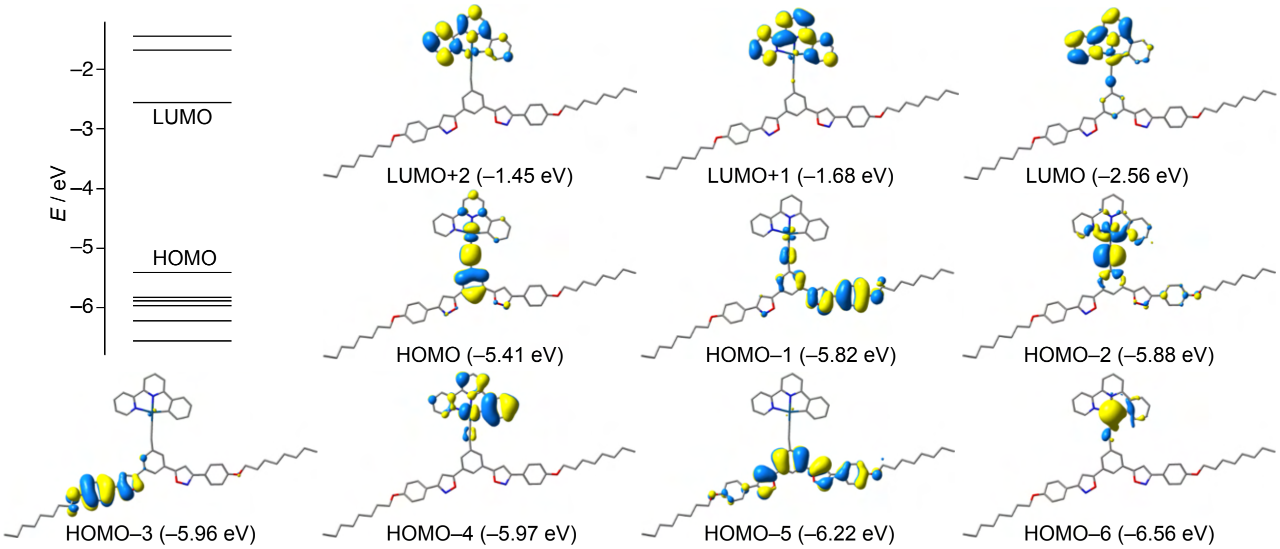

Figure S34. Energy diagram and orbital plots of monomer **2** obtained from the crystal structure calculated by TD-DFT at M06/6-31g** + LanL2DZ level; hydrogen atoms are omitted for clarity; isovalue 0.02.

Table S17. TD-DFT-calculated singlet excited state data for monomer **2** obtained from the crystal structure.†

Excited State	*E* (eV)	*λ* (nm)	*λ* –100 (nm)	*f*	*R* (10^{-40} cgs)
1	2.1436	578.38	478.38	0.1528	23.9023
2	2.2674	546.81	446.81	0.0067	-16.6683
3	2.3780	521.38	421.38	0.0310	-0.5982
4	2.7464	451.43	351.43	0.0018	0.4128
5	2.9275	423.52	323.52	0.0003	-0.0202
6	2.9811	415.91	315.91	0.0435	2.7074
7	3.0699	403.87	303.87	0.0000	0.0120
8	3.1709	391.00	291.00	0.0283	3.4438
9	3.2261	384.31	284.31	0.0019	-0.2416
10	3.2916	376.67	276.67	0.0030	-1.6742

†Excited state energies (*E*), wavelengths (*λ*), oscillator strength (*f*), and rotatory strength (*R*) were calculated at the M06/6-31G** + LanL2DZ level of theory.

Table S18 Electron transitions of the excited states for monomer **2** obtained from the crystal structure.

Exicited state	1	478.38 nm	CI coefficient
HOMO–4	->	LUMO	0.12144
HOMO–2	->	LUMO	0.11184
HOMO	->	LUMO	0.67659

Exicited state	2	446.81 nm	CI coefficient
HOMO–2	->	LUMO	0.67391
HOMO–1	->	LUMO	0.15182

Exicited state	3	421.38 nm	CI coefficient
HOMO–4	->	LUMO	0.67459
HOMO	->	LUMO	-0.12404

Exicited state	4	351.43 nm	CI coefficient
HOMO–6	->	LUMO	0.70253

Exicited state	5	323.52 nm	CI coefficient
HOMO–5	->	LUMO	-0.10737
HOMO–2	->	LUMO	-0.14052
HOMO–1	->	LUMO	0.67999

Exicited state	6	315.91 nm	CI coefficient
HOMO	->	LUMO+1	0.69342

Exicited state	7	303.87 nm	CI coefficient
HOMO–3	->	LUMO	0.69813

Exicited state	8	291.00 nm	CI coefficient
HOMO–4	->	LUMO+1	0.19396
HOMO	->	LUMO+2	0.66039

Exicited state	9	284.31 nm	CI coefficient
HOMO–5	->	LUMO	0.68781

Exicited state	10	276.67 nm	CI coefficient
HOMO–2	->	LUMO+1	0.66678
HOMO–1	->	LUMO+1	0.15088

Exicited state	1	478.38 nm	CI coefficient
HOMO–4	->	LUMO	0.12144
HOMO–2	->	LUMO	0.11184
HOMO	->	LUMO	0.67659

Exicited state	2	446.81 nm	CI coefficient
HOMO–2	->	LUMO	0.67391
HOMO–1	->	LUMO	0.15182

Exicited state	3	421.38 nm	CI coefficient
HOMO–4	->	LUMO	0.67459
HOMO	->	LUMO	-0.12404

Exicited state	4	351.43 nm	CI coefficient
HOMO–6	->	LUMO	0.70253

Exicited state	5	323.52 nm	CI coefficient
HOMO–5	->	LUMO	-0.10737
HOMO–2	->	LUMO	-0.14052
HOMO–1	->	LUMO	0.67999

Exicited state	6	315.91 nm	CI coefficient
HOMO	->	LUMO+1	0.69342

Exicited state	7	303.87 nm	CI coefficient
HOMO–3	->	LUMO	0.69813

Exicited state	8	291.00 nm	CI coefficient
HOMO–4	->	LUMO+1	0.19396
HOMO	->	LUMO+2	0.66039

Exicited state	9	284.31 nm	CI coefficient
HOMO–5	->	LUMO	0.68781

Exicited state	10	276.67 nm	CI coefficient
HOMO–2	->	LUMO+1	0.66678
HOMO–1	->	LUMO+1	0.15088

Table S19. Cartesian coordinates of monomer **2** obtained the crystal structure analysis and employed in a TD-DFT calculation.

Pt	10.2004	5.5749	7.9309
O	1.3112	7.193	9.6653
O	0.737	11.7824	15.8041
O	1.6705	4.0974	5.972
O	2.0717	-0.4045	-0.2151
N	10.8469	6.9514	9.3623
N	12.1601	5.3509	7.8384
N	0.6645	7.9287	10.664
N	1.2437	3.296	4.9501
C	10.0985	7.7426	10.1727
H	9.1514	7.7166	10.0997
C	10.6712	8.5759	11.0889
H	10.1222	9.133	11.6279
C	12.0359	8.6131	11.2395
H	12.4355	9.1865	11.8829
C	12.8112	7.81	10.4416
H	13.7565	7.82	10.5303
C	12.2175	6.9974	9.5201
C	12.9575	6.0851	8.6326
C	14.3153	5.8816	8.5842
H	14.8958	6.3614	9.1645
C	14.8192	4.9761	7.6878
H	15.7606	4.8595	7.6292
C	13.9931	4.2227	6.8595
H	14.3659	3.5901	6.2567
C	12.6456	4.4005	6.924
C	11.5636	3.7514	6.2212
C	11.7839	2.7508	5.2746
H	12.673	2.4814	5.0745
C	10.7417	2.1607	4.6363
H	10.9096	1.4686	4.0066
C	9.4304	2.5534	4.8909
H	8.7028	2.1476	4.434
C	9.2174	3.5383	5.8178
H	8.3224	3.8019	5.9985
C	10.2511	4.1806	6.5171
C	8.2673	5.7277	8.0392
C	7.0509	5.782	8.1557
C	5.6485	5.8003	8.0751
C	4.8445	6.5543	8.9733
H	5.252	7.0387	9.6816
C	3.4621	6.5841	8.8209
C	2.8611	5.8775	7.7972
H	1.9185	5.9158	7.6841
C	3.6358	5.1066	6.9312
C	4.9928	5.0647	7.0944
H	5.5066	4.5132	6.5166
C	2.6334	7.3436	9.7729
C	2.883	8.1949	10.7948
H	3.729	8.5102	11.0888
C	1.6133	8.5174	11.3309
C	1.3428	9.3782	12.4837
C	2.2993	10.2462	12.9714
H	3.1448	10.2892	12.5401
C	2.0677	11.0463	14.0543
H	2.7422	11.6407	14.361
C	0.8552	10.9873	14.7015
C	-0.1408	10.1573	14.2228
H	-0.9922	10.1428	14.6429
C	0.107	9.352	13.1273
H	-0.5747	8.7707	12.8106
C	-0.5104	11.8649	16.4979
H	-0.7967	10.9652	16.7932
H	-1.2079	12.2357	15.9
C	-0.3183	12.7535	17.6723
H	0.1607	13.5695	17.3809
H	0.2532	12.2892	18.3336
C	-1.607	13.1596	18.3428
H	-2.0775	12.351	18.666
H	-2.1919	13.6107	17.6838
C	-1.3539	14.1137	19.535
H	-0.9049	14.9336	19.211
H	-0.751	13.6721	20.1863
C	-2.6891	14.4978	20.245
H	-3.2437	15.0296	19.6209
H	-3.1871	13.6718	20.4652
C	-2.4678	15.301	21.5215
H	-1.9206	14.776	22.1584
H	-1.98	16.1375	21.3121
C	-3.7995	15.631	22.1454
H	-4.3708	14.8239	22.1883
H	-4.268	16.3236	21.6156
C	-3.4667	16.1968	23.6855
H	-2.9184	17.0062	23.624
H	-2.9797	15.5093	24.1866
H	-4.3063	16.4035	24.1452
C	3.0022	4.281	5.8949
C	3.4973	3.6223	4.821
H	4.4024	3.5805	4.5328
C	2.3892	3.0204	4.2294
C	2.326	2.1473	3.0479
C	1.1259	1.8756	2.3989
H	0.3266	2.2821	2.7133
C	1.0697	1.0377	1.316
H	0.2393	0.868	0.8863
C	2.2342	0.4333	0.8462
C	3.4441	0.7114	1.4433
H	4.2469	0.3332	1.1017
C	3.4767	1.5634	2.5638
H	4.3066	1.7394	2.9924
C	3.1388	-1.3279	-0.5056
H	3.4842	-1.7223	0.3351
H	3.8822	-0.8576	-0.9588
C	2.5869	-2.4124	-1.3984
H	2.3101	-2.0153	-2.2628
H	1.7859	-2.8106	-0.9751
C	3.6255	-3.5094	-1.653
H	3.8952	-3.9086	-0.7886
H	4.4307	-3.1053	-2.065
C	3.094	-4.6165	-2.5746
H	2.3935	-5.1285	-2.0967
H	2.6788	-4.2049	-3.3729
C	4.2046	-5.572	-3.0174
H	4.9367	-5.0517	-3.4329
H	4.5722	-6.0362	-2.2238
C	3.698	-6.6004	-4.007
H	3.2416	-6.1275	-4.7468
H	3.0257	-7.1686	-3.5548
C	4.7717	-7.4967	-4.5951
H	5.1766	-8.032	-3.8677
H	5.484	-6.9301	-4.9851
C	4.2443	-8.4431	-5.6744
H	3.8235	-7.9216	-6.3888
H	3.5838	-9.0508	-5.2804
H	4.9879	-8.9636	-6.0433

23. ^{1}H and ^{13}C NMR, and Mass spectra

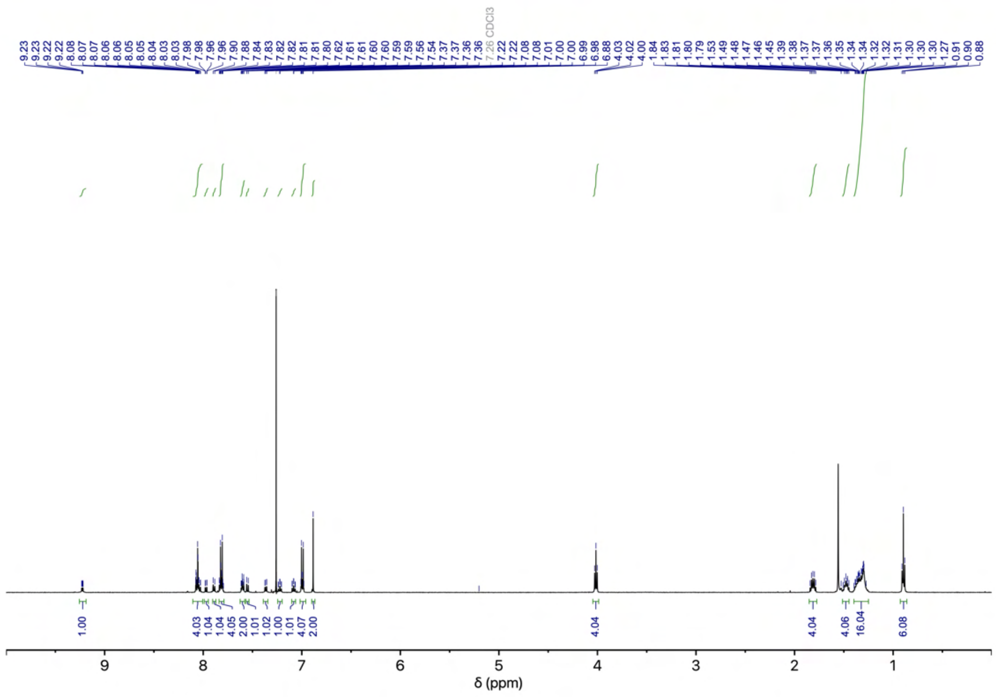

Figure S35. ^{1}H NMR (400 MHz, chloroform-*d*) of 5,5'-(5-ethynyl-1,3-phenylene)bis(3-(4-(octyloxy)phenyl)isoxazole).

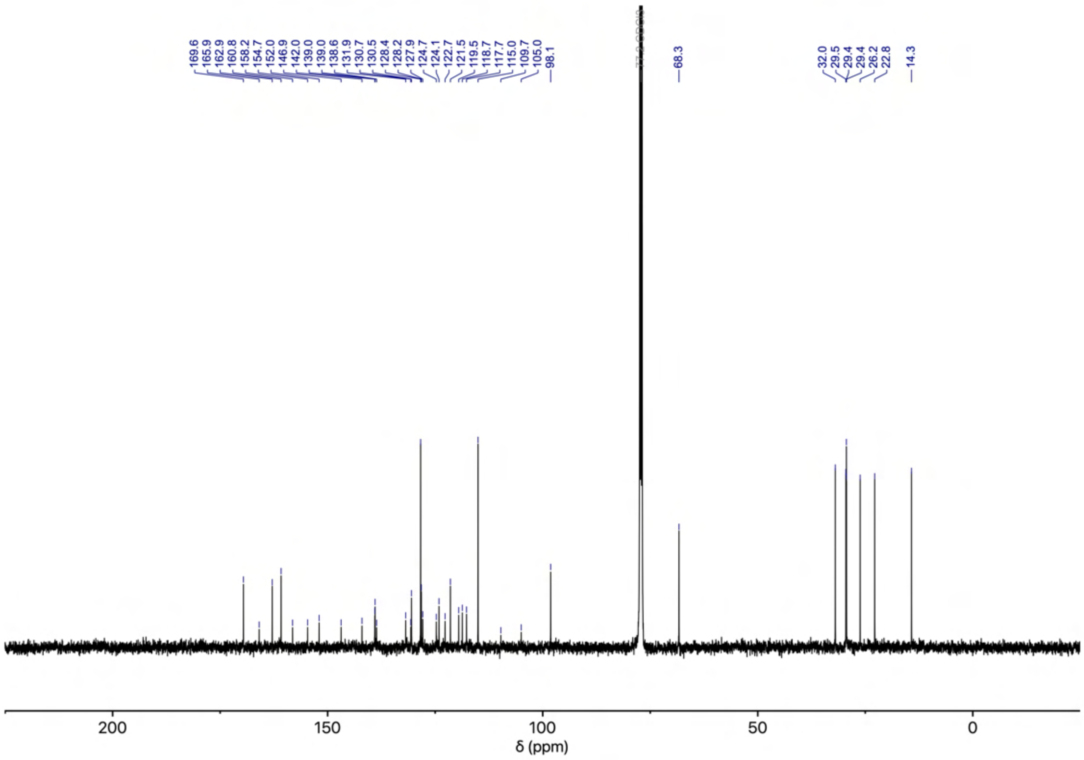

Figure S36. ^{13}C NMR (101 MHz, chloroform-*d*) of 5,5'-(5-ethynyl-1,3-phenylene)bis(3-(4-(octyloxy)phenyl)isoxazole).

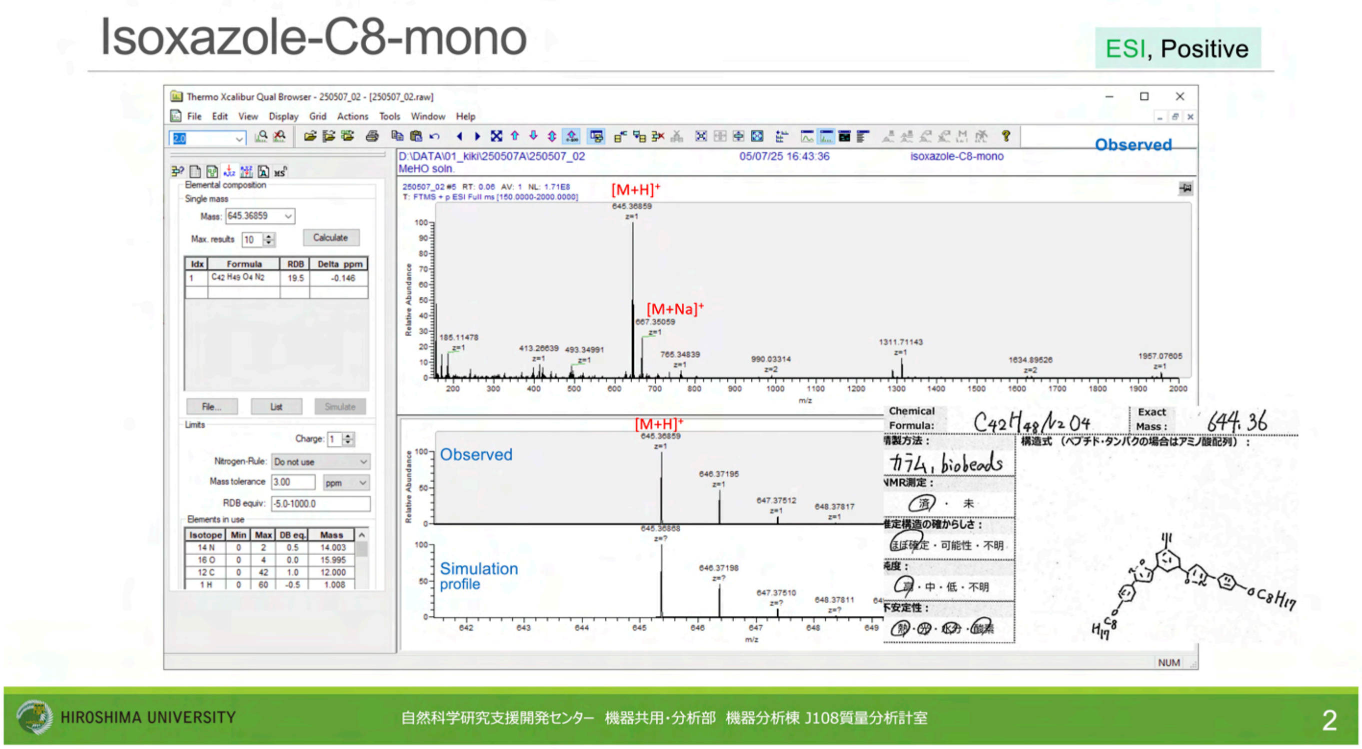

Figure S37. HRMS (ESI-Orbtrap) of 5,5'-(5-ethynyl-1,3-phenylene)bis(3-(4-(octyloxy)phenyl)isoxazole).

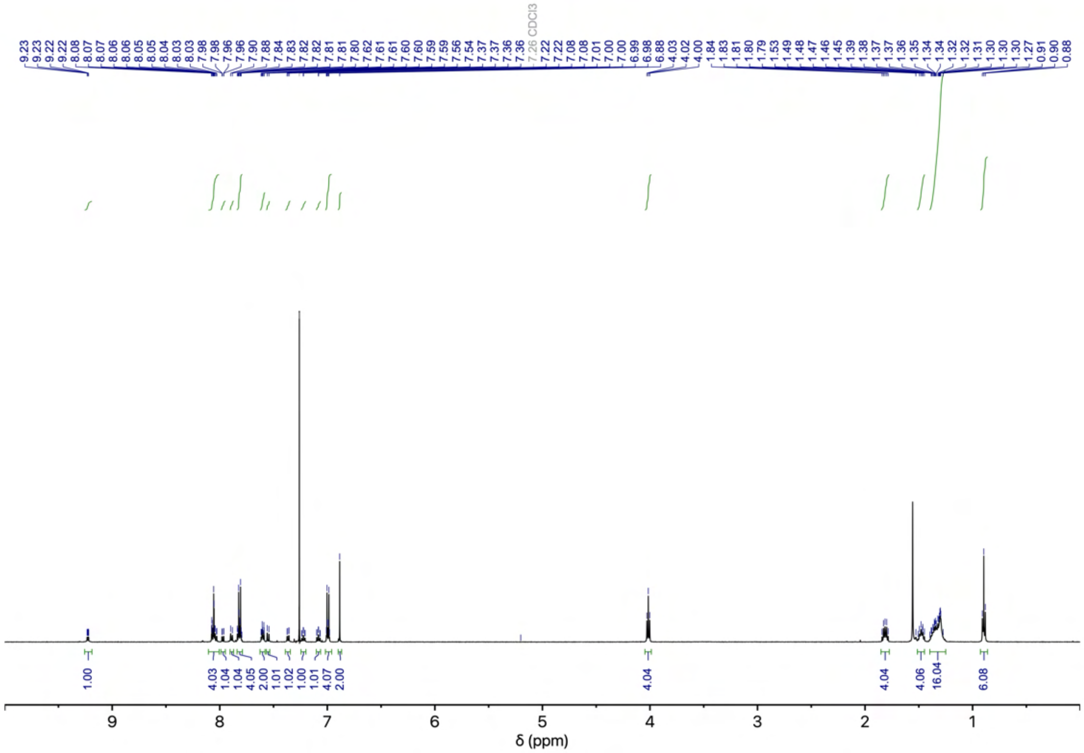

Figure S38. ^{1}H NMR (400 MHz, chloroform-*d*) of compound **2**.

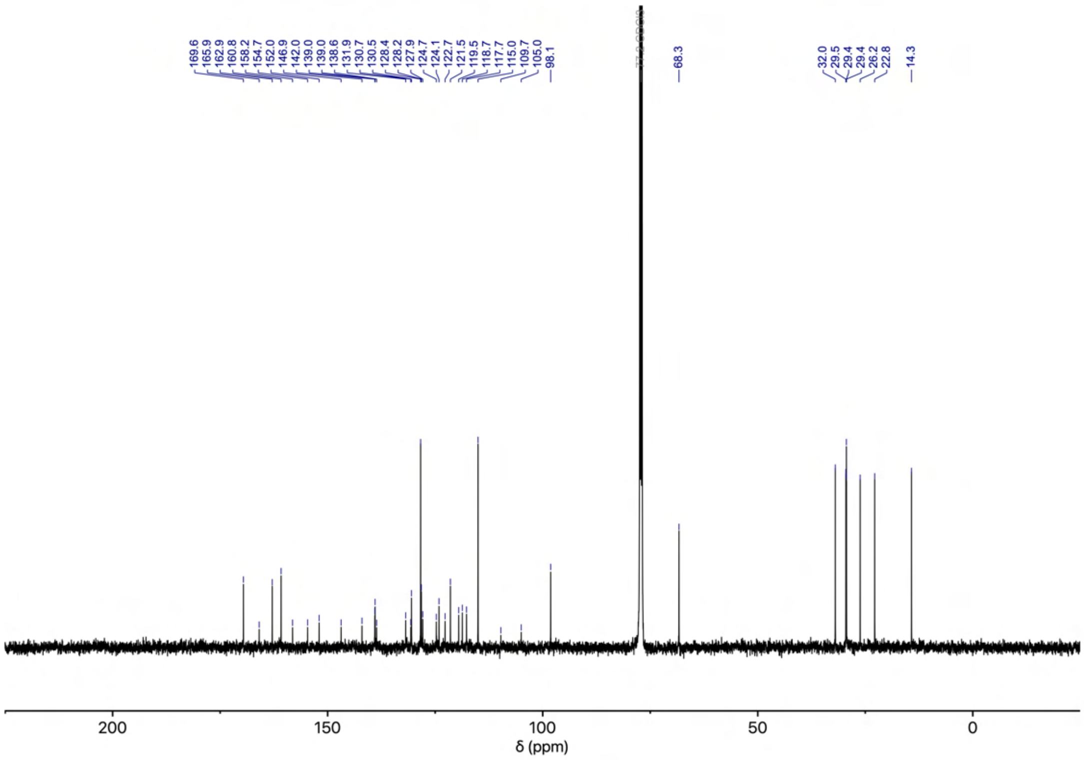

Figure S39. ^{13}C NMR (101 MHz, chloroform-*d*) of compound **2**.

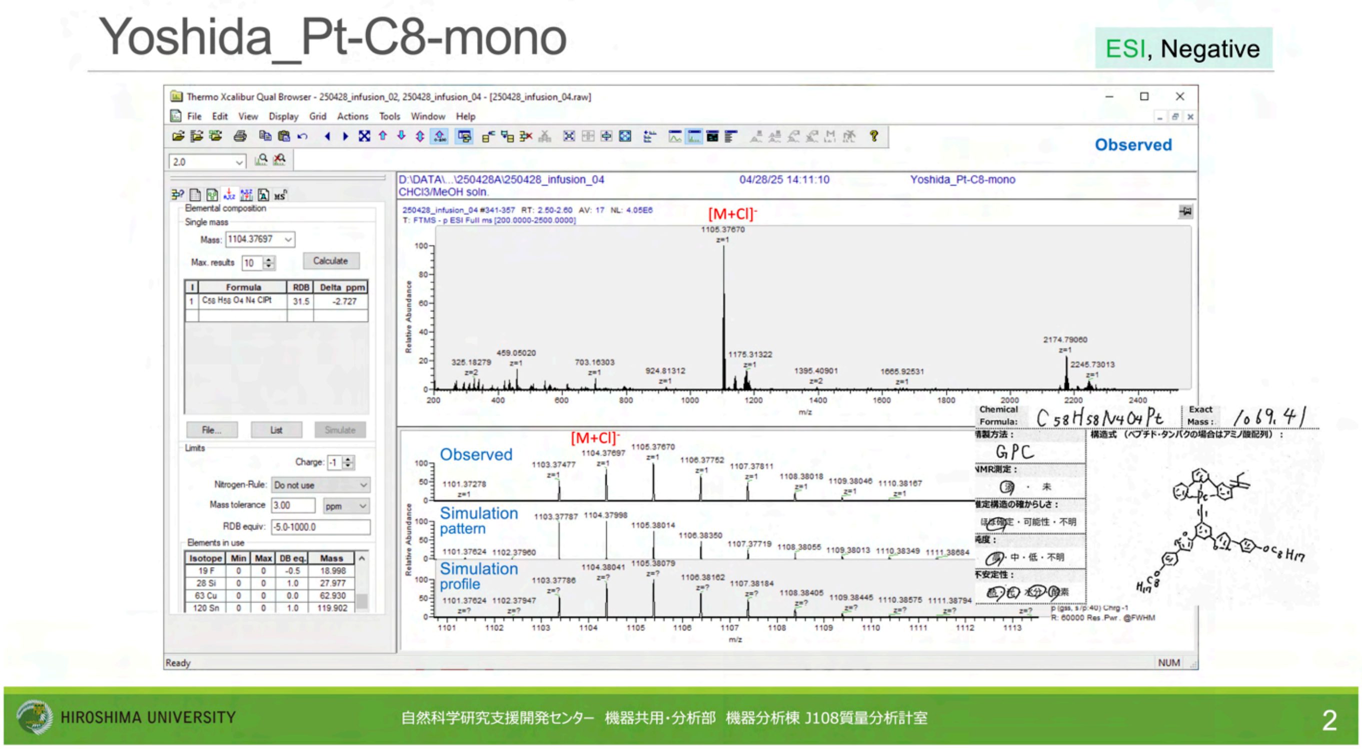

Figure S40. HRMS (ESI-Orbtrap) of compound **2**.

24. Reference

(60) Horcas, I.; Fernández, R.; Gómez-Rodríguez, J. M.; Colchero, J.; Gómez-Herrero, J.; Baro, A. M. WSXM:: A software for scanning probe microscopy and a tool for nanotechnology. *Rev. Sci. Instrum.* **2007**, *78*, 013705.

(61) Gaussian 16, Revision C.01, Frisch, M. J.; Trucks, G. W.; Schlegel, H. B.; Scuseria, G. E.; Robb, M. A.; Cheeseman, J. R.; Scalmani, G.; Barone, V.; Petersson, G. A.; Nakatsuji, H.; Li, X.; Caricato, M.; Marenich, A. V.; Bloino, J.; Janesko, B. G.; Gomperts, R.; Mennucci, B.; Hratchian, H. P.; Ortiz, J. V.; Izmaylov, A. F.; Sonnenberg, J. L.; Williams-Young, D.; Ding, F.; Lipparini, F.; Egidi, F.; Goings, J.; Peng, B.; Petrone, A.; Henderson, T.; Ranasinghe, D.; Zakrzewski, V. G.; Gao, J.; Rega, N.; Zheng, G.; Liang, W.; Hada, M.; Ehara, M.; Toyota, K.; Fukuda, R.; Hasegawa, J.; Ishida, M.; Nakajima, T.; Honda, Y.; Kitao, O.; Nakai, H.; Vreven, T.; Throssell, K.; Montgomery, J. A., Jr.; Peralta, J. E.; Ogliaro, F.; Bearpark, M. J.; Heyd, J. J.; Brothers, E. N.; Kudin, K. N.; Staroverov, V. N.; Keith, T. A.; Kobayashi, R.; Normand, J.; Raghavachari, K.; Rendell, A. P.; Burant, J. C.; Iyengar, S. S.; Tomasi, J.; Cossi, M.; Millam, J. M.; Klene, M.; Adamo, C.; Cammi, R.; Ochterski, J. W.; Martin, R. L.; Morokuma, K.; Farkas, O.; Foresman, J. B.; Fox, D. J. Gaussian, Inc., Wallingford CT, 2016.

(62) Dolomanov, O. V.; Bourhis, L. J.; Gildea, R. J.; Howard, J. A. K.; Puschmann, H. OLEX2: a complete structure solution, refinement and analysis program. *J. Appl. Crystallogr.* **2009**, *42*, 339-341.

(63) Sheldrick, G. M. A short history of SHELX. *Acta Crystallogr A* **2008**, *64*, 112-122.

(64) Sheldrick, G. M. SHELXT - Integrated space-group and crystal-structure determination. *Acta Crystallogr A* **2015**, *71*, 3-8.

(65) Sheldrick, G. M. Crystal structure refinement with SHELXL. *Acta Crystallogr C* **2015**, *71*, 3-8.

(66) Parthasarathy, R. Rapid, accurate particle tracking by calculation of radial symmetry centers. *Nat Methods* **2012**, *9*, 724–726.

(67) Robinson, M. E.; Nazemi, A.; Lunn, D. J.; Hayward, D. W.; Boott, C. E.; Hsiao, M. S.; Harniman, R. L.; Davis, S. A.; Whittell, G. R.; Richardson, R. M.; De Cola, L.; Manners, I. Dimensional Control and Morphological Transformations of Supramolecular Polymeric Nanofibers Based on Cofacially-Stacked Planar Amphiphilic Platinum(II) Complexes. *ACS Nano* **2017**, *11*, 9162-9175.

(68) Sarkar, S.; Sarkar, A.; George, S. J. Stereoselective Seed-Induced Living Supramolecular Polymerization. *Angew. Chem. Int. Ed.* **2020**, *59*, 19841-19845.

(69) Stephen, M. J.; Straley, J. P. Physics of Liquid-Crystals. *Rev. Mod. Phys.* **1974**, *46*, 617-704.

(70) Andrienko, D. Introduction to liquid crystals. *J. Mol. Liq.* **2018**, *267*, 520-541.

(71) Yuan, Y.; Tasinkevych, M.; Smalyukh, I. I. Colloidal interactions and unusual crystallization versus de-mixing of elastic multipoles formed by gold mesoflowers. *Nat. Commun.* **2020**, *11*, 188.
